# S-nitrosylation-mediated coupling of G-protein alpha-2 with CXCR5 induces Hippo/YAP-dependent diabetes-accelerated atherosclerosis

**DOI:** 10.1038/s41467-021-24736-y

**Published:** 2021-07-22

**Authors:** Meng-Lin Chao, Shanshan Luo, Chao Zhang, Xuechun Zhou, Miao Zhou, Junyan Wang, Chuiyu Kong, Jiyu Chen, Zhe Lin, Xin Tang, Shixiu Sun, Xinlong Tang, Hongshan Chen, Hong Wang, Dongjin Wang, Jin-Peng Sun, Yi Han, Liping Xie, Yong Ji

**Affiliations:** 1 Key Laboratory of Cardiovascular and Cerebrovascular Medicine, Nanjing Medical University, Nanjing, China; 2 Key Laboratory Experimental Teratology of the Ministry of Education and Department of Biochemistry and Molecular Biology, School of Basic Medical Sciences, Cheeloo college of Medicine, Shandong University, Jinan, Shandong China; 3 Department of Thoracic and Cardiovascular Surgery, The Affiliated Drum Tower Hospital of Nanjing University Medical School, Nanjing, China; 4 Nanjing Medical University Drum Tower Clinical Medical College, Nanjing, China; 5 The Center for Metabolic Disease Research, Temple University Lewis Katz School of Medicine, Philadelphia, PA USA; 6 Department of Physiology and Pathophysiology, School of Basic Medical Sciences, Peking University, Key Laboratory of Molecular Cardiovascular Science, Ministry of Education, Beijing, China; 7 Department of Geriatrics, First Affiliated Hospital of Nanjing Medical University, Nanjing, Jiangsu China; 8 Key Laboratory of Targeted Intervention of Cardiovascular Disease, Collaborative Innovation Center for Cardiovascular Disease Translational Medicine, Nanjing Medical University, Nanjing, China; 9 State Key Laboratory of Reproductive Medicine, Nanjing Medical University, Nanjing, China

**Keywords:** Nitrosylation, G protein-coupled receptors, Atherosclerosis

## Abstract

Atherosclerosis-associated cardiovascular disease is one of the main causes of death and disability among patients with diabetes mellitus. However, little is known about the impact of S-nitrosylation in diabetes-accelerated atherosclerosis. Here, we show increased levels of S-nitrosylation of guanine nucleotide-binding protein G(i) subunit alpha-2 (SNO-GNAI2) at Cysteine 66 in coronary artery samples from diabetic patients with atherosclerosis, consistently with results from mice. Mechanistically, SNO-GNAI2 acted by coupling with CXCR5 to dephosphorylate the Hippo pathway kinase LATS1, thereby leading to nuclear translocation of YAP and promoting an inflammatory response in endothelial cells. Furthermore, Cys-mutant GNAI2 refractory to S-nitrosylation abrogated GNAI2-CXCR5 coupling, alleviated atherosclerosis in diabetic mice, restored Hippo activity, and reduced endothelial inflammation. In addition, we showed that melatonin treatment restored endothelial function and protected against diabetes-accelerated atherosclerosis by preventing GNAI2 S-nitrosylation. In conclusion, SNO-GNAI2 drives diabetes-accelerated atherosclerosis by coupling with CXCR5 and activating YAP-dependent endothelial inflammation, and reducing SNO-GNAI2 is an efficient strategy for alleviating diabetes-accelerated atherosclerosis.

## Abstract (web summary)

S-nitrosylation can influence many pathophysiological processes. Here the authors show that the coupling efficiency of GNAI2 with CXCR5 is enhanced by S-nitrosylation of GNAI2, leading to Hippo-YAP dysfunction in endothelium, and plays a role in diabetes-accelerated atherosclerosis.

## Introduction

Diabetes mellitus is a major independent risk factor for the development of cardiovascular diseases. Large-scale clinical researches show that atherosclerotic cardiovascular disease remains the principal cause of death among patients with diabetes mellitus^1^. Patients with diabetes have a 2- to 4-fold greater risk of atherosclerosis than nondiabetic individuals^2,3^. In addition, diabetes-accelerated atherosclerosis is associated with 12-fold increase in mortality in men aged between 20 and 29 years, and 7-fold in those between 30 and 39 years^4,5^. More than half of deaths in patients with diabetes are due to ischemic heart disease or heart failure, with advanced atherosclerosis and peripheral artery disease^6^.

Diabetes-accelerated atherosclerosis is a complex disease with manifestations including hyperglycemia, hyperinsulinemia and dyslipidemia, the multiple pathological factors induce oxidative stress, inflammation, and vascular dysfunction^7^. However, the exact mechanisms underlying the acceleration of atherosclerosis in diabetes remain unclear. Several triggers of diabetes, including hyperglycemia, accelerated formation of advanced glycation end-products (AGEs), increased oxidative and nitrosative stress, have been proposed to activate vascular endothelial cells, which is the initial step of diabetes-accelerated atherosclerosis^4,8^. The subsequent secretion of chemokines and adhesion molecules, together with the deposition of platelet-derived chemokines, recruit monocytes to the endothelium and lead to atherosclerotic plaque initiation, formation and rupture^9,10^. Therefore, the inflammatory response is an important hallmark of endothelial dysfunction. In contrast to normoglycemia, hyperglycemia accelerates monocyte and macrophage accumulation in early fatty streak lesions and diapedesis in lesion-prone regions of vasculature, which accelerates necrotic core formation during atherosclerosis^11^.

Nitric oxide (NO) is a gaseous signaling molecule that plays a pivotal role in endothelial homeostasis, and it has an anti-inflammatory effect at the physiological level. However, superfluous NO under pathological conditions induces nitrosative stress and acts as a pro-inflammatory mediator in endothelial cells^12^. Studies have revealed that NO plays diverse pathophysiological roles through protein S-nitrosylation^13^. Protein S-nitrosylation is a covalent post-translational modification of a protein cysteine thiol by a nitric oxide group to form S-nitrosothiol^14^. In general, the NO moiety can be provided by NO itself, which originates from neuronal (nNOS/NOS1), inducible (iNOS/NOS2) and endothelial (eNOS/NOS3) nitric oxide synthase^15^. In contrast, reversible denitrosylation is mediated by S-nitrosoglutathione reductase (GSNOR) and the thioredoxin (Trx) system^16^. S-nitrosylation has been found to play important roles in various cardiovascular diseases^17,18^. It has been reported that the denitrosylation of Serine/threonine kinase (Akt) contributes to vascular inflammation and susceptibility to atherosclerosis^19^. S-nitrosylation of vasodilator-stimulated phosphoprotein (VASP) contributes to increased microvascular permeability. Our previous study also showed that iNOS-induced S-nitrosylation of eNOS at Cys94 and Cys99 aggravates Oxidized low-density lipoprotein (oxLDL)-induced endothelial dysfunction^20^.

Most extracellular stimuli signal through G-protein-coupled receptors (GPCRs) that couple with the heterotrimeric G proteins. GNAI1, GNAI2, and GNAI3 encode subunits of the “inhibitory class” of heterotrimeric G proteins, named based on their ability to inhibit adenylyl cyclase activity^21^. In smooth muscle cells, deficiency of GNAI2 exhibits a phenotype of insulin resistance, while homozygous GNAI2^G184S^ knock-in mice exhibit increased insulin sensitivity^22,23^, indicating the important role of GNAI2 in metabolic diseases. It has been found that S-nitrosylation of G-protein-coupled receptor kinase (GRK) can preserve the beta-adrenergic receptor signaling^24^. In addition, prior analysis has shown that eNOS-derived S-nitrosylation of β-arrestin2 reduces GPCR signals through enhancing receptor internalization^25^. These observations indicate roles of S-nitrosylation of GPCR-affiliated proteins in cellular signaling transduction.

Melatonin (MLT), a highly conserved molecule mostly synthesized in the pineal gland^26^, is well known for its regulatory roles in circadian rhythms, seasonal rhythms^27^, sleep promotion^28^, and immune defense^29^. Aside from these well-known health benefits, melatonin has recently been demonstrated to suppress overactivation of immune function in atherosclerosis and reduce pro-inflammatory cytokine production. These effects have been ascribed, at least in part, to reducing iNOS expression and eliminating redundant NO^30^. Clinical data show that the serum level of melatonin of acute myocardial infarction patients significantly reduces compared to that of control subjects^31^. Moreover, Mutation of the circadian clock gene Per2 impairs endothelial function involving decreased production of NO and increased release of cyclooxygenase-1-driven vasoconstrictor^32,33^, these observations indicate that endothelial function and inflammation are derived by rhythmicity. In addition, melatonin effectively alleviates hyperglycemia-induced MI/R injury by reactivating the intracellular Trx system and Notch1/Hes1/Akt signaling pathway^34^. These observations imply that there might be a complex crosstalk among melatonin, protein S-nitrosylation, and endothelial inflammation.

In this work, we try to find out the role of protein S-nitrosylation in diabetes-accelerated atherosclerosis. Using biotin-switch assay combined with Liquid chromatography-tandem mass spectrometry (LC-MS/MS) for detection of S-nitrosylated proteins and determination of S-nitrosylated sites, we identify GNAI2, which is highly expressed in endothelial cells, is nitrosylated at cysteine 66 in condition of high glucose (HG) and oxLDL. SNO-GNAI2 plays an important role in the initiation and development of diabetes-accelerated atherosclerosis, through inactivating Hippo-Yes-associated protein (YAP) signaling. iNOS mediates the S-nitrosylation of GNAI2. The level of melatonin is decreased in serum of mice with diabetes-accelerated atherosclerosis, and exogenous supplement of melatonin reduces iNOS expression and S-nitrosylation of GNAI2, thus alleviating diabetes-accelerated atherosclerosis.

## Results

### SNO-GNAI2 is higher in diabetes-accelerated atherosclerosis

**Fig. 1:**
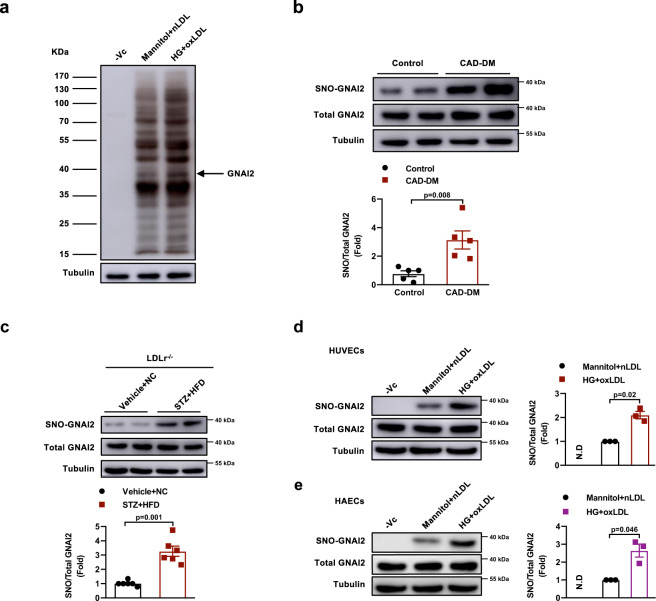
S-nitrosylation of GNAI2 is significantly higher in diabetes-accelerated atherosclerosis in vitro and in vivo. **a** A biotin-switch assay detected protein S-nitrosylation in HUVECs treated with Mannitol+nLDL or HG (25 mmol/L) and oxLDL (50 μg/mL). One independent experiment was performed. **b** S-nitrosylation of GNAI2 is significantly higher in coronary arteries of diabetic patients with CAD. The level of SNO-GNAI2 was quantified with total-GNAI2 and normalized to Control patients. *n* = 5 distinct samples for each group. **c** S-nitrosylation of GNAI2 is increased in the aortas of LDLr^−/−^ mice treated with STZ and HFD. The level of SNO-GNAI2 was quantified with total-GNAI2 and normalized to Vehicle + NC mice. *n* = 6 distinct samples for each group. **d** S-nitrosylation of GNAI2 is markedly increased in HUVECs treated with HG and oxLDL for 24 h. The level of SNO-GNAI2 was quantified with total-GNAI2 and normalized to Mannitol+nLDL-treated cells. *n* = 3 distinct samples for each group. N.D represents no detected. **e** S-nitrosylation of GNAI2 is significantly increased in HAECs treated with HG and oxLDL for 24 h. The level of SNO-GNAI2 was quantified with total-GNAI2 and normalized to Mannitol+nLDL-treated HAECs. *n* = 3 distinct samples for each group. N.D represents no detected. Data are represented as the Mean ± SEM. Unpaired two-tailed Student’s *t*-test was used for statistical analysis. Source data are provided as a Source Data file.

We assessed the total S-nitrosylation in HUVECs exposed to high glucose and oxLDL to mimic the diabetes-accelerated atherosclerosis condition. The whole-cell homogenates were subjected to the biotin-switch assay, the captured proteins were resolved by SDS-PAGE and gels were silver stained (Fig. 1a). With LC-MS/MS analysis, we identified 95 unique proteins. Inhibitory guanine nucleotide-binding protein 2 (GNAI2) was one of the highly S-nitrosylated proteins. To investigate the role of S-nitrosylation of GNAI2 in diabetes-accelerated atherosclerosis, we detected SNO-GNAI2 in coronary artery samples of diabetic patients with coronary artery disease (CAD-DM) and found a significant increase in SNO-GNAI2 rather than total-GNAI2 level, compared to patients with CAD (Fig. 1b). Consistently, the level of SNO-GNAI2 was increased significantly in the aortas of LDLr^−/−^ mice treated with streptozotocin (STZ) and fed on a high fat diet (HFD), compared to corresponding controls (Fig. 1c). In line with this, the level of SNO-GNAI2 was increased only in HUVECs exposed to HG and oxLDL, rather than in cells stimulated with HG or oxLDL alone (Fig. 1d, Supplementary Fig. 1a). Besides, the level of SNO-GNAI2 was higher in human aortic endothelial cells (HAECs) treated with HG and oxLDL than that in HAECs treated with mannitol and nLDL (Fig. 1e). We also detected the levels of S-nitrosylation of GNAI1 and GNAI3, two highly homologous Gi proteins to GNAI2. However, neither GNAI1 nor GNAI3 could be S-nitrosylated upon HG and oxLDL (Supplementary Fig. 1b, c). To explore why only GNAI2 is S-nitrosylated, we analyzed the sequences for these three proteins. Sequence alignment found that the adjacent amino acids for the S-nitrosylated site at Cys66 in GNAI2 is Arg67 (Arginine, R), which is Lys67 (Lysine, K) in GNAI1 and GNAI3 (Supplementary Fig. 1d). Considering that the adjacent amino acids of Cys (Arg67 for GNAI2, and Lys67 for GNAI1 and GNAI3) with the different ability of alkalinity can affect the process of S-nitrosylation, we speculated only GNAI2 with Arg67 could be modified by S-nitrosylation^35–37^. We therefore constructed recombinant plasmid encoding a site-mutated GNAI2 from Arg67 to Lys67 (GNAI2-R67K), which is consistant with GNAI1 and GNAI3. After transfection, GNAI2-R67K significantly reduced the level of GNAI2 S-nitrosylation, indicating that sequence differences surrounding the relevant Cys is important for protein S-nitrosylation (Supplementary Fig. 1e). Taken together, these results suggest that S-nitrosylation of GNAI2 is elevated in diabetes-accelerated atherosclerosis.

### The S-nitrosylation site is Cys66 of GNAI2

**Fig. 2:**
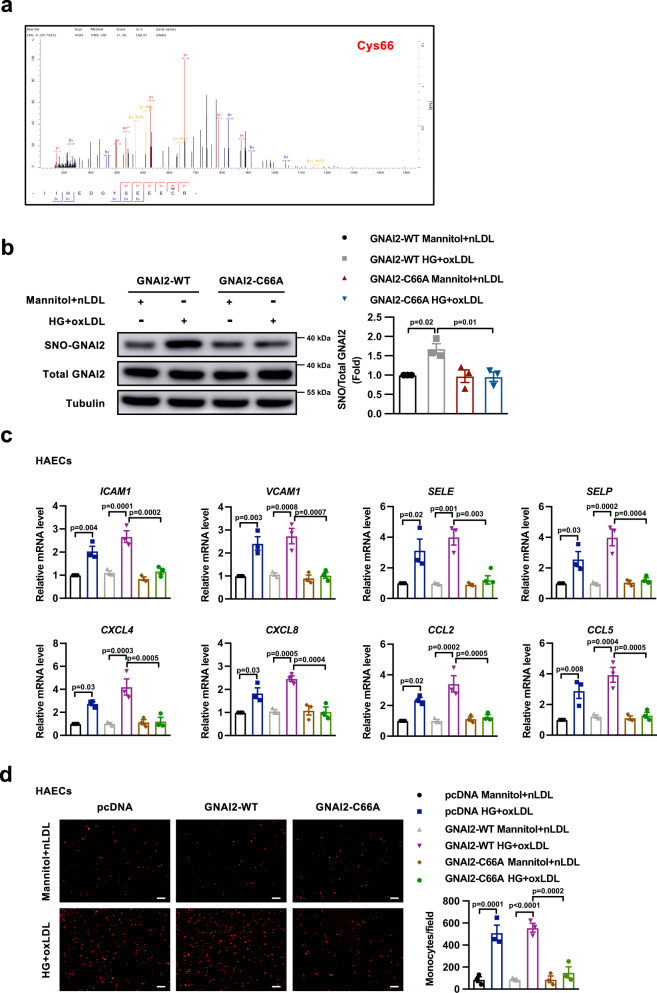
Inhibition of GNAI2 S-nitrosylation at Cys66 alleviates HG- and oxLDL-induced inflammatory response. **a** Cys66 is identified as the SNO-site of GNAI2 according to the liquid chromatography-tandem mass spectrometry (LC-MS/MS) analysis. **b** HUVECs were ectopically expressed with GNAI2-WT and GNAI2-C66A, followed by exposure to HG and oxLDL for 24 h. GNAI2-C66A abolishes S-nitrosylation of GNAI2 induced by HG and oxLDL as determined by a biotin-switch assay. *n* = 3 distinct samples for each group. **c** HAECs were transfected with pcDNA, GNAI2-WT, and GNAI2-C66A, followed by stimulated with HG and oxLDL for 24 h. GNAI2-C66A inhibits the mRNA expressions of adhesion molecules (*ICAM*1, *VCAM1*, *SELE*, and *SELP*) and chemokines (*CXCL4, CXCL8*, *CCL2*, and *CCL5*) induced by HG and oxLDL as determined by qPCR. *n* = 3 distinct samples for each group. **d** GNAI2-C66A prevents the attachment of THP-1 cells to HAECs in HG and oxLDL condition. Scale bar = 100 μm. *n* = 3 distinct samples for each group. Data are represented as the Mean ± SEM. One-way ANOVA followed by Tukey’s test for post-hoc comparisons was used. Source data are provided as a Source Data file.

We then sought to identify the cysteine residue(s) undergoing modification in GNAI2. GNAI2 has 10 cysteine residues, but only one cysteine residue, namely, Cys66, was identified as the SNO-site according to the LC-MS/MS analysis (Fig. 2a). To further determine if Cys66 was the only nitrosylation site of GNAI2, we constructed recombinant plasmid vectors encoding a GNAI2 mutant in which Cys66 was replaced by alanine (GNAI2-C66A) and successfully expressed them in HUVECs and HAECs (Supplementary Fig. 2a, b). As expected, overexpression of GNAI2-C66A inhibited the increase of SNO-GNAI2 induced by HG and oxLDL (Fig. 2b), confirming that Cys66 is the only S-nitrosylation site of GNAI2. Moreover, HG and oxLDL failed to increase the expression of adhesion molecules and chemokines, including Intercellular adhesion molecule 1 (*ICAM1)*, Vascular cell adhesion molecule 1 (*VCAM1)*, E-Selectin (*SELE)*, P-Selectin (*SELP)*, Chemokine C-X-C motif ligand 4 and 8 (*CXCL4*, *CXCL8)*, Chemokine (C-C motif) Ligand 2 (*CCL2)* and *CCL5*, or to enhance the attachment of THP-1 cells to endothelial cells in HAECs and HUVECs expressing SNO-resistant GNAI2-C66A (Fig. 2c, d, Supplementary Fig. 2c, d). These results suggest that S-nitrosylation of Cys66 in GNAI2 leads to endothelial inflammation.

### Cys66 mutation mitigates diabetes-accelerated atherosclerosis

**Fig. 3:**
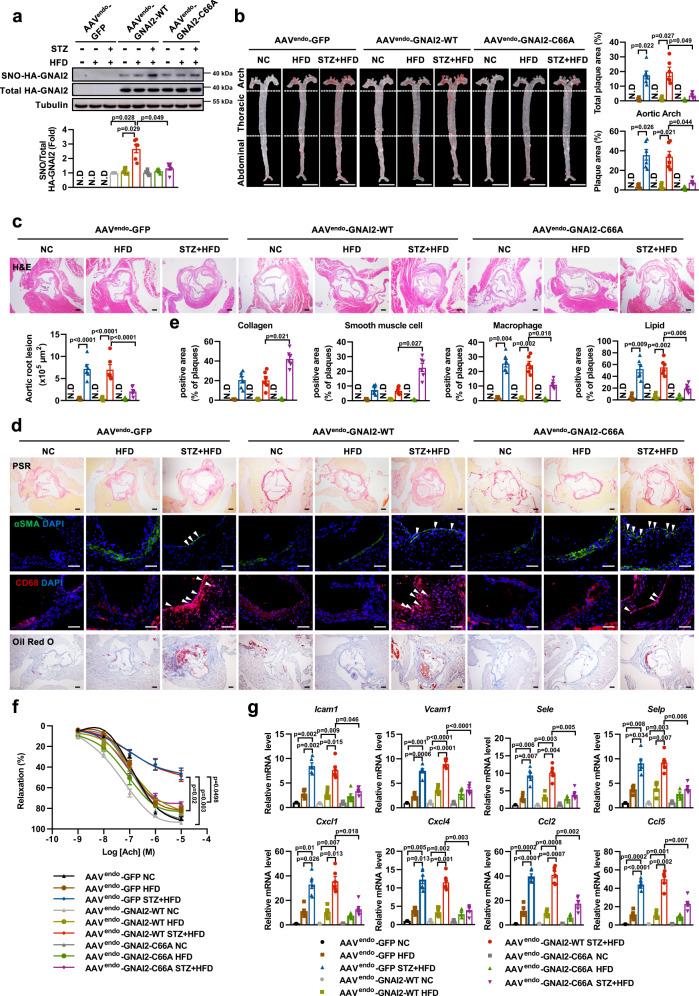
Inhibition of SNO-GNAI2 at Cys66 mitigates endothelial dysfunction in diabetes-accelerated atherosclerosis. LDLr^−/−^ mice were transfected with AAV^endo^-GFP, AAV^endo^-GNAI2-WT, or AAV^endo^-GNAI2-C66A, respectively, and randomly separated into NC, HFD, and STZ + HFD groups. **a** AAV^endo^-GNAI2-C66A significantly diminishes S-nitrosylation of GNAI2 in the aorta, as determined through a biotin-switch assay. *n* = 6 mice for each group. N.D represents no detected. **b** Endothelial specific transfection of GNAI2-C66A alleviates diabetes-accelerated atherosclerosis. Scale bar = 5 mm. *n* = 6 mice for each group. N.D represents no detected. **c** Endothelial specific transfection of GNAI2-C66A decreases plaque areas in the aortic roots, evidenced by hematoxylin and eosin staining (H&E). Scale bar = 200 μm. *n* = 6 mice for each group. N.D represents no detected. **d, e** AAV^endo^-GNAI2-C66A increases the stability of atherosclerotic plaques, as evidenced by picrosirius red staining for collagen (Scale bar = 200 μm), α-SMA for smooth muscle cells (Scale bar = 50 μm), CD68 for macrophage infiltration (Scale bar = 50 μm), and Oil Red O staining for lipid deposition (Scale bar = 200 μm), n = 6 mice for each group. N.D represents no detected. **f** Endothelial specific transfection of GNAI2-C66A rescues the impairment in endothelium-dependent relaxation induced by STZ and HFD in the presence of Ach (10^−9^ to 10^−5^ mol/L). *n* = 6 mice for each group. **g** The mRNA expressions of adhesion molecules (*Icam1*, *Vcam1*, *Sele*, and *Selp*) and chemokines (*Cxcl1*, *Cxcl4*, *Ccl2*, and *Ccl5*) are significantly reduced in aortas of STZ and HFD-treated LDLr^−/−^ mice transfected with AAV^endo^-GNAI2-C66A. *n* = 6 mice for each group. Data are represented as the Mean ± SEM. **a, b, e, f, g** Welch ANOVA followed by Tamhane’s T2 test for post-hoc comparisons. **c** One-way ANOVA followed by Tukey’s test for post-hoc comparisons. Source data are provided as a Source Data file.

We next investigated the role of SNO-GNAI2 in the development of diabetes-accelerated atherosclerosis lesions in vivo. LDLr^−/−^ mice were transduced with the EC-enhanced AAV vector containing GFP (AAV^endo^-GFP), GNAI2-WT (AAV^endo^-GNAI2-WT), or GNAI2-C66A (AAV^endo^-GNAI2-C66A), respectively, and subjected to normal chow (NC), HFD and STZ + HFD treatments for 4 weeks. Body weight, blood glucose, and metabolic parameters are shown in Supplementary Table 4. The body weights were significantly reduced in STZ + HFD-treated LDLr^−/−^ mice, and plasma lipid levels including total cholesterol (TC), Triglyceride (TG) and low-density lipoprotein (LDL) were significantly increased in HFD- and STZ + HFD-treated LDLr^−/−^ mice, while the fasting blood glucose were only increased in STZ + HFD-treated LDLr^−/−^ mice. Transduction with GNAI2-C66A showed no effects on these metabolic parameters compared to AAV^endo^-GNAI2-GFP or AAV^endo^-GNAI2-WT. Transduction with GNAI2-C66A significantly attenuated STZ + HFD-induced increase of SNO-GNAI2 (Fig. 3a). Functionally, we confirmed that STZ + HFD-treated LDLr^−/−^ mice developed obvious atherosclerosis, while little atherosclerotic lesions were found in NC- or HFD-treated LDLr^−/−^ mice (Fig. 3b, c). These results were in accordance with what observed by Deng, J. N, et al.^38^ AAV^endo^-GNAI2-C66A enhanced plaque stability compared to AAV^endo^-GFP and AAV^endo^-GNAI2-WT in STZ + HFD-treated LDLr^−/−^ mice, as evidenced by smaller necrotic cores, thicker fibrous cap (Supplementary Fig. 3a, b), increased collagen and smooth muscle cells content and reduced macrophage infiltration and lipid deposition (Fig. 3d, e). Besides, in aorta roots from STZ + HFD-treated LDLr^−/−^ mice transduced with AAV^endo^-GNAI2-C66A, we observed a significant reduction in intraplaque hemorrhage (Supplementary Fig. 3c). Endothelial specific transfection of GNAI2-C66A decreased the profibrous markers including type IV collagen a (*Col4a)*, *Fibronectin*, connective tissue growth factor *(Ctgf)* at the mRNA levels (Supplementary Fig. 3d), indicating reduced vascular fibrosis and remodeling. In addition, we assessed Ach-mediated aortic rings vasodilation, as expected, AAV^endo^-GNAI2-C66A rescued the impairment in endothelium-dependent relaxation ability, compared to AAV^endo^-GFP or AAV^endo^-GNAI2-WT (Fig. 3f). The mRNA expressions of monocytes rolling and adhesion molecules, including *Sele*, *Selp*, *Icam1*, and *Vcam1* were increased in aortas of STZ + HFD-treated LDLr^−/−^ mice and were reduced by AAV^endo^-GNAI2-C66A. Consistently, the elevation of chemokines including *Cxcl1*, *Cxcl4*, *Ccl2*, and *Ccl5* were attenuated by AAV^endo^-GNAI2-C66A (Fig. 3g). Taken together, these data demonstrate that inhibition of SNO-GNAI2 at Cys66 mitigates diabetes-accelerated atherosclerosis.

### SNO-GNAI2 induces CXCR5 activation and Hippo-YAP dysfunction

**Fig. 4:**
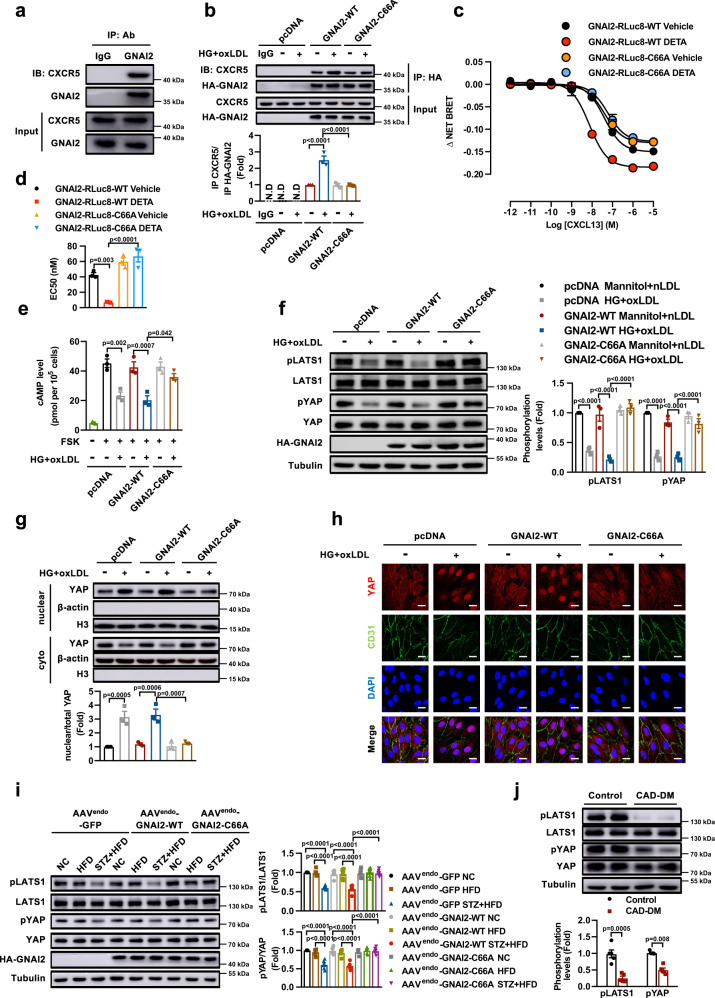
S-nitrosylation of GNAI2 at Cys66 mediates CXCR5 activation and Hippo-YAP pathway dysfunction. **a** Interaction of endogenous GNAI2 with CXCR5. Three indepenent experiments were performed. **b** GNAI2-C66A markedly decreases the interaction between GNAI2 and CXCR5 induced by HG + oxLDL. *n* = 3 distinct samples for each group. N.D represents no detected. **c, d** The BRET assay shows that GNAI2-C66A reverses the reduced EC50 of CXCL13 induced by treatment with DETA. *n* = 3 distinct samples for each group. **e** Ectopic expression of GNAI2-C66A rather than GNAI2-WT partly inhibits the reduction of cAMP in HUVECs stimulated with HG + oxLDL. *n* = 3 independent experiments. **f** Overexpression of GNAI2-C66A restores the decrease of phospho-LAST1 kinase and phospho-YAP induced by HG and oxLDL. *n* = 3 distinct samples for each group. **g** Overexpression of GNAI2-C66A reduces the nuclear level of YAP in HUVECs stimulated with HG + oxLDL. *n* = 3 distinct samples for each group. **h** Accumulation of YAP (Red) in nuclei (Blue) is reduced in HUVECs ectopically overexpressed with GNAI2-C66A. HUVECs were stained with CD31 (Green). Scale bar = 20 μm. Three indepenent experiments were performed. **i** Endothelial specific transduction of GNAI2-C66A rescues the dephosphorylation of LATS1 and YAP induced by STZ and HFD in LDLr^−/−^ mice. *n* = 6 distinct samples for each group. **j** Compared to CAD patients, phosphorylated levels of LATS1 and YAP are reduced in coronary arteries of diabetic patients with CAD. *n* = 5 distinct samples for each group. Data are represented as the Mean ± SEM. **b, d, e, f, g, i** One-way ANOVA followed by Tukey’s test for post-hoc comparisons. **j** Unpaired two-tailed Student’s *t*-test for pLATS1 and Mann–Whitney test for pYAP. Source data are provided as a Source Data file.

We next investigated the involvement of potential Gi-coupled GPCRs operating upstream of GNAI2 in regulation of SNO-GNAI2-induced endothelial inflammation. According to the RNA-seq analysis for the expression of Gi-coupled GPCRs in HUVECs, we performed an siRNA-mediated knockdown of top 14 highly abundant primary Gi receptors in HUVECs (Supplementary Fig. 4a), and determined the level of *ICAM1*, *VCAM1*, *SELE*, and *CCL2* upon HG and oxLDL. Either knockdown of the C-X-C chemokine receptor (CXCR) 4 or 5 could inhibit the increases in the four pro-inflammatory factors, which were significantly increased in HUVECs after HG and oxLDL treatment (Supplementary Fig. 4b, c). C-X-C chemokine receptor (CXCR) is a class of Gi-coupled GPCRs implicated in regulation of cytokine–cytokine receptor interaction. We thus further investigated the effects of SNO-GNAI2 on CXCR4 and CXCR5 signaling. The endogenous interaction of GNAI2 with CXCR5 rather than CXCR4 was observed by coimmunoprecipitation under normal conditions (Fig. 4a, Supplementary Fig. 4d). CXCR5, a chemokine receptor belonging to the GPCRs family, is involved in lymphocyte homing and development of normal lymphoid tissue^39,40^. Most signal transduction by CXCR5 requires recruitment of intracellular G proteins^41^. HG and oxLDL increased the interaction between GNAI2 and CXCR5 in HUVECs transfected with GNAI2-WT, however, overexpression of GNAI2-C66A markedly reversed such binding (Fig. 4b). Importantly, to study the effects of SNO-GNAI2 on CXCR5 signaling, we applied the bioluminescence resonance energy transfer (BRET) assay to determine the coupling of CXCR5 to GNAI2 in living HEK293T cells transiently transfected with GNAI2-RLuc8-WT or GNAI2-RLuc8-C66A, together with Gβ and Gγ-GFP2. Cells were then treated with Chemokine C-X-C motif ligand 13 (CXCL13) for 5 min before determining BRET signaling. The dose-response curve showed that the addition of DETA, a NO donor, which can raise the level of SNO-GNAI2, significantly increased the coupling of CXCR5 to GNAI2 in cells overexpressing GNAI2-WT rather than GNAI2-C66A (Fig. 4c, d). Ectopic expression of GNAI2-WT partially reduced the level of cAMP and GNAI2-C66A restored this reduction, indicating a ligand-independent effect of SNO-GNAI2 (Fig. 4e). In contrast, the coupling between CXCR4 and GNAI2 was not affected by S-nitrosylation (Supplementary Fig. 4e). These observations imply that SNO-GNAI2 specifically couples with CXCR5 and activates CXCR5 signaling in HUVECs.

GPCR signaling conveys extracellular stimuli to a variety of intracellular effectors, cAMP/Protein kinase (PKA)-mitogen-activated protein kinases (MAPKs) and phospholipase C (PLC)- phosphatidylinositol-3 kinase (PI3K)-Akt are the most reported downstream pathways^42^. Studies in HUVECs exposed to HG and oxLDL have revealed no change in activation of the Akt/ mammalian target of rapamycin (mTOR) pathway, and although there was a significant increase in the phosphorylation of ERK1/2 with HG and oxLDL treatment, ectopic overexpression of GNAI2-C66A failed to block Extracellular-signal-regulated kinase (ERK) phosphorylation (Supplementary Fig. 4f, g, h). These data imply that there are other downstream effectors of SNO-GNAI2 in the regulation of endothelial inflammation. The Hippo pathway has been established as a critical signaling branch downstream of GPCRs, and it has been reported to modulate endothelial activation and vascular inflammation in different conditions^43,44^. Activation of the Hippo pathway leads to downstream effector, YAP phosphorylation at S127 and its retention in the cytoplasm, thus suppressing transcriptional activity of YAP. In contrast, Hippo pathway inactivation induces YAP translocation into the nucleus and elicits YAP transcriptional activity^45^. To determine if Hippo signaling is involved in the pro-inflammatory effects of SNO-GNAI2, we detected the activity of the Hippo pathway in HUVECs transfected with GNAI2-WT and GNAI2-C66A. We found that HG and oxLDL led to dysfunction of the Hippo signaling pathway as evidenced by dephosphorylation of large tumor suppressor kinase 1 (LAST1) kinase and YAP (Fig. 4f) along with nuclear translocation of YAP (Fig. 4g, h). However, activation of YAP was reduced by overexpression of GNAI2-C66A rather than GNAI2-WT in HUVECs treated with HG and oxLDL (Fig. 4f, g, h). Consistently, the result was observed in STZ and HFD-treated LDLr^-/-^ mice transfected with AAV^endo^-GNAI2-WT and AAV^endo^-GNAI2-C66A (Fig. 4i). More importantly, dephosphorylation of LATS1 and YAP were observed in coronary artery samples of diabetic patients with CAD, compared with CAD patients (Fig. 4j). Collectively, these results demonstrate that Hippo-YAP is a key downstream effector of CXCR5, and inhibition of SNO-GNAI2 mitigates the dysfunction of Hippo-YAP pathway.

### Inhibiting CXCR5-Hippo-YAP reduces endothelial inflammation

**Fig. 5:**
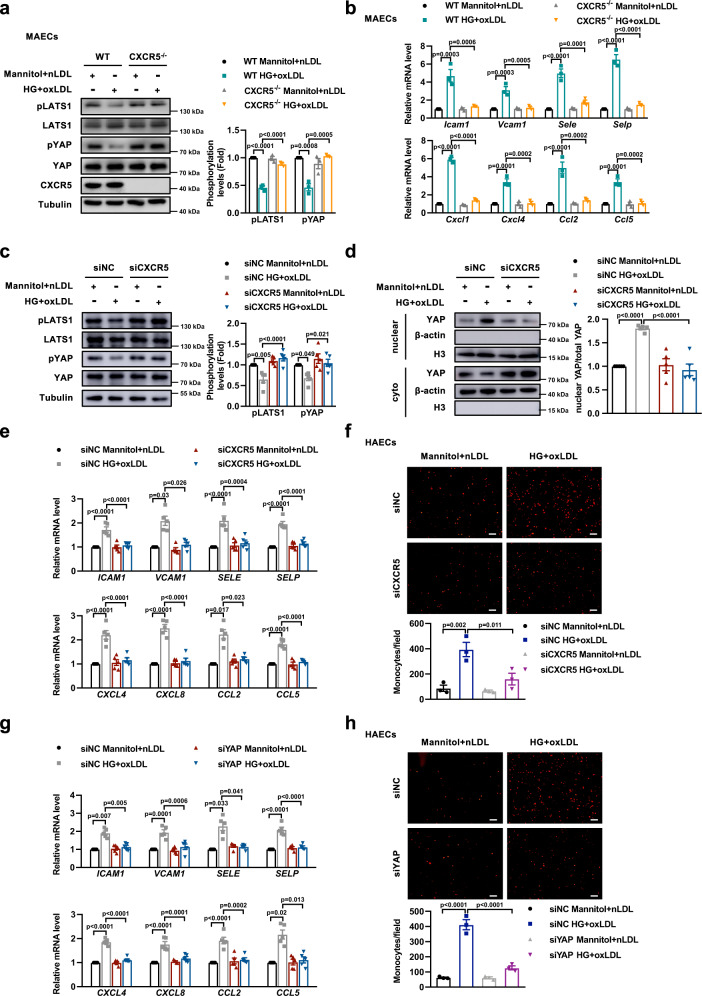
Inactivation of CXCR5-Hippo-YAP pathway improves endothelial inflammation and monocyte adhesion induced by HG and oxLDL. **a** MAECs isolated from CXCR5^−/−^ mice were treated with HG + oxLDL for 24 h. Knockout of CXCR5 reverses the decreased phosphorylation of LATS1 and YAP induced by HG + oxLDL. *n* = 3 distinct samples for each group. **b** Knockout of CXCR5 reduces the expression of adhesion molecules and chemokines. *n* = 3 independent experiments. **c** HUVECs were transfected with siNC or siCXCR5, subjected to HG and oxLDL for 24 h, the level of phospho-LAST1 and phospho-YAP was determined. Knockdown of CXCR5 restores the phosphorylation of LATS1 and YAP, **d** reduces YAP nuclear translocation, **e** suppresses the expression of adhesion molecules and chemokines. **c–e** *n* = 5 distinct samples for each group. **f** Knockdown of CXCR5 reduces the attachment of THP-1 monocytes to HAECs treated with HG and oxLDL. Scale bar = 100 μm. *n* = 3 distinct samples for each group. **g** HUVECs were transfected with siNC or siYAP, followed by stimulated with HG and oxLDL. Knockdown of YAP attenuates the mRNA expression of adhesion molecules and inflammatory chemokines. *n* = 5 distinct samples for each group. **h** Knockdown of YAP decreases the attachment of THP-1 cells to HG- and oxLDL-stimulated HAECs. *n* = 3 distinct samples for each group. Data are represented as the Mean ± SEM. *VCAM1*, *CCL2* in **e** and *ICAM1*, *SELE* in **g** were analyzed by Welch ANOVA followed by Tamhane’s T2 test for post-hoc comparisons. Others were analyzed by One-way ANOVA followed by Tukey’s test for post-hoc comparisons. Source data are provided as a Source Data file.

To further confirm that CXCR5 promotes YAP activation and induces inflammation, we isolated aortic endothelial cells from CXCR5^-/-^ mice (MAECs) or WT littermates, and stimulated them with HG + oxLDL. As expected, CXCR5 deficiency restored the reduction of phosphorylation of LATS1 and YAP induced by HG and oxLDL and reversed the elevation of inflammatory factors (Fig. 5a, b). We then transfected HUVECs with silencing RNAs against CXCR5 (siCXCR5) (Supplementary Fig. 5a), As shown in Fig. 5c and d, knockdown of CXCR5 restored the Hippo signaling pathway in HG- and oxLDL-treated HUVECs. As a result, the expression of adhesion molecules and chemokines, and attachment of THP-1 monocytes to ECs were reduced (Fig. 5e, f, Supplementary Fig. 5b). Besides, we used a Gi protein inhibitor, pertussis toxin (PTX), to inactivate CXCR5-GNAI2 signaling. As expected, PTX reversed the activation of Hippo pathway and reduced the expression of inflammatory genes in HUVECs stimulated with HG and oxLDL (Supplementary Fig. 5c, d). Similarly, knockdown of LATS1 significantly enhanced phosphorylation of YAP and led to retention of YAP in the cytoplasm (Supplementary Fig. 5e, f, g). Either LAST1 or YAP deficiency attenuated the expression of adhesion molecules and inflammatory chemokines upon HG and oxLDL, and this deficiency reduced monocyte adherence to ECs (Supplementary Fig. 5h–l, Fig. 5g, h). Considering that S-nitrosylation of GNAI2 promotes its coupling with CXCR5, these data collectively confirm that S-nitrosylation of GNAI2 induces the inflammatory response through the CXCR5-Hippo-YAP signaling pathway.

### Increased iNOS promotes SNO-GNAI2

**Fig. 6:**
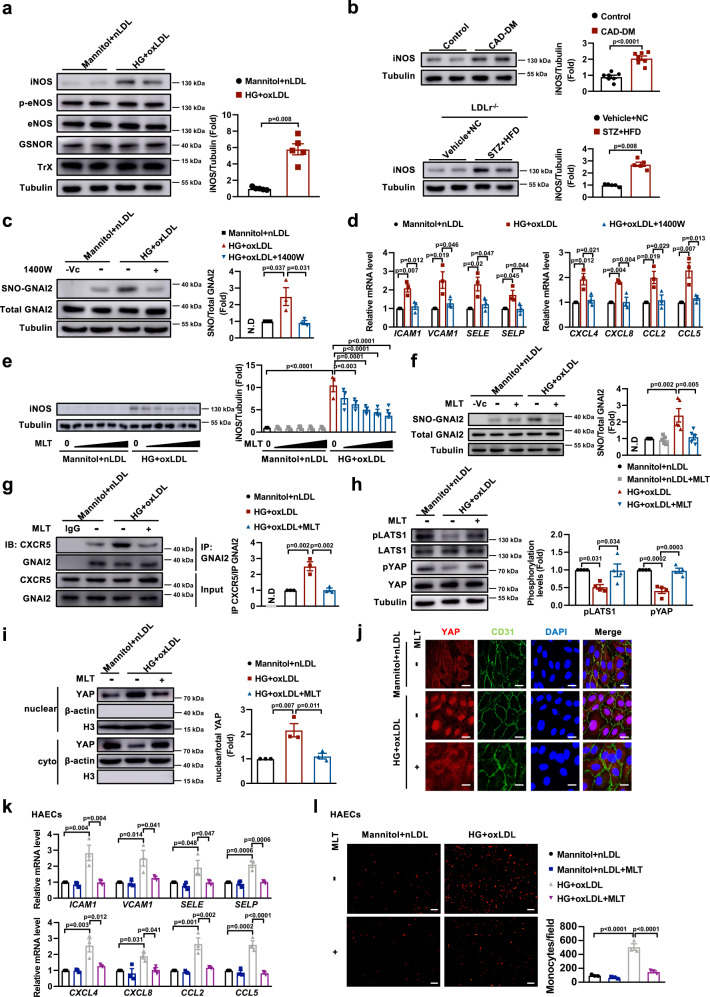
iNOS is responsible for melatonin-induced inhibition of SNO-GNAI2 and melatonin suppresses endothelial inflammation through CXCR5/Hippo pathway. **a** HG + oxLDL increases the level of iNOS, rather than eNOS, phospho-eNOS, GSNOR, or Trx. *n* = 5 cell samples for each group. **b** The expression of iNOS is increased in coronary arteries of diabetic patients with CAD and in aortas of STZ + HFD-treated LDL^−/−^ mice. *n* = 7 human samples. *n* = 5 mice samples. **c** 1400 W (100 μM) pretreatment for 15 min abolishes the elevation of SNO-GNAI2 induced by HG + oxLDL in HUVECs. *n* = 3 independent experiments. N.D represents no detected. **d** 1400 W represses the mRNA expressions of adhesion molecules and chemokines. *n* = 3 independent experiments. **e** Melatonin dose-dependently (1 μM, 2 μM, 5 μM, 10 μM, 20 μM) reduces the level of iNOS in HG + oxLDL-treated HUVECs. *n* = 3 independent experiments. **f** Melatonin decreases S-nitrosylation of GNAI2 in HG + oxLDL-treated HUVECs. *n* = 5 independent experiments. N.D represents no detected. **g** Melatonin represses the interaction between GNAI2 and CXCR5 (*n* = 3 independent experiments, N.D represents no detected), **h** reverses the dephosphorylation of LATS1 and YAP (*n* = 4 independent experiments) and **i** reduces the nuclear translocation of YAP in HUVECs treated with HG + oxLDL (*n* = 3 independent experiments). **j** Melatonin reduces nuclear translocation of YAP. YAP (red), nuclei (DAPI, blue), and HUVECs (CD31, green). Scale bar = 20 μm. Three indepenent experiments were performed. **k** Melatonin abolishes the elevation of adhesion molecules and chemokines in HAECs induced by HG + oxLDL. **l** Melatonin attenuates the adhesion of THP-1 cells to HAECs. Scale bar = 100 μm. **k, l** *n* = 3 independent experiments. Data are represented as the Mean ± SEM. **a** Mann–Whitney test. **b** Mann–Whitney test for mice samples and Unpaired two-tailed Student’s *t*-test for human samples. **c, d, f, g, h, i, k, l** One-way ANOVA followed by Tukey’s test for post-hoc comparisons. **e** One-way ANOVA followed by Dunnett’s test for post-hoc comparisons. Source data are provided as a Source Data file.

The sources of nitrosylating groups might include NOx species that originate exogenously or endogenously^16^. Therefore, we next explored the upstream enzymes that mediated S-nitrosylaiton of GNAI2. Compared to HUVECs treated with mannitol and nLDL (referred to as control), HG and oxLDL increased the protein expression of iNOS, whereas the expressions of phosphorylated eNOS, total eNOS, GSNOR, and Trx did not change (Fig. 6a). Meanwhile, the protein level of iNOS was increased in coronary artery samples of diabetic patients with CAD and in aortic samples of STZ and HFD-treated LDLr^−/−^ mice (Fig. 6b). To further validate whether S-nitrosylation of GNAI2 is mainly mediated by iNOS, we pretreated HUVECs with an iNOS inhibitor named 1400 W. As expected, 1400 W treatment decreased the level of SNO-GNAI2 induced by HG and oxLDL (Fig. 6c). Moreover, inhibition of iNOS abolished the increase in expression of adhesion molecules and inflammatory chemokines in HUVECs treated with HG and oxLDL (Fig. 6d). Taken together, these results indicate that the nitrosylation of GNAI2 is dependent on iNOS.

### Melatonin suppresses SNO-GNAI2 through reducing iNOS

Melatonin, a neuroendocrine hormone, which is synthesized in the pineal gland and other organs, has been shown to prevent endothelial inflammation in atherosclerosis^46^. We observed that the serum levels of melatonin in mice with diabetes-accelerated atherosclerosis were significantly lower than those in controls (Supplementary Fig. 7a). Given that S-nitrosylation of GNAI2 is dependent on iNOS, we detected the effects of melatonin on iNOS and found that melatonin dose-dependently reduced the protein level of iNOS in HUVECs stimulated with HG and oxLDL (Fig. 6e). Consistently, melatonin attenuated the increase of SNO-GNAI2 in HUVECs treated with HG and oxLDL (Fig. 6f). To determine if melatonin reduces endothelial inflammation through the CXCR5-Hippo-YAP signaling pathway, we examined the interaction of CXCR5 and GNAI2 and the activity of Hippo-YAP signaling. In HUVECs stimulated with HG and oxLDL, melatonin reduced the interaction between GNAI2 and CXCR5 (Fig. 6g) and restored the levels of phospho-LATS1 and phospho-YAP (Fig. 6h). In addition, melatonin preserved cytoplasmic retention of YAP, as determined by immunoblot analysis of YAP in cytoplasmic and nuclear fractions, respectively, (Fig. 6i) and in situ immunofluorescence (Fig. 6j). As a result, the mRNA levels of adhesion molecules and chemokines significantly increased both in HAECs and HUVECs stimulated with HG and oxLDL, which was almost completely abolished by simultaneously treating cells with melatonin (Fig. 6k, Supplementary Fig. 6a). Melatonin also suppressed the protein expressions of ICAM1 and VCAM1 (Supplementary Fig. 6b). Attenuation of the inflammatory response was further evidenced by less adhesion of THP-1 cells to HAECs and HUVECs (Fig. 6l, Supplementary Fig. 6c) and reduced foam cell formation in cultured macrophages (Supplementary Fig. 7b) in the melatonin-treated group. Taken together, these data suggest that melatonin attenuates inflammatory response through suppressing SNO-GNAI2 signaling via inhibiting iNOS.

### Melatonin mitigates diabetes-accelerated atherosclerosis

**Fig. 7:**
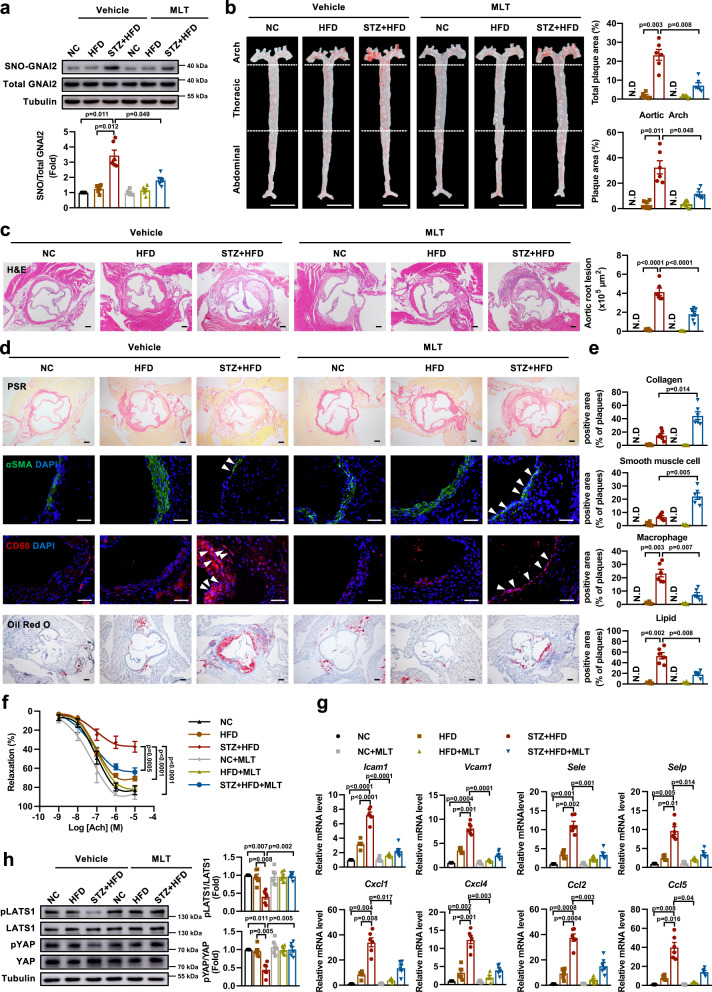
Melatonin exerts anti-inflammation and athero-protective role under diabetic condition. **a** Melatonin decreases the S-nitrosylation of GNAI2 in aortas of STZ + HFD-treated LDLr^−/−^ mice. *n* = 6 mice for each group. **b** Melatonin mitigates the diabetes-accelerated atherosclerosis as evidenced by en face Oil Red O staining of the whole aortas and melatonin mainly reduces the lesion in aortic arch. Scale bar = 5 mm. *n* = 6 mice for each group. N.D represents no detected. **c** Melatonin attenuates the plaque areas in aortic roots induced by STZ + HFD. scale bar = 200 μm. *n* = 7 mice in STZ + HFD + MLT group and *n* = 6 mice in other groups. N.D represents no detected. **d** Melatonin increases collagen deposition and smooth muscle cell content, reduces macrophage infiltration and lipid accumulation in aortas of STZ and HFD-treated LDLr^−/−^ mice, as determined by PSR staining (scale bar = 200 μm), immunofluorescent staining of α-SMA and CD68^+^ macrophage (scale bar = 50 μm), and Oil Red O staining (scale bar =200 μm). **e** Quantification of collagen content (*n* = 7 mice in STZ + HFD + MLT group and *n* = 6 mice in other groups), smooth muscle cell content (*n* = 6 mice for each group), macrophage infiltration (*n* = 6 mice for each group), and lipid accumulation (*n* = 6 mice for each group). N.D represents no detected. **f** Melatonin rescues the impairment in Ach-mediated endothelium-dependent relaxation in STZ + HFD-treated LDLr^−/−^ mice. *n* = 6 mice for each group. **g** Melatonin suppresses the mRNA levels of adhesion molecules and chemokines in STZ + HFD-treated LDLr^−/−^ mice. *n* = 6 mice for each group. **h** Melatonin restores the phosphorylation of LATS1 and YAP in the aortas of STZ + HFD-treated LDLr^−/−^ mice. *n* = 6 mice for each group. Data are represented as the Mean ± SEM. **a, b, e, g, h** Welch ANOVA followed by Tamhane’s T2 test for post-hoc comparisons. For *Icam1* in **g**, and **c, f** One-way ANOVA followed by Turkey’s test for post-hoc comparisons was used. Source data are provided as a Source Data file.

To determine the role of melatonin in diabetes-accelerated atherosclerosis, LDLr^−/−^ mice received intraperitoneal injection with STZ and fed on a HFD to develop diabetes-accelerated atherosclerosis. As expected, increased fasting blood glucose levels and loss of body weight were observed in STZ and HFD-treated LDLr^−/−^ mice compared to those fed with normal chow (Supplementary Table 5). Diabetic LDLr^−/−^ mice fed with a HFD developed high levels of total plasma cholesterol, triglyceride and low-density lipoprotein in one month, but melatonin treatment did not change the blood glucose level or lipoprotein profiles (Supplementary Table 5). In accordance with the in vitro data, melatonin decreased the expression of iNOS in the aorta of STZ and HFD-treated LDLr^−/−^ mice (Supplementary Fig. 7c). Meanwhile, the S-nitrosylation of GNAI2 was increased in the aortas of STZ and HFD-treated LDLr^−/−^ mice, which was attenuated by melatonin treatment (Fig. 7a). STZ and HFD-treated LDLr^−/−^ mice displayed abundant atherosclerotic lesions in the whole aorta, with increased plaque burden observed in aortic arch and aortic roots, and melatonin treatment reduced plaque formation at both locations (Fig. 7b, c). H&E and picric sirius red (PSR) staining of aortic roots revealed that melatonin reduced the necrotic core areas and increased fibrous cap thickness (Supplementary Fig. 7d, e). Besides, melatonin improved stability of atherosclerotic plaques, manifesting as increased collagen and smooth muscle cell content, decreased macrophage infiltration, and reduced lipid deposition (Fig. 7d, e). Melatonin also attenuated the intraplaque hemorrhage in aortic roots from STZ and HFD-treated LDLr^−/−^ mice as demonstrated by immunofluorescent staining of Ter-119 (Supplementary Fig. 7f). Besides, melatonin reduced the mRNA levels of profibrous markers (Supplementary Fig. 7g). These results demonstrate favorable athero-protective effects of melatonin independent of blood cholesterol level or glycemia.

Next, we determined the vasorelaxation of mouse aorta and found that melatonin rescued the impairment in endothelium-dependent relaxation induced by STZ and HFD (Fig. 7f). The aortic sections of STZ and HFD-treated LDLr^−/−^ mice showed greater fluorescent immunoreactivity for ICAM1 and VCAM1 in the endothelium than those of control mice. In contrast, melatonin treatment significantly reduced ICAM1 and VCAM1 immuno-positive signals (Supplementary Fig. 7h). Consistent with the immunofluorescent staining profiles, the mRNA levels of adhesion molecules and chemokines were increased in the aortas of STZ and HFD-treated LDLr^−/−^ mice but were suppressed by melatonin treatment (Fig. 7g). Furthermore, melatonin restored the phosphorylation of LATS1 and YAP in the aortas of STZ and HFD-treated LDLr^−/−^ mice (Fig. 7h). Taken together, these data indicate that melatonin alleviates diabetes-accelerated atherosclerosis through SNO-GNAI2 and Hippo-YAP signaling pathway.

## Discussion

Although NO was firstly identified as an endothelium-derived hyperpolarizing factor, it has been realized that S-nitrosylation mediated by NO exerts many physiological and pathological effects independent of cyclic guanosine monophosphate (cGMP). A large number of proteins have been found hypo- or hyper-S-nitrosylated in cardiovascular diseases, which may explain many facets of NO in the cardiovascular system. In endothelial cells, protein S-nitrosylation is involved in various physiological and pathophysiological processes, including inflammation, proliferation, apoptosis and mitochondrial energy metabolism. In fact, our previous work has shown that iNOS-induced S-nitrosylation of eNOS at Cys94 and Cys99 aggravates oxLDL-induced endothelial dysfunction^20^. In addition, S-nitrosylation of Plastin-3 exacerbates thoracic aortic dissection formation via inducing endothelial barrier dysfunction^47^. Inducible NO synthase is one of the major sources of NO in pathological condition and has been well established as a pro-inflammatory factor in various cardiovascular diseases. However, mechanisms through this protein regulates the transcription of inflammatory genes remain unclear.

In this work, it is interesting to find that the S-nitrosylation occurs in GNAI2 rather than GNAI1 or GNAI3. However, GNAI2-R67K mutant could partially but not completely inhibit the increase of GNAI2 S-nitrosylation induced by HG + oxLDL. This is also a reasonable observation, as ion pair that directs S-nitrosylation is not restricted to adjacent amino acid, it has been found that tertiary structure-derived acid-base motifs also induces protein S-nitrosylation^48,49^. Besides, hydrophobic environments, including membranes, those generated by protein structure or protein–protein interactions, can also facilitate the formation of S-nitrosylation^48^. In addition, the conformational flexibility of the secondary structure where cysteine resides in would also affects S-nitrosylation through aiding in protein–protein interactions to NO donor^50^. The amino acid sequence alignment shows a low degree of sequence identity at the region containing Cys66 (aa 61-119) among the three isoforms, indicating that there are differences in tertiary structure-derived ion charge, local hydrophobicity, and conformational flexibility among them. These differences could also cause different reactivities of GNAI2-Cys66 to SNO-donor.

Recently, Li C et al. found that activation of YAP induced inflammasome formation and increased iNOS level in macrophages^51^, our study, on the other hand, found that the iNOS-induced S-nitrosylation of GNAI2 increases endothelial inflammation through inactivating Hippo-YAP signaling, indicating a crosstalk between iNOS and Hippo-YAP signaling pathway in the context of inflammation. The Hippo-YAP pathway has been well documented in organ size control, tissue homeostasis, and cancer. The Hippo-YAP pathway also plays an essential role in inflammatory response, maintenance of vessel homeostasis, and development of the cardiovascular system^52^. Recent studies have shown that atheroprone-disturbed flow leads to YAP/TAZ activation and translocation into the nucleus, which promotes endothelial inflammation and monocyte attachment to ECs. In contrast, laminar flow decreases YAP nuclear localization and its transcriptional activity^43,44,53^. Our results were in accordance with these observations.

Although the link between YAP and inflammation has been determined, upstream signals that regulate the Hippo pathway have not been well delineated. Previous work has reported a mechanism regulating the Hippo-YAP pathway through the G12/13-coupled GPCR signaling cascades^54^. Using a RNAi-based screening strategy, we found potential roles for CXCR5 and CXCR4 in HG + oxLDL-induced endothelial inflammation. The underlying mechanism for this preference, however, requires further investigations. It should be noted that cell-type-specific preferences for GPCRs in regulating a specific pathological process (endothelial inflammation in our work) are very common. This may be integrated results of receptor levels, ligands concentrations, intracellular effector preferences and the microenvironments of cells. In our work, knockdown of CXCR5 or CXCR4 induced the strongest anti-inflammatory effects than any other receptors. Furthermore, knockdown of GPER1 even increased the level of inflammatory factors, indicating that different Gi-coupled receptors have very distinct functions. Our observations provide insight into the role of GNAI2 and CXCR5 in the Hippo-YAP pathway. We found that SNO-GNAI2 couples to CXCR5 and inactivates LATS1/2 kinase, leading to YAP dephosphorylation, nuclear localization, and inflammatory genes expression. Several recent studies provide evidence that the actin cytoskeleton modulates the Hippo pathway^55^. We also postulate that coupling of SNO-GNAI2 with CXCR5 might induce signal to LATS1/2 through rearrangement of cytoskeleton.

G-protein-coupled receptors are targets of more than 40% drugs. They mediate the extracellular signal to intercellular signaling pathways through coupling to specific G proteins. It is well established that CXCR5 and its ligand CXCL13 play important roles in inflammation and immunity, and recent studies raised roles for this receptor in growth and metastasis of tumor. It has been reported that overexpression of CXCR5 in mesenchymal stromal cells suppresses inflammatory cell infiltration and pro-inflammatory cytokines production^56^. In addition, in prostate cancer cells, CXCL13 promotes cell migration and tumorigenesis through the CXCL13-CXCR5 pathway^57^. However, the role of CXCR5 in endothelial cells and in the process of atherosclerosis is largely unknown. Our findings show that similar to what observed in T cells, CXCR5 in endothelial cells mediates inflammatory reaction in response to extracellular stimuli. Signaling pathways downstream of CXCR5 include PI3K/Akt, MEK/ERK, and Rac pathways. Here in our study, we exclude the involvement of the cAMP-MAPKs pathway and the Akt/mTOR pathways in the regulation of CXCR5-mediated endothelial inflammation, confirming that coupling of SNO-GNAI2 to CXCR5 specifically regulates Hippo-YAP signaling in condition of diabetes-accelerated atherosclerosis. Similarly, a recent work published by Sun JP and coworkers demonstrated that palmitoylation on C351 at the cytosolic tail of the G_o_ protein was essential for efficient engagement with adhesion G-protein-coupled receptor G3 (ADGRG3) but was not observed in other GPCR complex structures. They observed that the palmitoylation on G_o_ inserted deeply into the 7TM core and interacted with H362^3.53^ and L363^3.54^ of GPR97, the intracellular loop 1 (ICL1) of the receptor adopted a one-turn helical structure that formed direct contacts with Gβ^58^. We propose here a similar allosteric regulating mechanism that S-nitrosylation of GNIA2 promotes the ability of CXCR5 rather than other GPCRs to engage its active Gi protein-coupled state.

Melatonin has been found to inhibit iNOS expression in murine macrophages through inhibiting NF-κB activation, but its role in diabetes-accelerated atherosclerosis and the underlying mechanism is largely unknown. In the present study, we demonstrate that melatonin dose-dependently decreases the expression of iNOS and attenuates S-nitrosylation of GNAI2. In accordance with what has been found by H Vural et al.^59^ and Goldberg IJ et al.^60^, our present study suggests that the protective effects of melatonin are independent of correcting hyperglycemia or hyperlipidemia, potentially implying that melatonin alleviates diabetes-accelerated atherosclerosis through an intrinsic shared mechanism between diabetes and atherosclerosis. In fact, considering the fundamental role of inflammation in atherosclerosis and diabetes mellitus, it is reasonable to ascribe the protective effects of melatonin to alleviating endothelial inflammation.

**Fig. 8:**
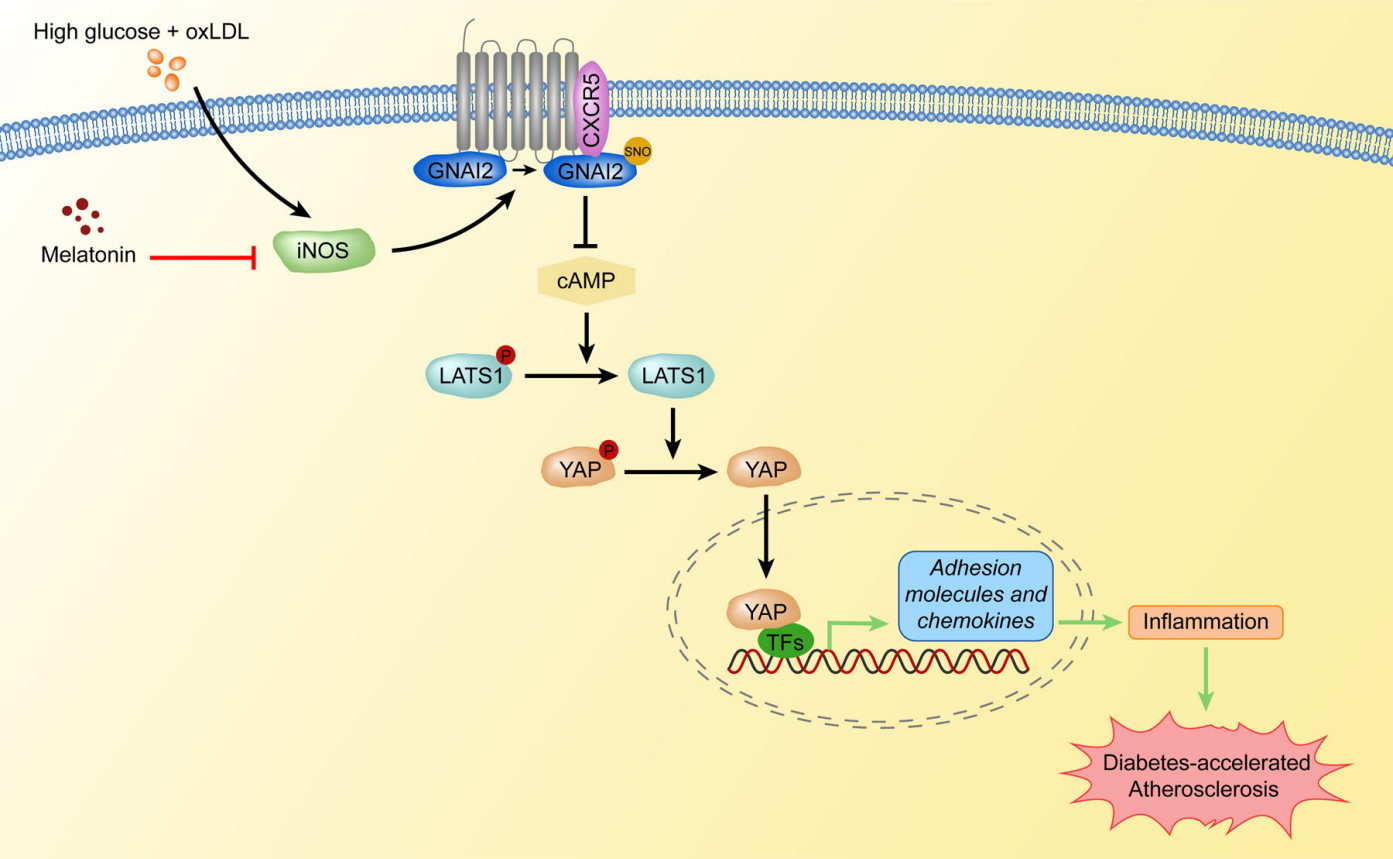
Graphic model of SNO-GNAI2-induced diabetes-accelerated atherosclerosis. During diabetes-accelerated atherosclerosis, high glucose, and oxLDL increases S-nitrosylation of GNAI2 in endothelial cells, which enhances coupling with CXCR5 and reduces cAMP level. The reduction in cAMP dephosphorylates LATS1 and YAP, promotes nuclear translocation of YAP and promotes transcription of adhesion molecules and chemokines, which enhances endothelial inflammation. Melatonin restores phosphorylation of LAST1 and YAP through reducing iNOS-induced SNO-GNAI2 and alleviates endothelial inflammation, which improves diabetes-accelerated atherosclerosis. oxLDL oxidized low-density lipoprotein, GNAI2 guanine nucleotide-binding protein G(i) subunit alpha-2, CXCR5 C-X-C chemokine receptor type 5, cAMP cyclic adenosine monophosphate, LATS1 large tumor suppressor kinase 1, YAP Yes-associated protein, iNOS inducible nitric oxide synthase.

In conclusion, we found S-nitrosylation of GNAI2 at Cys66-mediated diabetes-accelerated atherosclerosis. Notably, we found that SNO-GNAI2 inactivated Hippo-YAP signaling through coupling to CXCR5, which contributed to endothelial inflammation during diabetes-accelerated atherosclerosis. Based on these findings, we confirmed the therapeutic effects of melatonin in diabetes-accelerated atherosclerosis, at least in part, through reducing SNO-GNAI2-induced deactivation of Hippo-YAP axis (Fig. 8). These data indicate that SNO-GNAI2 may be a potential therapeutic target against diabetes-accelerated atherosclerosis.

## Methods

### Experimental animals

Male LDLr^−/−^ mice were obtained from the Changzhou Cavens Laboratory Animal Co. Ltd. and kept at a constant temperature (22 ± 1 °C) and 50% relative humidity under a 12 h/12 h light/dark cycle with free access to food and water. Mice were rendered diabetes by intraperitoneal injections of streptozotocin (STZ, 60 mg/kg/day) or citrate buffer (nondiabetic) on 5 consecutive days. Nondiabetic mice were fed whether on a chow diet (Negative Controls, NC) or a high fed diet (HFD) for 4 weeks. STZ-treated mice with fasting blood glucose levels greater than 16.7 mmol/L were kept on HFD for 4 weeks (STZ + HFD). Mice were randomized and concurrently intraperitoneally administered with melatonin (20 mg/kg/d) (NC + MLT, HFD + MLT, STZ + HFD + MLT) or vehicle (1% ethanol) (NC, HFD, STZ + HFD). Body weight and fasting blood glucose were measured before sacrifice.

For overexpression of GNAI2 in the endothelium, 4-week-old LDLr^−/−^ mice were transduced with AAV^endo^-GFP, AAV^endo^-GNAI2-WT, and AAV^endo^-GNAI2-C66A (5 × 10^11^ GC/mouse) through tail vein injection. The recombinant adeno-associated virus (AAV) was generated by Jimai Gene Biotechnology Co. Ltd. (Suzhou, China). Four weeks after injection, diabetes-accelerated atherosclerosis was induced in mice as described above. All animal experiments were conducted according to the ARRIVE guidelines for the care and use of laboratoty animals, and protocols were approved by the Committee of Nanjing Medical University (IACUC-1811027).

### Human specimens

Human coronary artery samples were obtained from coronary artery disease (CAD) patients combined with or without diabetes subjected to coronary artery bypass grafting. Written informed consent was obtained from all patients. All procedures involving sampling were performed according to the principles outlined in the Declaration of Helsinki and were approved by the Ethics Committee of the Affiliated Drum Tower Hospital of Nanjing University Medical School (2019-219-01).

### Cell culture and treatment

Human umbilical vein endothelial cells (HUVECs) were isolated from human umbilical cords obtained from the Sir Run Run Hospital. Human aortic endothelial cells (HAECs) were a gift from Prof. Yong Xu (Nanjing Medical University). Mouse aortic endothelial cells (MAECs) were isolated from 6-week-old male CXCR5 knockout mice (B6.129S2[Cg]-Cxcr5^tm1Lipp/J^)^61^, a gift from Nantong University, which were housed at a constant temperature (22 ± 1 °C) and 50% relative humidity under a 12 h/12 h light/dark cycle with unrestricted access to food and water. HEK293T cells were a gift from Prof. Jin-Peng Sun (Shandong University). HUVECs, HAECs and MAECs were cultured in Endothelial Cell Medium (ECM, ScienCell, CA, USA) supplemented with 5% FBS and endothelial cell growth supplement (ECGS) at 37 in an incubator with 5% CO2. HEK 293T cells were maintained in DMEM (Gibco, MA, USA) supplemented with 10% (v/v) FBS (Gibco, MA, USA). Cells within seven passages were used for the in vitro studies, and cells with 80–90% confluence were used. Before treatment, endothelial cells were exposed to serum starvation for 4 h followed by treatment with either mannitol (25 mmol/L) alone, native LDL (nLDL, 50 μg/mL) alone, mannitol and native LDL (Mannitol+nLDL) or high glucose (HG, 25 mmol/L) alone, oxLDL (50 μg/mL) alone and high glucose plus oxLDL (HG + oxLDL). Simultaneously, cells were treated with or without melatonin (10 μmol/L) for 24 h.

### Measurements of serum melatonin levels

Blood samples from diabetes-accelerated atherosclerosis mice and controls were collected before sacrifice. Serum melatonin levels were determined with an enzyme-immunoassay kit (YBio, Shanghai, China) according to the manufacturer’s guide. Briefly, the samples and standards were added to the appropriate microtiter plate wells precoated with an antibody specific to melatonin. Then Avidin conjugated to HRP was added and incubated. Next, the detection reagent was added to each well and the concentration of melatonin was measured by comparing the O.D. of the samples to the standard curve.

### Plasmid and siRNA transfection

Plasmids containing HA-GNAI2-WT and HA-GNAI2-C66A were purchased from Sino Bio (Beijing, China). Small silencing RNAs (siNC, siCXCR5, siLATS1, and siYAP) were purchased from GenePharma (Shanghai, China). Plasmids or siRNAs were transfected into 75% confluent HUVECs with Lipofectamine 3000 reagent (Invitrogen). After 4–6 h of transfection, medium was changed to fresh ECM, and cells were maintained for an additional 24 h before use in experiments.

### Biotin-switch assay

A biotin-switch assay was performed to detect the S-nitrosylation status of the whole-cell or specific protein according to the manufacturer’s instructions (Cayman Chemical, Ann Arbor, USA). Briefly, the cells were dissolved with a blocking reagent, so that protein-free thiols were blocked. After centrifugation for 10 min, the supernatants were transferred into 4 volumes of ice-cold acetone followed by incubation at −20 °C for 1 h. Nitrosothiol groups were then reduced by ascorbic acid to yield free thiols, which were then covalently labeled with maleimide-biotin (Buffer B containing Reducing and Labeling Reagents, 8:1:1). Biotinylated proteins were subsequently purified by incubation with avidin-coupled agarose beads overnight at 4 °C, and the level of S-nitrosylation was analyzed by western blotting with a specific antibody. Samples not reduced by ascorbic acid were used as the negative control (-Vc).

### LC-MS/MS analysis

Liquid chromatography-tandem mass spectrometry (LC-MS/MS) analysis was performed by the Analysis Center, Nanjing Medical University. HUVECs treated with Mannitol + nLDL or HG + oxLDL were blocked with N-ethylmaleimide and labeled with biotin-maleimide. Then the biotinylated proteins were digested by trypsin (trypsin: protein = 1:50) for 16 h and purified with streptavidin-agarose for LC-MS/MS analysis^62^. LC-MS/MS analysis of SNO-peptides was carried out on a NanoLC system equipped with a TripleTOF® 5600+ (AB Sciex) mass spectrometer. Tandem mass spectrometry spectra were extracted using MS/MS spectra provided sequence-specific fragment ions (y-, b-ions) which were searched against SequestHT based on Uniprot human proteome database and variable modification of 451.54 Da (the molecular weight of Biotin-maleimide) for cysteines when the biotin-maleimide reagent was used.

### Bioluminescence resonance energy transfer (BRET) measurement

Gi BRET probes, including GNAI2-RLuc8, Gβ3, and Gγ8-GFP2, were generated according to previous report^63^. Briefly, the GNAI2-RLuc8-WT subunit was generated by amplifying and inserting the coding sequence of RLuc8 into pcDNA-GNAI2 backbone construct between residue L91 and Q92. Gγ8-GFP2 was generated by fusing the GFP2 coding sequence in frame at the N-terminus to Gγ8. GNAI2-RLuc8-C66A mutation was generated by site-directed mutagenesis. All of the constructs and mutations were verified by DNA sequencing.

For detecting the Gi protein activation, HEK293T cells were transiently co-transfected with CXCR5 or CXCR4 together with Gi BRET probes. Twenty-four hours after transfection, cells were distributed into a 96-well microplate and incubated for additional 24 h. The cells were preincubated with or without DETA for 16 h before they were stimulated with CXCL13 or CXCL12 at varying concentrations for 5 min. BRET signal was measured after the addition of the luciferase substrate coelenterazine 400a using a Mithras LB940 microplate reader (Berthold Technologies). The BRET signal was calculated as the ratio of light emission of GFP2 (510 nm) to RLuc8 (400 nm).

### Measurement of cAMP level

Cells were incubated in 12-well plates, after different treatments, cells were incubated with 3-isobutyl-1-methylxanthine (IBMX, 0.5 mmol/L) for 20 min, followed by stimulation with forskolin (FSK, 10 μmol/L) for 15 min. The reactions were stopped by ice-cold perchloric acid containing 4 mM theophylline and neutralized with ice-cold KHCO3. The extracts were assayed for cAMP concentration by cAMP specific enzyme-immunoassay kit (Cayman, Chemical) according to the manufacturer’s instruments.

### En face analysis of atherosclerosis and plaque histology

After sacrifice, mouse aortas were collected from the base ascending aorta to the iliac bifurcation. Atherosclerosis was determined by Oil Red O staining. Hematoxylin and eosin (H&E) staining was used to show basic lesion morphology and necrotic core. For quantitative analysis of lipids and collagen deposition, 10 consecutive sections from the aortic roots of each mouse were stained with Oil Red O, Picrosirius Red (PSR). The fibrous cap thickness was measured by averaging the thickness in three separate regions of the lesions and expressed as the cap thickness/plaque area ratio. Plaque areas, lipid content and collagen content and cap thickness were quantified with Image-Pro Plus 6.0 software.

### Monocyte adhesion assay

THP-1 monocytes were purchased from National Infrastructure of Cell Line Resource (Shanghai, China). THP-1 cells were cultured in RPMI 1640 medium supplemented with 10% FBS in a humidified atmosphere with 5% CO_2_. THP-1 cells were labeled with CM-Dil (2.5 μg/mL) at 37 °C for 10 min. Labeled cells were then incubated with HUVECs or HAECs with different treatments for 60 min at 37 °C. After incubation, nonadherent THP-1 cells were gently washed away with PBS. Fluorescence images of adherent THP-1 cells were captured and analyzed using ImageJ software to estimate the number of monocytes adhered to endothelial cells.

### Immunofluorescent staining

HUVECs, aortic root sections, and aorta sections were fixed with 4% paraformaldehyde for 15 min, permeabilized with 0.2% Triton X-100 for 20 min, and blocked with 10% fetal bovine serum for 1 h at room temperature. Samples were then incubated with primary antibodies at 4 °C overnight followed by corresponding secondary antibody incubation. Images were captured using ZEISS LSM 800 confocal microscope (Germany). The antibodies used are listed in Supplementary Table 1.

### Extraction of cytosolic and nuclear fraction

HUVECs were washed with PBS and scraped with lysis buffer A (10 mM HEPES, 0.1 mM EDTA, 1 mM KCl, 50 mM NaF, 0.1 mM EGTA, 1 mM Na_3_VO_4_, 1 mM DTT, and protease inhibitor cocktail). Cells were then oscillated 24 times on a vortex, and homogenates were centrifuged at 9391 × *g* for 5 min at 4 °C. The supernatant was collected as the cytosolic fraction. The pellet was further lysed with buffer C (20 mM HEPES, 1 mM EDTA, 0.4 M NaCl, 50 mM NaF, 1 mM EGTA, 25% Glycerol, 1 mM DTT and protease inhibitor cocktail) for 1 h on ice and centrifuged at 9391 × *g* for 10 min at 4 °C. The obtained supernatant was considered as the nuclear fraction.

### Coimmunoprecipitation

HUVECs were harvested after treatment and lysed in buffer containing 40 mM HEPES (pH = 7.4), 2 mM EDTA, 0.5% Triton X-100, phosphatase inhibitors and protease inhibitors. Cells were placed on a low-speed rotating shaker for 30 min at 4 °C and then centrifuged at 12,000 × *g* for 10 min. Whole-cell lysates (50 μL) were loaded as input sample, and the remaining cell lysates were incubated with the indicated antibody at 4 °C overnight, the immune complex was then captured on protein A/G agarose beads. Beads were washed five times with washing buffer and boiled. All samples were subjected to SDS-PAGE separation and western blotting with the indicated antibodies (listed in Supplementary Table 2).

### Western blotting

Endothelial cells or mouse aorta were lysed on ice with RIPA lysis buffer supplemented with a protease and phosphatase inhibitor cocktail. Lysates were centrifuged at 13,523 × *g* for 15 min at 4 °C, and protein concentration was determined using the Bradford Assay. Proteins were separated by SDS-PAGE and transferred to PVDF membranes (Millipore). Target proteins were incubated with the appropriate primary antibodies overnight at 4 °C and the corresponding secondary antibodies for 2 h at room temperature followed by chemiluminescent detection (Bio-Rad) with American Image 600 (AI600, GE, USA) and analyzed by Image J software (v1.53, National Institutes of Health, USA). The primary antibodies used in this study are listed in Supplementary Table 2 and the full scan blots are provided in the Source Data file.

### Quantitative real-time PCR analysis

Total RNA was extracted using RNAiso Plus (Takara, 9109) according to the manufacturer’s instructions. cDNA was synthesized using HiScript II Q RT SuperMix for qPCR Kit (Vazyme). Quantitative PCR was performed by ChamQ^TM^ SYBR® qPCR Master Mix (Vazyme). The expression levels of target genes were normalized to GAPDH and expressed as relative mRNA levels compared to internal control. QuantStudio Design and Analysis software (v1.5.1, ABI, USA) was used for real-time PCR data collection. The primer sequences used for quantitative real-time PCR are listed in Supplementary Table 3.

### Statistical analysis

All data were represented as the Mean ± standard error of the mean (SEM). Unpaired two-tailed Student’s *t*-test was used for comparisons between two groups when data passed normality and equal variance test, otherwise Mann–Whitney test was used. Differences among groups were evaluated using one-way ANOVA followed by Tukey’s test or Dunnett’s test for post-hoc comparison when appropriate. Otherwise Welch ANOVA test followed by Tamhane’s T2 test for post-hoc comparisons was performed. *P*-values < 0.05 were considered statistically significant. Statistical analyses were performed with GraphPad Prism 8.0 software.

### Reporting summary

Further information on research design is available in the Nature Research Reporting Summary linked to this article.

## Supplementary information

Supplementary Information

Reporting Summary

## Data Availability

Raw RNA-sequence data for GPCRs expression in HUVECs were deposited at the GEO database https://www.ncbi.nlm.nih.gov/geo/query/acc.cgi?acc=GSE173669 with accession codes GSE173669. Mass Spectrometry data for protein S-nitrosylation have been deposited to the ProteomeXchange Consortium via the PRIDE partner repository with the dataset identifier PXD025295 with hyperlinks through http://proteomecentral.proteomexchange.org/cgi/GetDataset?ID=PXD025295. Raw data of all figures and uncropped versions of any gels or blots presented in the figures are provided as a Source Data file. Source data are provided with this paper.

## Source data

Source Data

## References

[1] Low Wang CC, Hess CN, Hiatt WR, Goldfine AB (2016). Clinical update: cardiovascular disease in Diabetes mellitus: atherosclerotic cardiovascular disease and heart failure in type 2 Diabetes mellitus—mechanisms, management, and clinical considerations. Circulation.

[2] Funk, S. D., Yurdagul, A., Jr. & Orr, A. W. Hyperglycemia and endothelial dysfunction in atherosclerosis: lessons from type 1 diabetes. *Int. J. Vasc. Med*. **2012**, 569654 (2012). 10.1155/2012/569654 PMC3303762 22489274

[3] Kannel WB, McGee DL (1979). Diabetes and cardiovascular disease. The Framingham study. JAMA.

[4] Renard CB (2004). Diabetes and diabetes-associated lipid abnormalities have distinct effects on initiation and progression of atherosclerotic lesions. J. Clin. Invest..

[5] Dahl-Jorgensen K, Larsen JR, Hanssen KF (2005). Atherosclerosis in childhood and adolescent type 1 diabetes: early disease, early treatment?. Diabetologia.

[6] Tancredi M (2015). Excess mortality among persons with type 2 diabetes. N. Engl. J. Med..

[7] Pavlansky R (1970). [70 years of Professor Dr. Jan Knobloch, DrSc., bearer of the Order of Work]. Acta Chir. Orthop. Traumatol. Cech..

[8] Zeadin MG, Petlura CI, Werstuck GH (2013). Molecular mechanisms linking diabetes to the accelerated development of atherosclerosis. Can. J. Diabetes.

[9] Ley K, Laudanna C, Cybulsky MI, Nourshargh S (2007). Getting to the site of inflammation: the leukocyte adhesion cascade updated. Nat. Rev. Immunol..

[10] Jones, D. P., True, H. D. & Patel, J. Leukocyte trafficking in cardiovascular disease: insights from experimental models. *Mediators Inflamm.* **2017**, 9746169 (2017). 10.1155/2017/9746169 PMC5390637 28465628

[11] Kanter JE, Johansson F, LeBoeuf RC, Bornfeldt KE (2007). Do glucose and lipids exert independent effects on atherosclerotic lesion initiation or progression to advanced plaques?. Circ. Res..

[12] Guzik TJ, Korbut R, Adamek-Guzik T (2003). Nitric oxide and superoxide in inflammation and immune regulation. J. Physiol. Pharmacol..

[13] Haldar SM, Stamler JS (2013). S-nitrosylation: integrator of cardiovascular performance and oxygen delivery. J. Clin. Invest..

[14] Lima B, Forrester MT, Hess DT, Stamler JS (2010). S-nitrosylation in cardiovascular signaling. Circ. Res..

[15] Ravi K, Brennan LA, Levic S, Ross PA, Black SM (2004). S-nitrosylation of endothelial nitric oxide synthase is associated with monomerization and decreased enzyme activity. Proc. Natl Acad. Sci. USA.

[16] Hess DT, Matsumoto A, Kim SO, Marshall HE, Stamler JS (2005). Protein S-nitrosylation: purview and parameters. Nat. Rev. Mol. Cell Biol..

[17] Murphy E (2014). Signaling by S-nitrosylation in the heart. J. Mol. Cell Cardiol..

[18] Schiattarella GG (2019). Nitrosative stress drives heart failure with preserved ejection fraction. Nature.

[19] Li J (2018). GSNOR modulates hyperhomocysteinemia-induced T cell activation and atherosclerosis by switching Akt S-nitrosylation to phosphorylation. Redox Biol..

[20] Wang, W. et al. eNOS S-nitrosylation mediated OxLDL-induced endothelial dysfunction via increasing the interaction of eNOS with betacatenin. *Biochim. Biophys. Acta Mol. Basis Dis.* **1865**, 1793-1801 (2019). 10.1016/j.bbadis.2018.02.009 29471036

[21] Wilkie TM (1992). Evolution of the mammalian G protein alpha subunit multigene family. Nat. Genet.

[22] Huang X (2008). Resistance to diet-induced obesity and improved insulin sensitivity in mice with a regulator of G protein signaling-insensitive G184S Gnai2 allele. Diabetes.

[23] Moxham CM, Malbon CC (1996). Insulin action impaired by deficiency of the G-protein subunit G ialpha2. Nature.

[24] Whalen EJ (2007). Regulation of beta-adrenergic receptor signaling by S-nitrosylation of G-protein-coupled receptor kinase 2. Cell.

[25] Hayashi H (2018). S-nitrosylation of beta-arrestins biases receptor signaling and confers ligand independence. Mol. Cell.

[26] Stehle JH (2011). A survey of molecular details in the human pineal gland in the light of phylogeny, structure, function and chronobiological diseases. J. Pineal Res.

[27] Lax P (2011). Circadian dysfunction in P23H rhodopsin transgenic rats: effects of exogenous melatonin. J. Pineal Res..

[28] Gandhi AV, Mosser EA, Oikonomou G, Prober DA (2015). Melatonin is required for the circadian regulation of sleep. Neuron.

[29] Calvo JR, Gonzalez-Yanes C, Maldonado MD (2013). The role of melatonin in the cells of the innate immunity: a review. J. Pineal Res.

[30] Gao, Y. et al. Melatonin synergizes the chemotherapeutic effect of 5-fluorouracil in colon cancer by suppressing PI3K/AKT and NF-kappaB/iNOS signaling pathways. *J. Pineal Res.* **62**, 12380 (2017). 10.1111/jpi.12380 27865009

[31] Dominguez-Rodriguez A (2004). Light/dark patterns of interleukin-6 in relation to the pineal hormone melatonin in patients with acute myocardial infarction. Cytokine.

[32] de Juan A (2019). Artery-associated sympathetic innervation drives rhythmic vascular inflammation of arteries and veins. Circulation.

[33] Viswambharan H (2007). Mutation of the circadian clock gene Per2 alters vascular endothelial function. Circulation.

[34] Yu, L. et al. Melatonin rescues cardiac thioredoxin system during ischemia-reperfusion injury in acute hyperglycemic state by restoring Notch1/Hes1/Akt signaling in a membrane receptor-dependent manner. *J Pineal Res.* **62**, 12375 (2017). 10.1111/jpi.12375 27753144

[35] Stamler JS, Toone EJ, Lipton SA, Sucher NJ (1997). (S)NO signals: translocation, regulation, and a consensus motif. Neuron.

[36] Ascenzi P (2000). Re-evaluation of amino acid sequence and structural consensus rules for cysteine-nitric oxide reactivity. Biol. Chem..

[37] Marino SM, Gladyshev VN (2010). Structural analysis of cysteine S-nitrosylation: a modified acid-based motif and the emerging role of trans-nitrosylation. J. Mol. Biol..

[38] Deng JN (2019). Cardiotonic pills plus recombinant human prourokinase ameliorates atherosclerotic lesions in LDLR(-/-) Mice. Front. Physiol..

[39] Pierce KL, Premont RT, Lefkowitz RJ (2002). Seven-transmembrane receptors. Nat. Rev. Mol. Cell Biol..

[40] Denton AE (2019). Type I interferon induces CXCL13 to support ectopic germinal center formation. J. Exp. Med..

[41] Rodriguez-Frade JM, Martinez AC, Mellado M (2005). Chemokine signaling defines novel targets for therapeutic intervention. Mini Rev. Med. Chem..

[42] Franco R, Martinez-Pinilla E, Navarro G, Zamarbide M (2017). Potential of GPCRs to modulate MAPK and mTOR pathways in Alzheimer’s disease. Prog. Neurobiol..

[43] Wang KC (2016). Flow-dependent YAP/TAZ activities regulate endothelial phenotypes and atherosclerosis. Proc. Natl Acad. Sci. USA.

[44] Wang L (2016). Integrin-YAP/TAZ-JNK cascade mediates atheroprotective effect of unidirectional shear flow. Nature.

[45] Zhao B (2007). Inactivation of YAP oncoprotein by the Hippo pathway is involved in cell contact inhibition and tissue growth control. Genes Dev..

[46] Zhang, Y. et al. Melatonin prevents endothelial cell pyroptosis via regulation of long noncoding RNA MEG3/miR-223/NLRP3 axis. *J. Pineal Res.* **64**, 12449 (2018). 10.1111/jpi.12449 29024030

[47] Pan L (2020). S-nitrosylation of plastin-3 exacerbates thoracic aortic dissection formation via endothelial barrier dysfunction. Arterioscler Thromb. Vasc. Biol..

[48] Hess DT, Matsumoto A, Nudelman R, Stamler JS (2001). S-nitrosylation: spectrum and specificity. Nat. Cell Biol..

[49] Savidge TC (2011). Host S-nitrosylation inhibits clostridial small molecule-activated glucosylating toxins. Nat. Med..

[50] Doulias PT (2010). Structural profiling of endogenous S-nitrosocysteine residues reveals unique features that accommodate diverse mechanisms for protein S-nitrosylation. Proc. Natl Acad. Sci. USA.

[51] Li C (2019). Hippo signaling controls NLR family pyrin domain containing 3 activation and governs immunoregulation of mesenchymal stem cells in mouse liver injury. Hepatology.

[52] Zhou Q, Li L, Zhao B, Guan KL (2015). The hippo pathway in heart development, regeneration, and diseases. Circ. Res.

[53] Xu S, Koroleva M, Yin M, Jin ZG (2016). Atheroprotective laminar flow inhibits Hippo pathway effector YAP in endothelial cells. Transl. Res..

[54] Yu FX (2012). Regulation of the Hippo-YAP pathway by G-protein-coupled receptor signaling. Cell.

[55] Xu S (2019). The novel coronary artery disease risk gene JCAD/KIAA1462 promotes endothelial dysfunction and atherosclerosis. Eur. Heart J..

[56] Zhang X (2017). CXCR5-overexpressing mesenchymal stromal cells exhibit enhanced homing and can decrease contact hypersensitivity. Mol. Ther..

[57] Garg R (2017). Protein kinase C epsilon cooperates with PTEN loss for prostate tumorigenesis through the CXCL13-CXCR5 pathway. Cell Rep..

[58] Ping YQ (2021). Structures of the glucocorticoid-bound adhesion receptor GPR97-Go complex. Nature.

[59] Vural H, Sabuncu T, Arslan SO, Aksoy N (2001). Melatonin inhibits lipid peroxidation and stimulates the antioxidant status of diabetic rats. J. Pineal Res..

[60] Goldberg IJ, Dansky HM (2006). Diabetic vascular disease: an experimental objective. Arterioscler Thromb. Vasc. Biol..

[61] Jiang BC (2016). CXCL13 drives spinal astrocyte activation and neuropathic pain via CXCR5. J. Clin. Invest.

[62] Tang X (2020). SNO-MLP (S-nitrosylation of muscle LIM protein) facilitates myocardial hypertrophy through TLR3 (Toll-like receptor 3)-mediated RIP3 (receptor-interacting protein kinase 3) and NLRP3 (NOD-like receptor pyrin domain containing 3) inflammasome activation. Circulation.

[63] Olsen RHJ (2020). TRUPATH, an open-source biosensor platform for interrogating the GPCR transducerome. Nat. Chem. Biol..

