# Supplementary material for: S-nitrosylation-mediated coupling of G-protein alpha-2 with CXCR5 induces Hippo/YAP-dependent diabetes-accelerated atherosclerosis
Source: Nat Commun. 2021 Jul 22;12:4452. doi: 10.1038/s41467-021-24736-y (PMC8298471; doi:10.1038/s41467-021-24736-y)
Supplement: Supplementary file 1 — Supplementary Information [file 41467_2021_24736_MOESM1_ESM.pdf]

S-nitrosylation-mediated coupling of G-protein alpha-2 with CXCR5 induces Hippo/YAP-dependent diabetes-accelerated atherosclerosis

Supplementary information

Supplementary Table 1. Antibodies for Immunofluorescence staining.

Antibody	concentration	Sources of species	Cat No.	Manufacturer
anti-CD68	1:100	Rabbit	GB11067	Servicebio
anti-ICAM1	1:100	Mouse	SC8439	Santa Cruz
anti-VCAM1	1:50	Mouse	SC13160	Santa Cruz
anti-GNAI2	1:100	Rabbit	11136-1-AP	Proteintech
anti-CXCR5	1:100	Mouse	SC373775	Santa Cruz
anti-YAP	1:100	Rabbit	14074	CST
anti-CD31	1:100	Goat	AF3628	R&D system
anti-Ter119	1:100	Mouse	13-5921-82	Invitrogen
anti-αSMA	1:100	Rabbit	ab124964	Abcam

Supplementary Table 2. Antibodies for immunoblot analysis.

Antibody	Cat No.	Manufacturer	Sources of species	MW (kDa)
ICAM1	SC8439	Santa Cruz	Mouse	100
VCAM1	SC13160	Santa Cruz	Rabbit	110
iNOS	ab178945	Abcam	Rabbit	131
p-eNOS	9571	CST	Rabbit	140
eNOS	610297	BD	Mouse	140
Trx	ab86255	Abcam	Rabbit	12
GSNOR	ab177932	Abcam	Rabbit	40
GNAI2	11136-1-AP	Proteintech	Rabbit	38
CXCR5	ab133706	Abcam	Rabbit	42

pLATS1	9157	CST	Rabbit	140
LATS1	DF7517	Affitiny	Rabbit	140
pYAP	13008	CST	Rabbit	75
YAP	14074	CST	Rabbit	75
pAKT	9271	CST	Rabbit	60
AKT	9272	CST	Rabbit	60
p-mTOR	2974	CST	Rabbit	289
mTOR	2983	CST	Rabbit	289
pERK1/2	9101	CST	Rabbit	42,44
ERK1/2	9102	CST	Rabbit	42,44
Tubulin	BS1482M	Bioworld	Mouse	50
β-actin	AP0060	Bioworld	Rabbit	42
GAPDH	AP0063	Bioworld	Rabbit	36
H3	9715	CST	Rabbit	17

Supplementary Table 3. The primer sequences used in this study.

Gene	Primer sequence
Icam1 (mouse)	Forward: GTGATGCTCAGGTATCCATCCA
	Reverse: CACAGTTCTCAAAGCACAGCG
ICAM1 (human)	Forward: TGACCGTGAATGTGCTCTCC
	Reverse: TCCCTTTTTGGGCCTGTTGT
Vcam1 (mouse)	Forward: AGTTGGGGATTCGGTTGTTCT
	Reverse: CCCCTCATTCCTTACCACCC
VCAM1(human)	Forward: AATGCCTGGGAAGATGGTCG
	Reverse: GATGTGGTCCCCTCATTCGT
Sele (mouse)	Forward: ATGCCTCGCGCTTTCTCTC
	Reverse: GTAGTCCCGCTGACAGTATGC
SELE (human)	Forward: GCCTGCAATGTGGTTGAGTG
	Reverse: ACGAACCCATTGGCTGGATT
Selp (mouse)	Forward: CATCTGGTTCAGTGCTTTGATCT
	Reverse: ACCCGTGAGTTATTCCATGAGT

SELP (human)	Forward: ACCTTCAGGACAATGGACAGCAG	
	Reverse: CCCAGAGGTTGGAGCAGTTCA	
Cxcl1 (mouse)	Forward: CTGGGATTCACCTCAAGAACATC	
	Reverse: CAGGGTCAAGGCAAGCCTC	
Cxcl4 (mouse)	Forward: AGTCCTGAGCTGCTGCTTCT	
	Reverse: GATCTCCATCGCTTTCTTCG	
CXCL4 (human)	Forward: TGAAGAATGGAAGGAAAATTTGC	
	Reverse: CAAATGCACACACGTAGGCAGCT	
CXCL8 (human)	Forward: ACTGAGAGTGATTGAGAGTGGAC	
	Reverse: AACCCTCTGCACCCAGTTTTC	
Ccl2 (mouse)	Forward: TTAAAAACCTGGATCGGAACCAA	
	Reverse: GCATTAGCTTCAGATTTACGGGT	
CCL2 (human)	Forward: CAGCCAGATGCAATCAATGCC	
	Reverse: TGGAATCCTGAACCCACTTCT	
Ccl5 (mouse)	Forward: GCTGCTTTGCCTACCTCTCC	
	Reverse: TCGAGTGACAAACACGACTGC	
CCL5 (human)	Forward: CCAGCAGTCGTCTTTGTCAC	
	Reverse: CTCTGGGTTGGCACACACTT	
iNOS (human)	Forward: TCCCGAAGTTCTCAAGGCAC	
	Reverse: GTGTCACTGGACTGGAGGTG	
Gapdh (mouse)	Forward: AGGTCGGTGTGAACGGATTTG	
	Reverse: TGTAGACCATGTAGTTGAGGTCA	
GAPDH (human)	Forward: GGAGCGAGATCCCTCCAAAAT	
	Reverse: GGCTGTTGTCATACTTCTCATGG	
Col4a (mouse)	Forward: CTGGCACAAAAGGGACGAG	
	Reverse: ACGTGGCCGAGAATTTCACC	
Fibronectin (mouse)	Forward: ATGTGGACCCCTCCTGATAGT	
	Reverse: GCCCAGTGATTTCAGCAAAGG	
Ctgf (mouse)	Forward: CTAAGACCTGTGGAATGGGC	
	Reverse: CTCAAAGATGTCATTGTCCCC	

Supplementary Table 4. Body weight, fasting blood glucose and plasma lipid profiles in LDLr$^{-/-}$ mice transduced with AAV^{endo}-GFP, AAV^{endo}-GNAI2-WT and AAV^{endo}-GNAI2-C66A respectively, and followed by NC, HFD or STZ+HFD treatment for 4 weeks.

	AAV^{endo}-GFP			AAV^{endo}-GNAI2-WT			AAV^{endo}-GNAI2-C66A		
	NC	HFD	STZ+HFD	NC	HFD	STZ+HFD	NC	HFD	STZ+HFD
Body weight (g)	27.4 ± 0.48	28.3 ± 0.47	16.5 ± 0.66*	27.5 ± 0.43	28.1 ± 0.52	16.3 ± 1.05#	28.1 ± 0.38	28.1 ± 0.35	14.6 ± 0.89$
Blood glucose (mmol/L)	7.7 ± 0.37	7.4 ± 0.34	22.4 ± 1.44*	8.2 ± 0.68	8.8 ± 0.43	24.1 ± 1.42#	8.7 ± 0.57	8.5 ± 0.48	25.5± 1.80$
TC (mmol/L)	10.4 ± 0.73	47.0 ± 1.11	157.8 ± 5.42*	12.4 ± 1.44	43.9 ± 1.38	127.1 ± 12.96#	10.6 ± 0.96	38.9 ± 1.53	138.5 ± 7.00$
TG (mmol/L)	0.9 ± 0.14	9.2 ± 0.87	21.7 ± 2.18*	1.3 ± 0.26	6.7 ± 0.78	24.8 ± 3.00#	1.2 ± 0.30	4.2 ± 0.42	23.0 ± 2.00$
LDL-C (mmol/L)	1.7 ± 0.34	11.4 ± 1.12	43.3 ± 2.13*	1.6 ± 0.35	11.1 ± 1.07	33.1 ± 2.95#	1.6 ± 0.29	9.3 ± 0.77	38.2 ± 3.90$

All results are expressed as mean ± SEM. TC, total cholesterol; TG, Triglyceride; LDL-C, Low-density lipoprotein cholesterol. Body weight and blood glucose: n=5 mice in AAV^{endo}-GFP NC, AAV^{endo}-GFP HFD, AAV^{endo}-WT STZ+HFD and AAV^{endo}-C66A STZ+HFD groups; n=6 mice in AAV^{endo}-GFP STZ+HFD, AAV^{endo}-WT NC, AAV^{endo}-WT HFD, AAV^{endo}-C66A NC and AAV^{endo}-C66A HFD groups. TC, TG and LDL-C levels: n= 5 mice for each group. *P<0.05 vs. AAV^{endo}-GFP NC; #P<0.05 vs. AAV^{endo}-GNAI2-WT NC; $<0.05 vs. AAV^{endo}-GNAI2-C66A NC. For body weight and TG, one-way ANOVA followed by Turkey's test for post hoc comparisons was used. For blood glucose, TC and LDL-C, Welch ANOVA followed

by Tamhane's T2 test for post hoc comparisons was used. Source data are provided as a Source Data file.

Supplementary Table 5. Body weight, fasting blood glucose and plasma lipid profiles in LDLr$^{-/-}$ mice treated with NC, HFD or STZ+HFD, and with or without melatonin.

	NC	HFD	STZ+HFD	NC+MLT	HFD+MLT	STZ+HFD+MLT
Body weight (g)	25.7 ± 0.78	25.7 ± 0.61	18.3 ± 1.79*	25.1 ± 1.09	24.9 ± 0.63	18.5 ± 0.78#
Blood glucose (mmol/L)	8.5 ± 0.80	8.2 ± 0.40	26.4 ± 1.66*	8.6 ± 0.69	9.1 ± 0.41	23.4 ± 2.19#
TC (mmol/L)	9.2 ± 1.24	36.1 ± 2.33	138.9 ± 12.46*	9.8 ± 1.19	33.8 ± 4.49	129.2 ± 9.81#
TG (mmol/L)	1.1 ± 0.21	9.0 ± 0.80	22.7 ± 3.21*	0.9 ± 0.17	8.8 ± 1.18	21.5 ± 2.50#
LDL-C (mmol/L)	1.1 ± 0.21	9.7 ± 0.83	36.1 ± 5.43*	1.0 ± 0.19	10.0 ± 1.03	36.6 ± 5.12#

All results are expressed as mean ± SEM. TC, total cholesterol; TG, Triglyceride; LDL-C, Low-density lipoprotein cholesterol. Body weight and blood glucose: n=5 mice in NC and NC+MLT groups; n=8 mice in HFD and HFD+MLT groups; n=10 mice in STZ+HFD group; n=9 mice in STZ+HFD+MLT group. TC, TG and LDL-C levels: n= 5 mice for each group. *P<0.05 vs. NC; #P<0.05 vs. NC+MLT. For body weight, one-way ANOVA followed by Turkey's test for post hoc comparisons was used. For blood glucose, TC, TG and LDL-C, Welch ANOVA followed by Tamhane's T2 test for post hoc comparisons was used. Source data are provided as a Source Data file.

Supplementary Figure 1

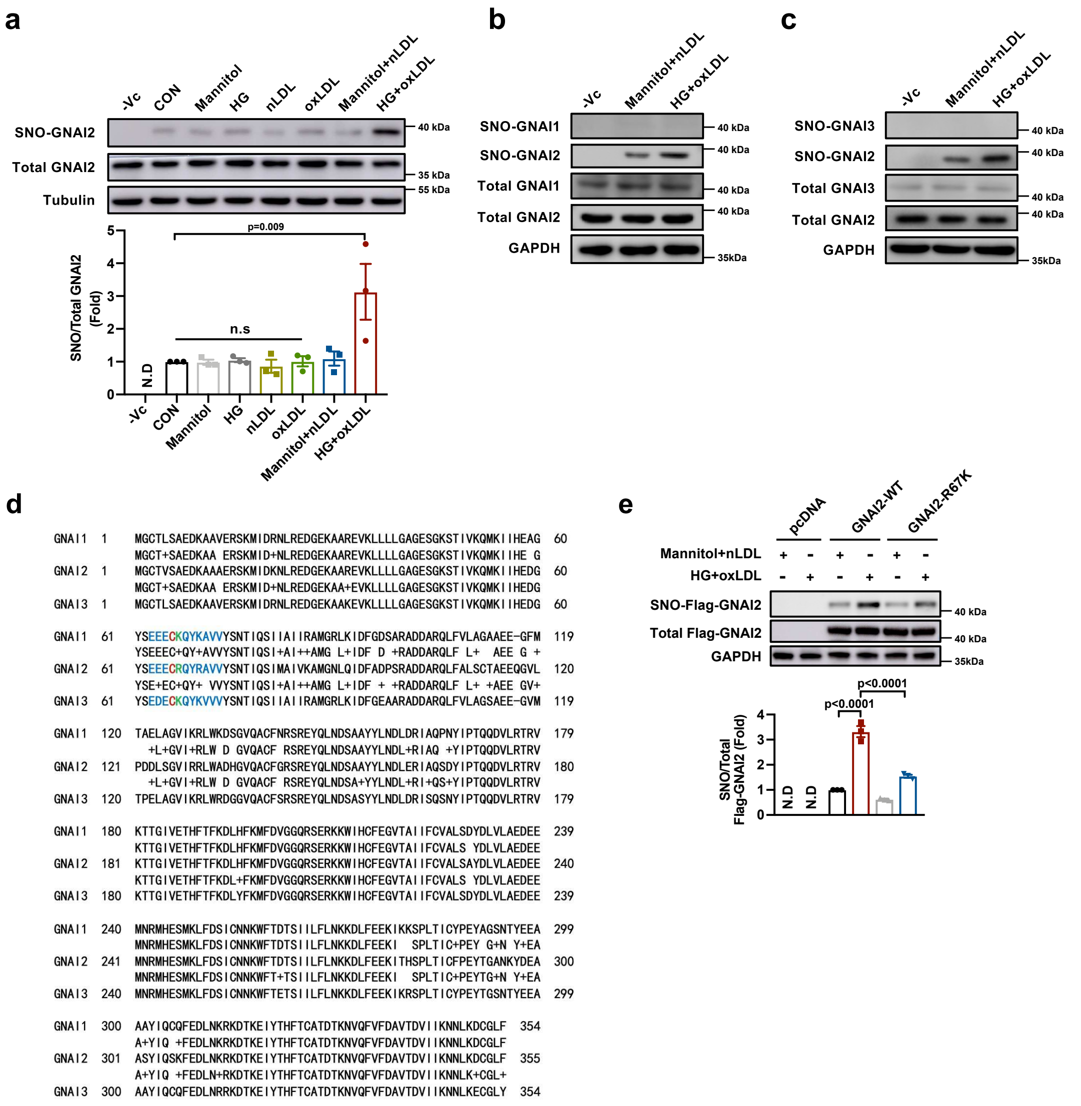

Supplementary Figure 1: S-nitrosylation of GNAI1, 2 and 3. (a) S-nitrosylation of GNAI2 is not increased in HUVECs treated with mannitol alone, nLDL alone, HG alone or oxLDL alone. n=3 distinct samples for each group. N.D represents no detected. n.s represents no significance. (b, c) S-nitrosylation of GNAI1 and GNAI3 are not detected in HUVECs treated with Mannitol+nLDL or HG+oxLDL for 24 h. n=3 distinct samples for each group. (d) Amino acid sequence alignment of GNAI1, GNAI2 and GNAI3. Cys66 was highlighted with red color and the adjacent amino was highlighted with green color. The blue color indicates the α-helix where Cys66 resides in. (e) HUVECs were transfected with pcDNA, GNAI2-WT

and GNAI2-R67K plasmids, followed by HG and oxLDL stimulation for 24 h. Mutation of Arg 67 of GNAI2 to Lys 67 partially inhibits the level of S-nitrosylated GNAI2. n=3 distinct samples for each group. N.D represents no detected. Data are represented as the Mean ± SEM. One-way ANOVA followed by Turkey's test for post hoc comparisons. Source data are provided as a Source Data file.

Supplementary Figure 2

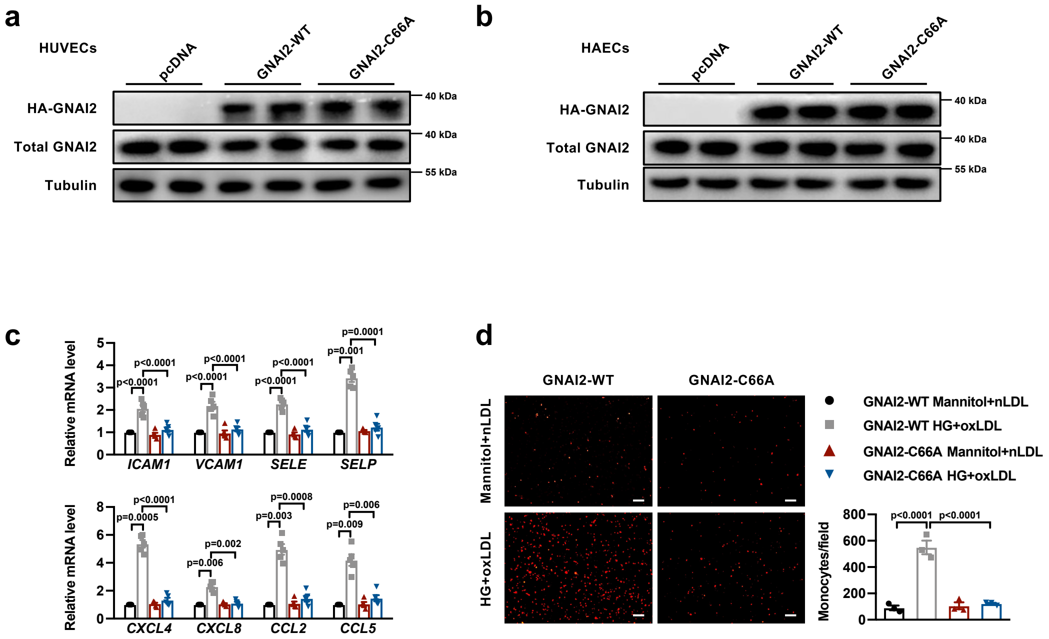

Supplementary Figure 2: Inhibition of S-nitrosylation of GNAI2 attenuates endothelial inflammation in HG- and oxLDL-treated HUVECs. (a, b) The GNAI2-WT and GNAI2-C66A were successfully over-expressed in HUVECs or HAECs as evidenced by HA-GNAI2, n=4 indepenent samples for each group. (c) HUVECs were ectopically expressed with GNAI2-WT and GNAI2-C66A, followed by stimulated with HG and oxLDL for 24 h. GNAI2-C66A inhibits the mRNA expressions of adhesion molecules (*ICAM1*, *VCAM1*, *SELE* and *SELP*) and chemokines (*CXCL4*, *CXCL8*, *CCL2* and *CCL5*) induced by HG and oxLDL as determined by qPCR. n=5 distinct samples for each group. (d) GNAI2-C66A

prevents the attachment of THP-1 cells to HG- and oxLDL-treated HUVECs. Scale bar=100 µm. n=3 distinct samples for each group. Data are represented as the Mean ± SEM. (c) One-way ANOVA followed by Turkey's test for post hoc comparisons was used for analysis of *ICAM1*, *VCAM1* and *SELE*, Welch ANOVA followed by Tamhane's T2 test for post hoc comparisons was used for *SELP*, *CXCL4*, *CXCL8*, *CCL2* and *CCL5*. (d) One-way ANOVA followed by Turkey's test for post hoc comparisons. Source data are provided as a Source Data file.

Supplementary Figure 3

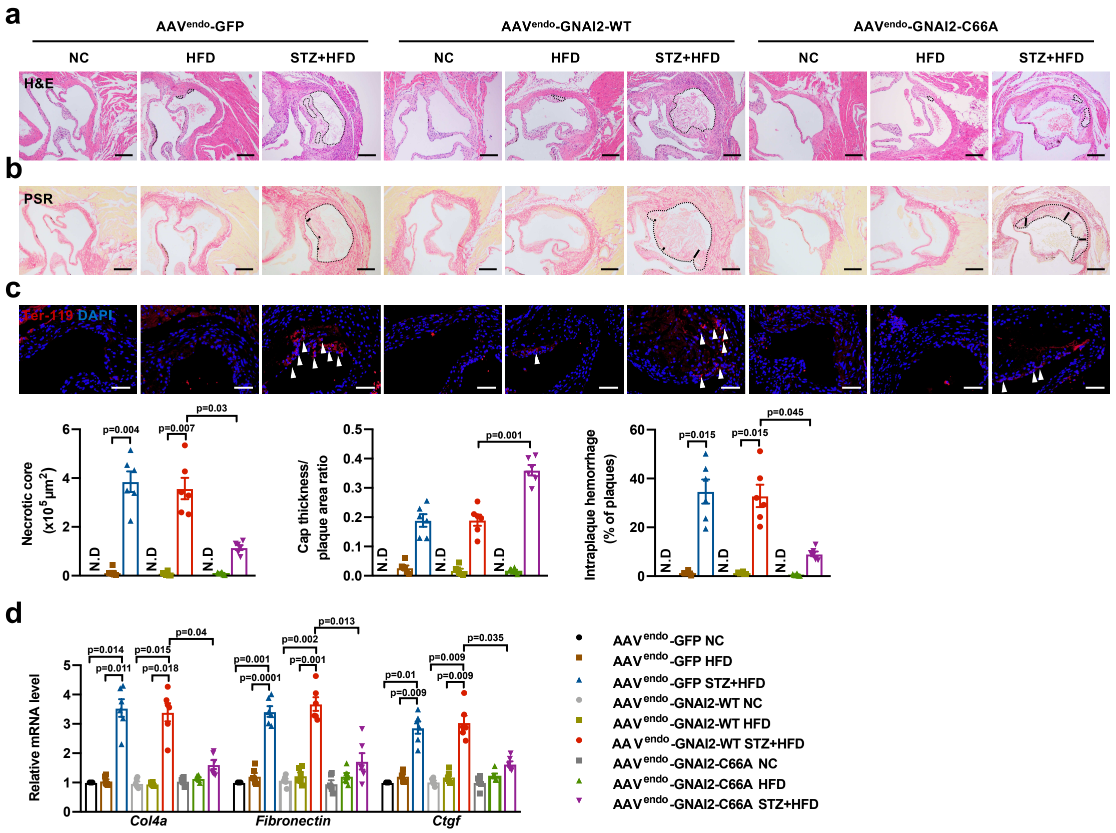

Supplementary Figure 3: Inhibition of SNO-GNAI2 restrains the development of diabetic atherosclerosis and enhances the plaque stability in treated LDLr$^{-/-}$ mice. LDLr$^{-/-}$ mice were transduced with AAV^{endo}-GFP, AAV^{endo}-GNAI2-WT or AAV^{endo}-GNAI2-C66A respectively, and randomly separated into NC, HFD and STZ+HFD groups. (a) Endothelial

specific transfection of GNAI2-C66A reduces the necrotic core areas induced by STZ+HFD. Necrotic cores were indicated with black lines. scale bar=200 μm. n=6 mice for each group. N.D represents no detected. (b) Endothelial specific transfection of GNAI2-C66A increases the fibrous cap thickness of plaques compared to AAV^{endo}-GNAI2-WT STZ+HFD groups. n=6 mice for each group. N.D represents no detected. (c) Endothelial specific transfection of GNAI2-C66A attenuates the intraplaque hemorrhage as demonstrated by immunofluorescent staining of Ter-119. scale bar=50 μm. n=6 mice for each group. N.D represents no detected. (d) AAV^{endo}-GNAI2-C66A decreases the mRNA levels of *Col4a*, *Fibronectin* and *Ctgf* in diabetes-accelerated atherosclerosis. n=6 mice for each group. Data are represented as the Mean ± SEM. Welch ANOVA followed by Tamhane's T2 test for post hoc comparisons. Source data are provided as a Source Data file.

Supplementary Figure 4

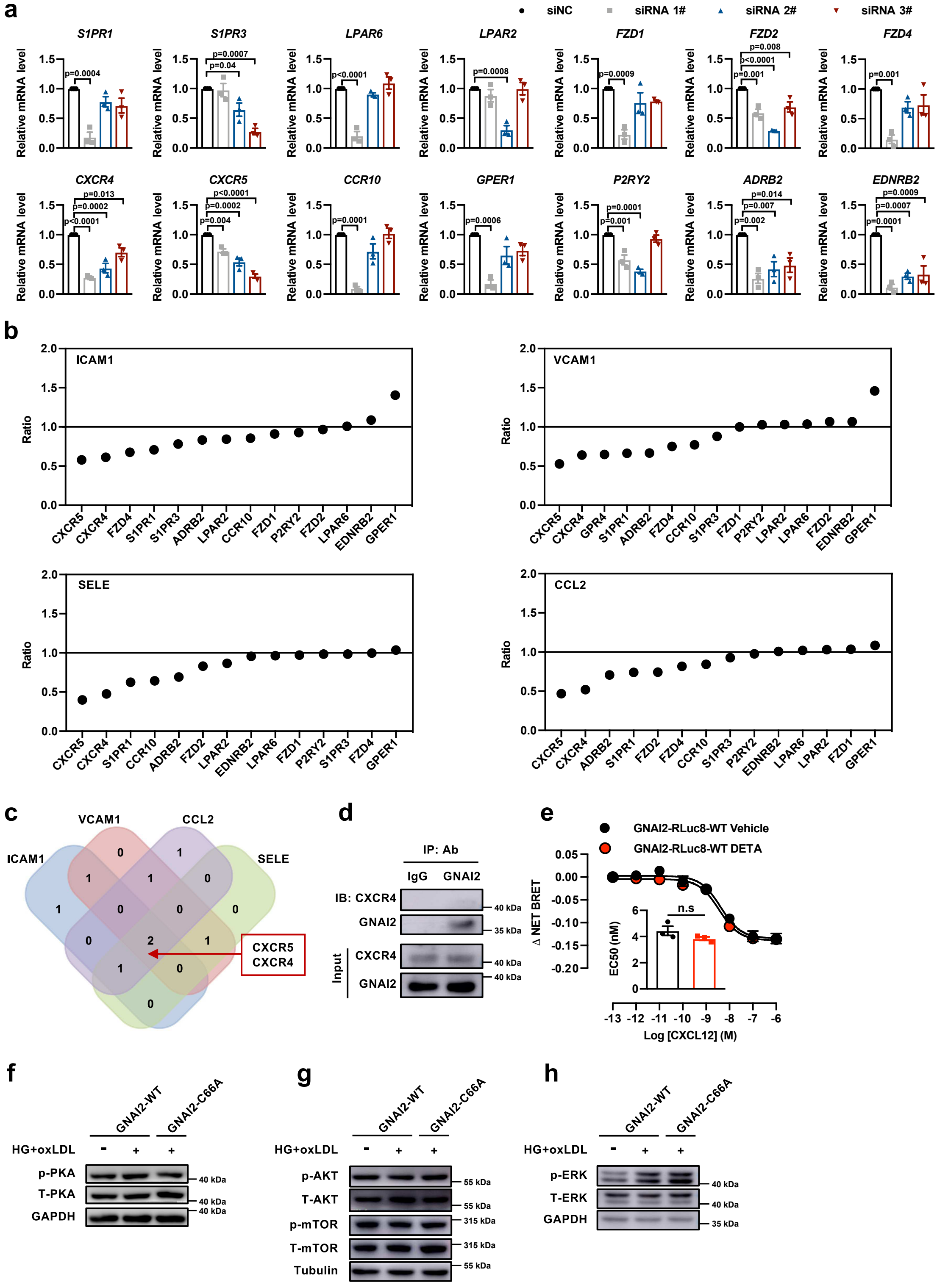

Supplementary Figure 4: CXCR5 is indentified as recepotrs mediating endothelial inflammation and S-nitrosylation of GNAI2 at Cys66 does not affect classical GPCR signaling pathways in HG- and oxLDL-treated HUVECs. (a) HUVECs were transfected with siRNAs against 14 selected Gi-coupled receptors. The knockdown efficiency was

verified by qPCR. n=3 independent experiments. (b) siRNA screening to identify GPCRs that mediate HG+oxLDL-induced expression of *ICAM1*, *VCAM1*, *SELE* and *CCL2* in HUVECs. Shown is the ratio of the effects on *ICAM1*, *VCAM1*, *SELE* and *CCL2* in HUVECs transfected with siRNA against a GPCR and HUVECs transfected with siNC. n=3 for each group. (c) Only knockdown of CXCR4 or CXCR5 can inhibit all four inflammatory factors. (d) The endogenous interaction between GNAI2 and CXCR4. One indepenent experiment was performed. (e) HEK293T cells were transfected with GNAI2-RLuc8-WT, followed by treatment with a NO donor, DETA (500 μM). The BRET assay shows that SNO-GNAI2 does not affect the response of CXCL12 to DETA. n=3 distinct samples for each group. n.s represents no significance. (f) GNAI2-C66A does not affect the levels of phospho-PKA and total PKA protein. n=3 distinct samples for each group. (g) GNAI2-C66A does not affect the AKT/mTOR signaling pathway as evidenced by phosphorylated and total AKT and mTOR in HG- and oxLDL-treated HUVECs. n=4 distinct samples for each group. (h) GNAI2-C66A does not rescue the increased levels of phospho-ERK1/2 in HUVECs treated with HG+oxLDL. n=4 distinct samples for each group. Data are represented as the Mean ± SEM. (a) One-way ANOVA followed by Dunnett’s test for post hoc comparisons was used for statistical analysis. (e) Unpaired two-tailed Student’s t test was used for statistical analysis. Source data are provided as a Source Data file.

Supplementary Figure 5

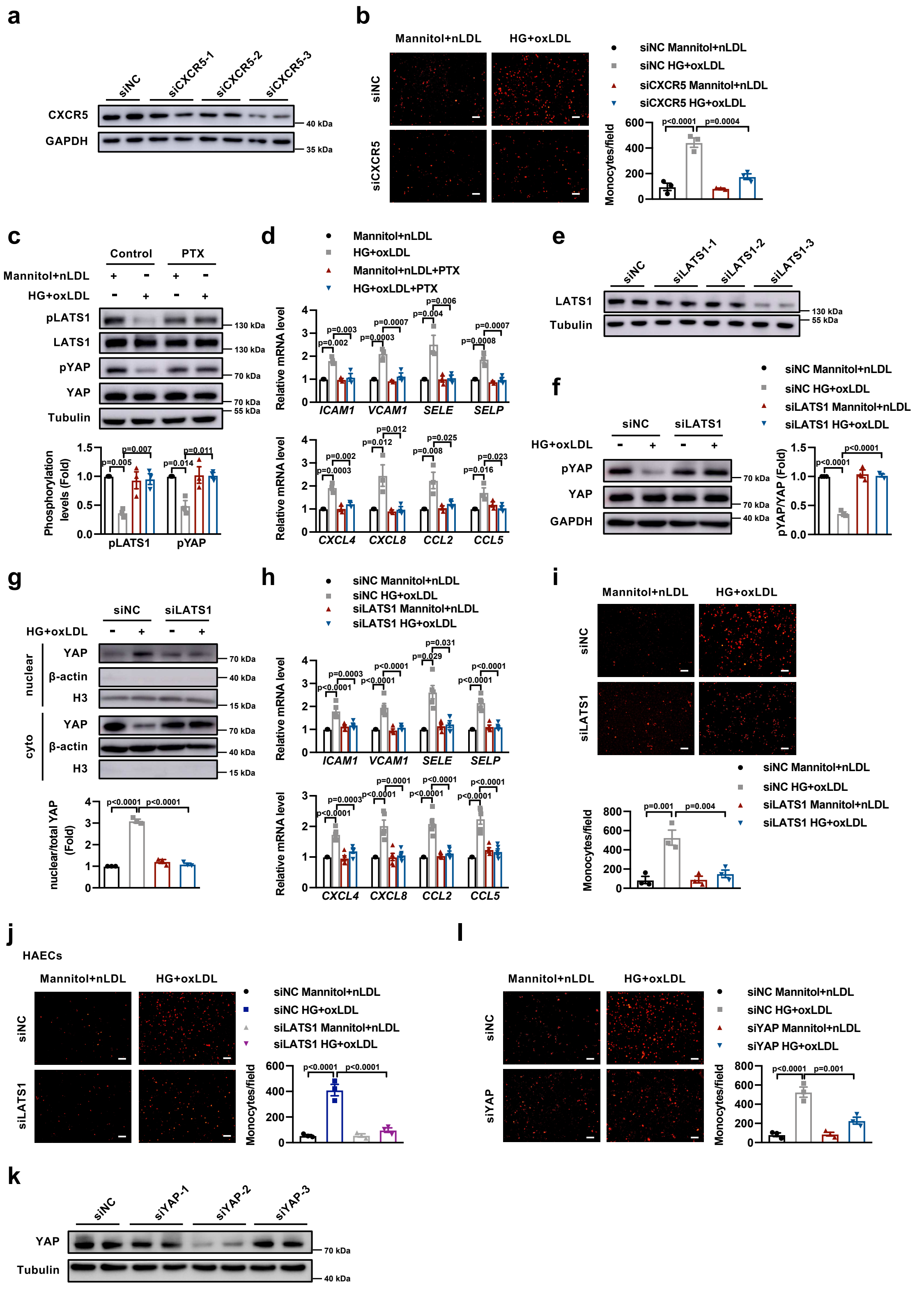

Supplementary Figure 5: Inactivation of CXCR5-Hippo-YAP pathway improves endothelial inflammation and monocyte adhesion induced by HG and oxLDL. (a) HUVECs were transfected with siNC or siCXCR5. The knockdown efficiency of CXCR5

was detected by western blot analysis. Two distinct cell samples were performed. (b) Knockdown of CXCR5 inhibits the attachment of THP-1 monocytes to ECs treated with HG and oxLDL. Scale bar=100 μm. n=3 independent experiments. (c, d) HUVECs were preincubated with Pertussis toxin (PTX, 100 ng/mL) before treated with HG+oxLDL. (c) PTX restores the phosphorylation of LATS1 and YAP. n=3 distinct samples for each group. (d) PTX reduces the expression of inflammatory genes in HUVECs stimulated with HG and oxLDL. n=3 distinct samples for each group. (e) The knockdown efficiency of LATS1 was detected by western blot analysis. Two distinct cell samples were performed. (f) Knockdown of LATS1 restores the phosphorylation of YAP (n=3 independent experiments), (g) reduces YAP nuclear translocation (n=3 independent experiments), (h) suppresses the expression of adhesion molecules and chemokines (n=5 independent experiments) and (i) reduces the attachment of THP-1 monocytes to HUVECs treated with HG and oxLDL (n=3 independent experiments). Scale bar=100 μm. (j) Knockdown of LATS1 reduces the attachment of THP-1 monocytes to HAECs treated with HG and oxLDL. Scale bar=100 μm. n=3 distinct samples for each group. (k) The knockdown efficiency of YAP was detected by western blot analysis in HUVECs. Two distinct cell samples were performed. (l) Knockdown of YAP decreases the attachment of THP-1 cells to HG- and oxLDL-stimulated HUVECs. Scale bar=100 μm. n=3 distinct samples for each group. Data are represented as the Mean ± SEM. One-way ANOVA followed by Turkey's test for post hoc comparisons was used. For *SELE* in (h), Welch ANOVA followed by Tamhane's T2 test for post hoc comparisons was used. Source data are provided as a Source Data file.

Supplementary Figure 6

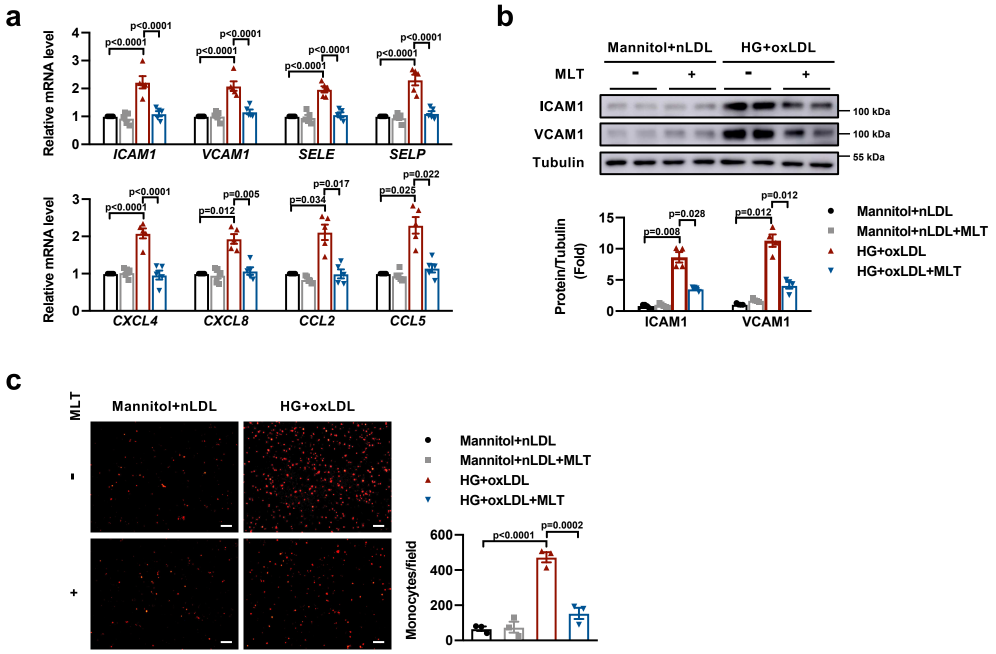

Supplementary Figure 6: Melatonin inhibits the endothelial inflammation in HUVECs treated with HG and oxLDL. (a) Melatonin abolishes the elevation of adhesion molecules and chemokines in HUVECs stimulated by HG+oxLDL. n=5 distinct samples for each group. (b) Melatonin suppresses the protein levels of ICAM1 and VCAM1. n=4 distinct samples for each group. (c) Melatonin attenuates the adhesion of THP-1 cells to HUVECs treated with HG+oxLDL. Scale bar=100 μm. n=3 distinct samples for each group. Data are represented as the Mean ± SEM. (a) One-way ANOVA followed by Turkey's test for post hoc comparisons was used for *ICAM1*, *VCAM1*, *SELE*, *SELP* and *CXCL4*. Welch ANOVA followed by Tamhane's T2 test for post hoc comparisons was used for *CXCL8*, *CCL2*, *CCL5*. (b) Welch ANOVA followed by Tamhane's T2 test for post hoc comparisons. (c) One-way ANOVA followed by Turkey's test for post hoc comparisons. Source data are provided as a Source Data file.

Supplementary Figure 7

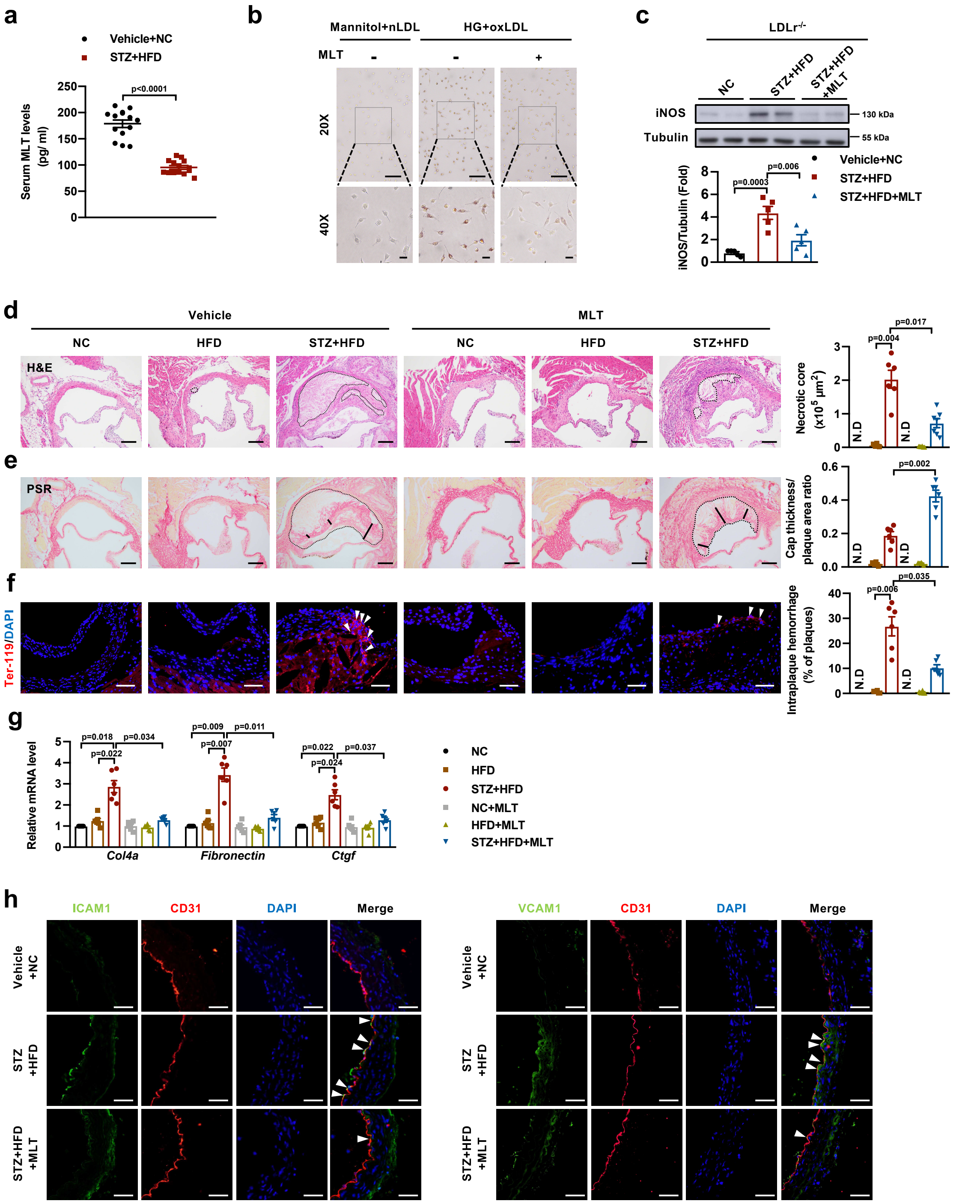

Supplementary Figure 7: Melatonin attenuates the development of diabetes-accelerated atherosclerosis in STZ and HFD-treated LDLr$^{-/-}$ mice. (a) The serum levels of melatonin in STZ+HFD-treated mice were significantly lower than those in controls. n=14 mice samples for each group. (b) Foam cell formation as determined by Oil Red O-staining. Scale

bar=100 μm. Three distinct cell samples were performed. (c) Melatonin decreases the expression of iNOS in aortas of STZ+HFD-treated LDLr$^{-/-}$ mice. n=5 mice for each group. (d) Melatonin attenuates the necrotic cores areas in aortic roots induced by STZ+HFD. Necrotic cores were indicated with black lines. scale bar=200 μm. n=7 mice in STZ+HFD+MLT group and n=6 mice in other groups. (e) Melatonin increases the fibrous cap thickness of plaques compared with those in STZ+HFD-treated group. scale bar=200 μm. n=6 mice for each group. (f) Melatonin reduces the intraplaque hemorrhage in STZ+HFD-treated LDLr$^{-/-}$ mice as demonstrated by immunofluorescent staining of Ter-119. Scale bar=50 μm, Ter-119 (red). n=6 mice for each group. (g) Melatonin reduces the mRNA levels of *Col4a*, *Fibronectin* and *Ctgf* in aortas of STZ+HFD-treated LDLr$^{-/-}$ mice. n=6 mice for each group. (h) ICAM1 and VCAM1 staining in the endothelium of STZ+HFD-treated LDLr$^{-/-}$ mice. Scale bar=100 μm, ICAM1 (green), VCAM1 (green), CD31 (red). n=3 mice samples for each group. N.D represents no detected. Data are represented as the Mean ± SEM. (a) Mann-Whitney test. (c) One-way ANOVA followed by Turkey's test for post hoc comparisons. (d, e, f, g) Welch ANOVA followed by Tamhane's T2 test for post hoc comparison. Source data are provided as a Source Data file.
