# Supplementary material for: S-nitrosylation-mediated coupling of G-protein alpha-2 with CXCR5 induces Hippo/YAP-dependent diabetes-accelerated atherosclerosis
Source: Nat Commun. 2021 Jul 22;12:4452. doi: 10.1038/s41467-021-24736-y (PMC8298471; doi:10.1038/s41467-021-24736-y)
Supplement: Supplementary file 2 — Reporting Summary [file 41467_2021_24736_MOESM2_ESM.pdf]

nature portfolio

Corresponding author(s): Yong Ji, Liping Xie, Yi Han

Last updated by author(s): June 8, 2021

Reporting Summary

Nature Portfolio wishes to improve the reproducibility of the work that we publish. This form provides structure for consistency and transparency in reporting. For further information on Nature Portfolio policies, see our Editorial Policies and the Editorial Policy Checklist.

Statistics

For all statistical analyses, confirm that the following items are present in the figure legend, table legend, main text, or Methods section.

n/a	Confirmed	
☐	☒	The exact sample size (*n*) for each experimental group/condition, given as a discrete number and unit of measurement
☐	☒	A statement on whether measurements were taken from distinct samples or whether the same sample was measured repeatedly
☐	☒	The statistical test(s) used AND whether they are one- or two-sided<br>*Only common tests should be described solely by name; describe more complex techniques in the Methods section.*
☒	☐	A description of all covariates tested
☐	☒	A description of any assumptions or corrections, such as tests of normality and adjustment for multiple comparisons
☐	☒	A full description of the statistical parameters including central tendency (e.g. means) or other basic estimates (e.g. regression coefficient) AND variation (e.g. standard deviation) or associated estimates of uncertainty (e.g. confidence intervals)
☐	☒	For null hypothesis testing, the test statistic (e.g. *F*, *t*, *r*) with confidence intervals, effect sizes, degrees of freedom and *P* value noted<br>*Give P values as exact values whenever suitable.*
☒	☐	For Bayesian analysis, information on the choice of priors and Markov chain Monte Carlo settings
☒	☐	For hierarchical and complex designs, identification of the appropriate level for tests and full reporting of outcomes
☒	☐	Estimates of effect sizes (e.g. Cohen's *d*, Pearson's *r*), indicating how they were calculated

Our web collection on statistics for biologists contains articles on many of the points above.

Software and code

Policy information about availability of computer code

Data collection	American Image 600 (AI600, GE, USA) was used for western blot data collection. ZEISS LSM 800 confocal microscope (Germany) was used for immunofluorescent staining data collection. QuantStudio Design and Analysis software version 1.5.1 (ABI, USA) was used for real-time PCR data collection.
Data analysis	The Statistical analyses were performed with Graphpad Prism software version 8.0 (GraphPad Software, Inc., San Deigo, CA, USA). Image J software version 1.53 (National Institutes of Health, USA) was for western blot analysis and Image-Pro Plus 6.0 software (Media Cybernetics, USA) was for histological staining analyses.

For manuscripts utilizing custom algorithms or software that are central to the research but not yet described in published literature, software must be made available to editors and reviewers. We strongly encourage code deposition in a community repository (e.g. GitHub). See the Nature Portfolio guidelines for submitting code & software for further information.

Data

Policy information about availability of data

All manuscripts must include a data availability statement. This statement should provide the following information, where applicable:

- Accession codes, unique identifiers, or web links for publicly available datasets
- A description of any restrictions on data availability
- For clinical datasets or third party data, please ensure that the statement adheres to our policy

Data Availability. Raw RNA-sequence data for GPCRs expression in HUVECs were deposited at the GEO database https://www.ncbi.nlm.nih.gov/geo/query/acc.cgi?acc=GSE173669 with accession codes GSE173669. Mass Spectrometry data for protein S-nitrosylation have been deposited to the ProteomeXchange Consortium via

the PRIDE partner repository with the dataset identifier PXD025295 with hyperlinks throughout http://proteomecentral.proteomexchange.org/cgi/GetDataset?ID=PXD025295. Raw data of all figures and uncropped versions of any gels or blots presented in the figures are provided as a Source Data file.

Field-specific reporting

Please select the one below that is the best fit for your research. If you are not sure, read the appropriate sections before making your selection.

☒ Life sciences ☐ Behavioural & social sciences ☐ Ecological, evolutionary & environmental sciences

For a reference copy of the document with all sections, see nature.com/documents/nr-reporting-summary-flat.pdf

Life sciences study design

All studies must disclose on these points even when the disclosure is negative.

Sample size	We did not use statistical method to predetermine sample size. The sample size was based on the previous literature that a minimum of N = 3 biological replicates with sufficient reproducibility in cell experiments and a minimum of N = 5 biological replicates with sufficient reproducibility in animal experiments (PMID: 31902237; PMID: 30405100).
Data exclusions	We did not exclude any data.
Replication	All the experiments have been at least replicated for three independent times. All the results have been validated in the independent repeated experiments.
Randomization	The mice were randomly divided into different groups. For each independent cell experiment, cells were passed to enough culture dishes, and randomly stimulated with different treatments.
Blinding	The investigators were blinded to group allocations during data collection and analysis.

Reporting for specific materials, systems and methods

We require information from authors about some types of materials, experimental systems and methods used in many studies. Here, indicate whether each material, system or method listed is relevant to your study. If you are not sure if a list item applies to your research, read the appropriate section before selecting a response.

Materials & experimental systems

n/a	Involved in the study	
☐	☒	Antibodies
☐	☒	Eukaryotic cell lines
☒	☐	Palaeontology and archaeology
☐	☒	Animals and other organisms
☐	☒	Human research participants
☒	☐	Clinical data
☒	☐	Dual use research of concern

Methods

n/a	Involved in the study	
☒	☐	ChIP-seq
☒	☐	Flow cytometry
☒	☐	MRI-based neuroimaging

Antibodies

Antibodies used

Antibodies for western blot
Antibody Cat No. Manufacturer Sources of species Concentration
ICAM1 SC8439 Santa Cruz Mouse 1:500
VCAM1 SC13160 Santa Cruz Rabbit 1:500
iNOS ab178945 Abcam Rabbit 1:1000
p-eNOS 9571 CST Rabbit 1:1000
eNOS 610297 BD Mouse 1:1000
Trx ab86255 Abcam Rabbit 1:1000
GSNOR ab177932 Abcam Rabbit 1:1000
GNAI2 11136-1-AP Proteintech Rabbit 1:1000
CXCR5 ab133706 Abcam Rabbit 1:1000
pLATS1 9157 CST Rabbit 1:2000
LATS1 DF7517 Affitiny Rabbit 1:2000
pYAP 13008 CST Rabbit 1:1000
YAP 14074 CST Rabbit 1:1000
pAKT 9271 CST Rabbit 1:1000
AKT 9272 CST Rabbit 1:1000
p-mTOR 2974 CST Rabbit 1:1000
mTOR 2983 CST Rabbit 1:1000

pERK1/2 9101 CST Rabbit 1:1000
ERK1/2 9102 CST Rabbit 1:1000
Tubulin BS1482M Bioworld Mouse 1:5000
β-actin AP0060 Bioworld Rabbit 1:3000
GAPDH AP0063 Bioworld Rabbit 1:5000
H3 9715 CST Rabbit 1:3000

Antibodies for Immunofluorescence staining
Antibody Cat No. Manufacturer Sources of species concentration
anti-CD68 GB11067 Servicebio Rabbit 1:100
anti-ICAM1 SC8439 Santa Cruz Mouse 1:100
anti-VCAM1 SC13160 Santa Cruz Mouse 1:50
anti-GNAI2 11136-1-AP Proteintech Rabbit 1:100
anti-CXCR5 SC373775 Santa Cruz Mouse 1:100
anti-YAP 14074 CST Rabbit 1:100
anti-CD31 AF3628 R&D system Goat 1:100
anti-Ter119 13-5921-82 Invitrogen Mouse 1:100
anti-αSMA ab124964 Abcam Rabbit 1:100

Validation

Validation statements for antibodies can be found on their corresponding manufacturer websites.
Anti-ICAM1: Species Reactivity: Mouse, Rat and Human. Applications: WB, IP, IF, IHC, Flow cytometry and ELISA.
Anti-VCAM1: Species Reactivity: Mouse, Rat and Human. Applications: WB, IP, IF, IHC, Flow cytometry and ELISA.
Anti-iNOS: Species Reactivity: Mouse, Rat and Human. Applications: ELISA, WB, ICC/IF and IP.
Anti-p-eNOS: Species Reactivity: Human, Bovine and Pig. Applications: WB.
Anti-eNOS: Species Reactivity: Human (QC Testing), Mouse, Rat (Tested in Development). Applications: WB (Routinely Tested), IP, IF and IHC (Tested During Development).
Anti-Trx: Species Reactivity: Human. Applications: WB.
Anti-GSNOR: Species Reactivity: Mouse, Rat and Human. Applications: WB and IHC-P.
Anti-GNAI2: Species Reactivity: human, mouse, rat. Applications: IF, IHC, IP, WB,ELISA. Positive WB detected in:fetal human brain tissue, SKOV-3 cells, mouse brain tissue, rat brain tissue. Positive IP detected in:mouse brain tissue.
Anti-CXCR5: Species Reactivity: Human. Applications: ICC/IF, Flow cytometry, IHC-P, IP and WB.
Anti-pLATS1: Species Reactivity: human. Applications: WB.
Anti-LATS1: Species Reactivity: Human and Mosue. Applications: WB, IHC, ICC/IF and ELISA (peptide).
Anti- pYAP: Species Reactivity: Human, Mouse, Rat. Applications: WB, IP and IHC-P.
Anti-YAP: Species Reactivity: Human, Mouse, Rat, Hamster and Monkey. Applications: WB, IP, IHC, IHC-P, ICC/IF, Flow cytometry, ChIP, ChIP-Seq and CUT&RUN.
Anti-pAKT: Species Reactivity: Human, Mouse, Rat, Hamster, Monkey, D. melanogaster, Bovine and Dog. Applications: WB, IP, ICC/IF and Flow cytometry.
Anti-AKT: Species Reactivity: Human, Mouse, Rat, Hamster, Monkey, Chicken, D. melanogaster, Bovine, Dog, Pig and Guinea Pig. Applications: WB, IP, ICC/IF and Flow cytometry.
Anti-p-mTOR: Species Reactivity: Human, Mouse, Rat and Monkey. Applications: WB.
Anti-mTOR: Species Reactivity: Human, Mouse, Rat and Monkey. Applications: WB, IHC-P, ICC/IF and Flow cytometry.
Anti-pERK1/2: Species Reactivity: Human, Mouse, Rat, Hamster, Monkey, Mink, D. melanogaster, Zebrafish, Bovine, Pig and C. elegans. Applications: WB, IP, ICC/IF and Flow cytometry.
Anti-ERK1/2: Species Reactivity: Human, Mouse, Rat, Hamster, Monkey, Mink, Zebrafish, Bovine, Pig and S. cerevisiae. Applications: WB, IP and ICC/IF.
Anti-Tubulin: Species Reactivity: Human, Mouse, Rabbit, Frog, Fish, Chicken and Rat. Applications: WB and IHC.
Anti-β-actin: Species Reactivity: Human, Mouse and Rat. Applications: WB.
Anti-GAPDH: Species Reactivity: Human, Mouse and Rat. Applications: WB.
Anti-H3: Species Reactivity: Human, Mouse, Rat, Monkey, Zebrafish, Bovine and Pig. Applications: WB.

Anti-CD68: Species Reactivity: Mouse, Rat. Applications: IHC, IF.
Anti-ICAM1: Species Reactivity: Mouse, Rat and Human. Applications: WB, IP, IF, IHC, Flow cytometry and ELISA.
Anti-VCAM1: Species Reactivity: Mouse, Rat and Human. Applications: WB, IP, IF, IHC, Flow cytometry and ELISA.
Anti-GNAI2: Species Reactivity: human, mouse, rat. Applications: IF, IHC, IP, WB,ELISA. Positive WB detected in:fetal human brain tissue, SKOV-3 cells, mouse brain tissue, rat brain tissue. Positive IP detected in:mouse brain tissue.
Anti-CXCR5: Species Reactivity: Mouse, Rat and Human. Applications: WB, IP, IF and ELISA.
Anti-YAP: Species Reactivity: Human, Mouse, Rat, Hamster and Monkey. Applications: WB, IP, IHC, Flow cytometry, ChIP, ChIP-Seq and CUT&RUN.
Anti-CD31: Species Reactivity: Mouse and Rat. Applications: WB, Flow cytometry, IHC, CyTOF-ready and ICC/IF.
Anti-Ter119: Species Reactivity: Mouse. Published Species: Fish, Mouse and Human. Applications: IHC, Flow cytometry, Functional Assay, ICC/IF and Misc.
Anti-αSMA: Species Reactivity: Mouse, Rat, Human and Simian Virus 40. Applications: WB, IHC-P, Flow cytometry and ICC/IF.

Eukaryotic cell lines

Policy information about cell lines

Cell line source(s)

Human umbilical vein endothelial cells (HUVECs) were isolated from human umbilical cords obtained from the Sir Run Run Hospital. Human aortic endothelial cells (HAECs) were a gift from Prof. Yong Xu (Nanjing Medical University). Mouse aortic endothelial cells (MAECs) were isolated from CXCR5 knockout mice from Nantong University. THP-1 monocytes were

	purchased from National Infrastructure of Cell Line Resource (Shanghai, China). HEK293T cells were a gift from Prof. Jin-Peng Sun (Shandong University).
Authentication	No authentication has been used.
Mycoplasma contamination	The cells were tested negative for mycoplasma contamination.
Commonly misidentified lines (See ICLAC register)	None of the cells is listed in ICLAC.

Animals and other organisms

Policy information about studies involving animals; ARRIVE guidelines recommended for reporting animal research

Laboratory animals	6- to 8- week-old male LDLr knockout mice and 6-week-old male CXCR5 knockout mice were used in this project.
Wild animals	The study did not involve wild animals.
Field-collected samples	The study did not involve samples collected from the field.
Ethics oversight	All animal experiments were conducted according to the ARRIVE guidelines for the care and use of laboratory animals, protocols were approved by the Animal Care and Use Committee of Nanjing Medical University (IACUC-1811027).

Note that full information on the approval of the study protocol must also be provided in the manuscript.

Human research participants

Policy information about studies involving human research participants

Population characteristics	Male Coronary artery disease (CAD) patients combined with diabetes subjected to coronary artery bypass grafting between age of 55y and 75y. Male Coronary artery disease (CAD) patients without diabetes subjected to coronary artery bypass grafting between age of 34y and 75y.
Recruitment	Tissue samples were collected from patients who had signed informed consent form. There were no self-selection bias or other biases that are likely to impact results.
Ethics oversight	All procedures involving sampling were performed according to the principles outlined in the Declaration of Helsinki and were approved by the Ethics Committee of the Affiliated Drum Tower Hospital of Nanjing University Medical School (2019-219-01).

Note that full information on the approval of the study protocol must also be provided in the manuscript.
