# Supplementary material for: S-nitrosylation-mediated coupling of G-protein alpha-2 with CXCR5 induces Hippo/YAP-dependent diabetes-accelerated atherosclerosis
Source: Nat Commun. 2021 Jul 22;12:4452. doi: 10.1038/s41467-021-24736-y (PMC8298471; doi:10.1038/s41467-021-24736-y)
Supplement: Supplementary file 3 — Source Data [file 41467_2021_24736_MOESM3_ESM.zip › Souece data/Full set of western blots.pptx]

## Slide 1
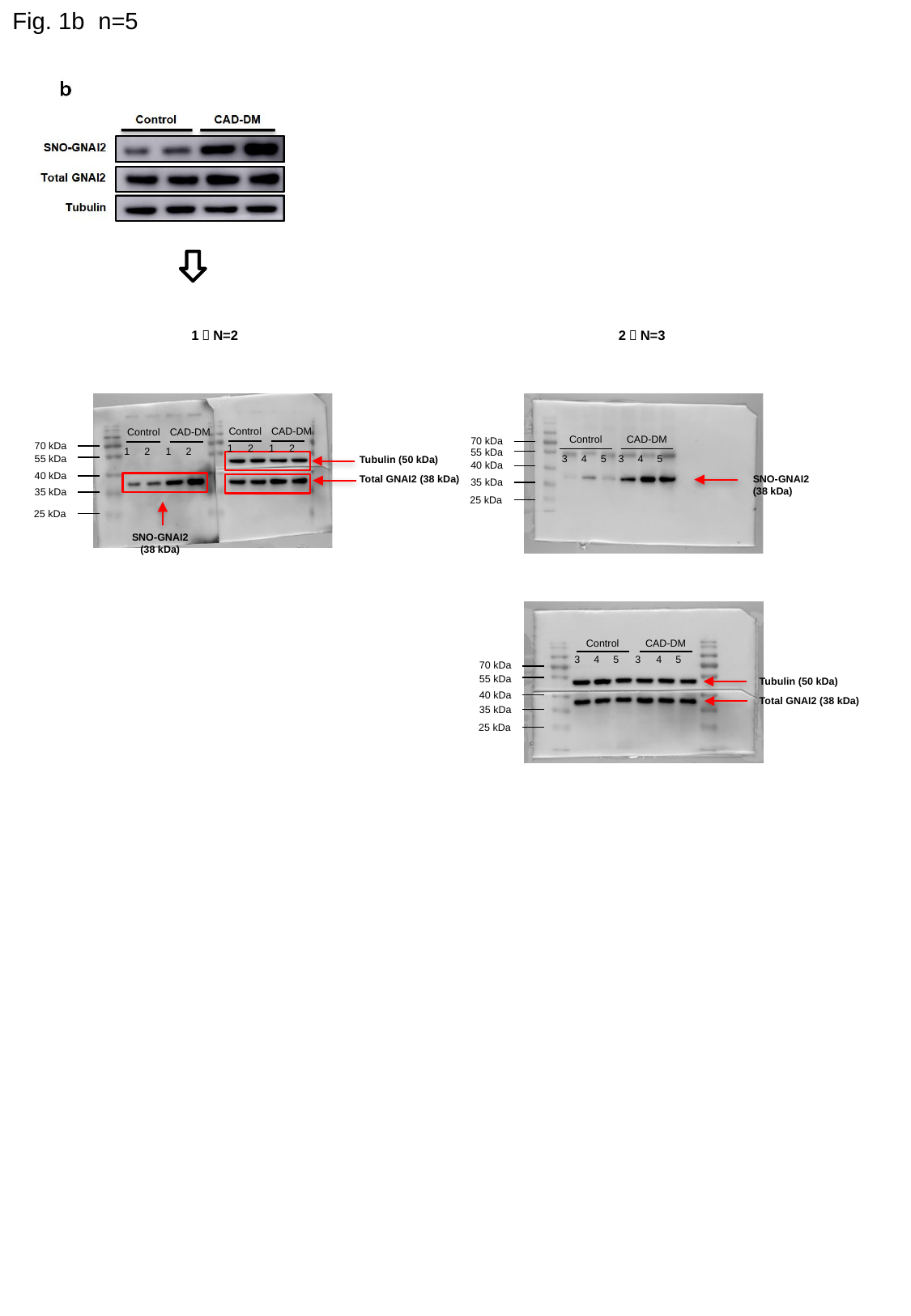

Fig. 1b n=5
1：N=2
2：N=3
Control
CAD-DM
Control
CAD-DM
70 kDa
1
2
1
2
1
2
1
2
55 kDa
Tubulin (50 kDa)
40 kDa
Total GNAI2 (38 kDa)
35 kDa
25 kDa
SNO-GNAI2
(38 kDa)
Control
CAD-DM
70 kDa
55 kDa
3
4
5
3
4
5
40 kDa
SNO-GNAI2
(38 kDa)
35 kDa
25 kDa
Control
CAD-DM
3
4
5
3
4
5
70 kDa
55 kDa
Tubulin (50 kDa)
40 kDa
Total GNAI2 (38 kDa)
35 kDa
25 kDa

## Slide 2
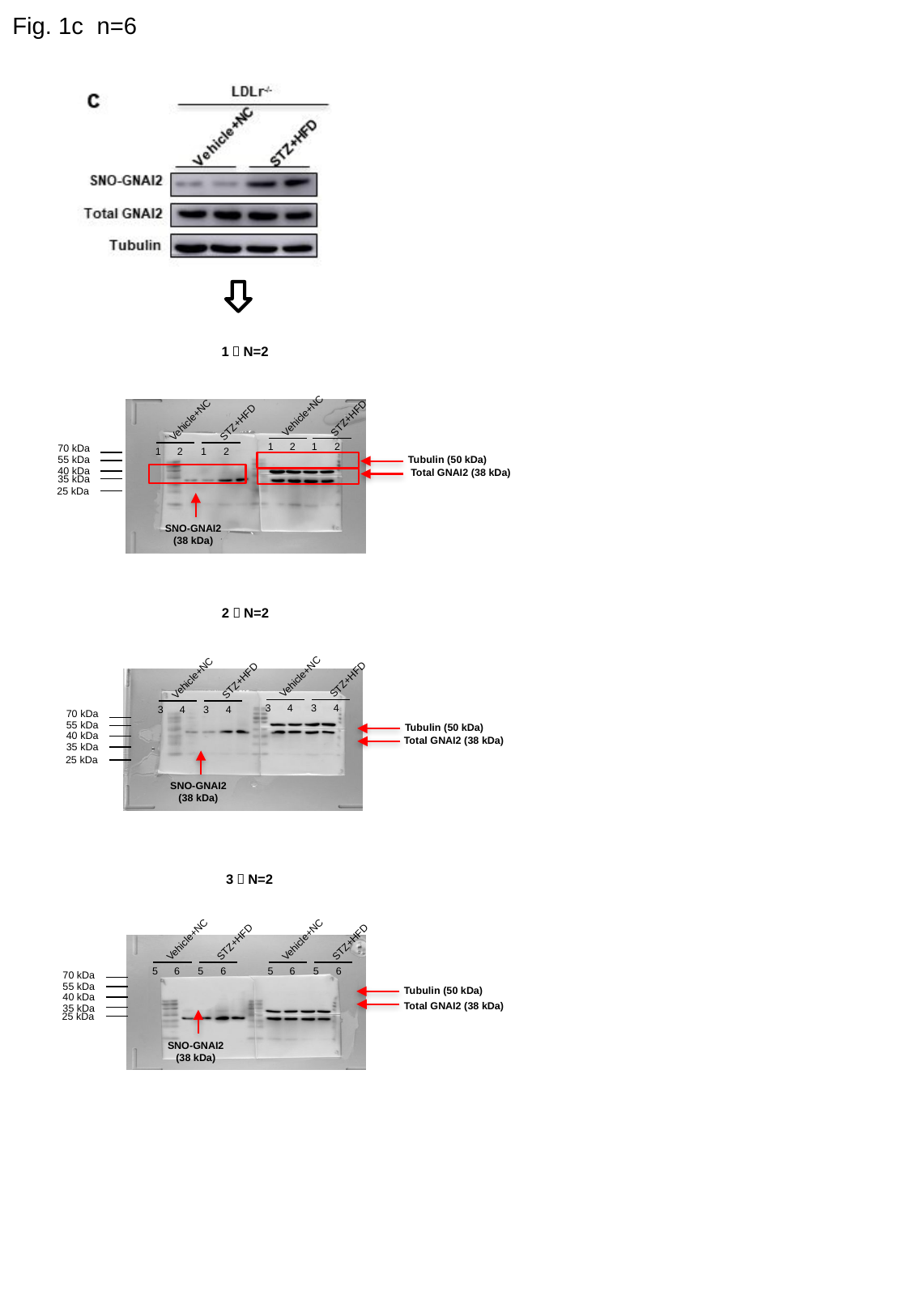

Fig. 1c n=6
1：N=2
Vehicle+NC
STZ+HFD
Vehicle+NC
STZ+HFD
1
2
1
2
70 kDa
1
2
1
2
Tubulin (50 kDa)
55 kDa
40 kDa
 Total GNAI2 (38 kDa)
35 kDa
25 kDa
SNO-GNAI2
(38 kDa)
2：N=2
Vehicle+NC
STZ+HFD
Vehicle+NC
STZ+HFD
3
4
3
4
3
4
3
4
70 kDa
55 kDa
Tubulin (50 kDa)
40 kDa
 Total GNAI2 (38 kDa)
35 kDa
25 kDa
SNO-GNAI2
(38 kDa)
3：N=2
Vehicle+NC
STZ+HFD
Vehicle+NC
STZ+HFD
5
6
5
6
5
6
5
6
70 kDa
55 kDa
Tubulin (50 kDa)
40 kDa
 Total GNAI2 (38 kDa)
35 kDa
25 kDa
SNO-GNAI2
(38 kDa)

## Slide 3
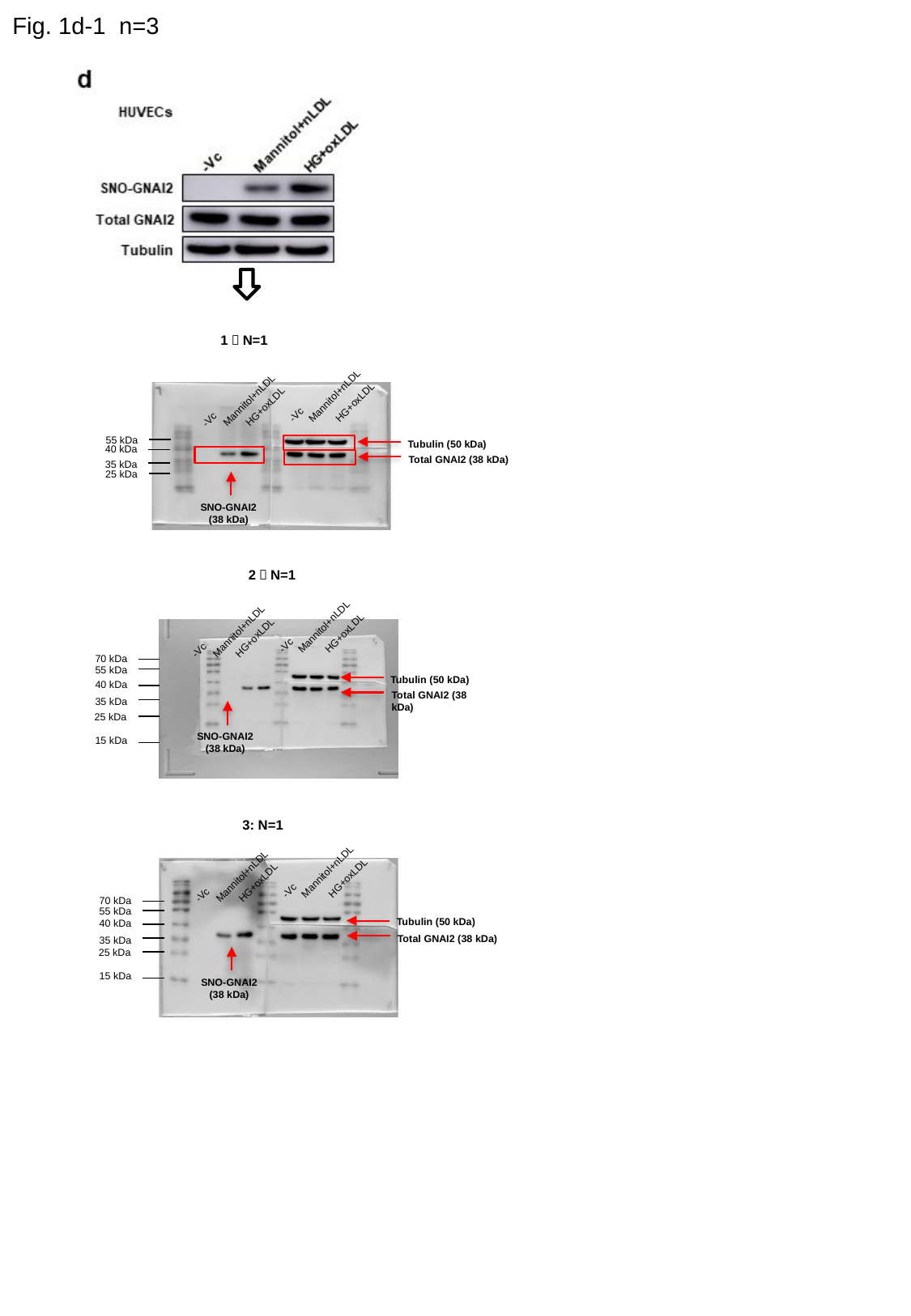

Fig. 1d-1 n=3
1：N=1
Mannitol+nLDL
HG+oxLDL
Mannitol+nLDL
HG+oxLDL
-Vc
-Vc
55 kDa
Tubulin (50 kDa)
40 kDa
Total GNAI2 (38 kDa)
35 kDa
25 kDa
SNO-GNAI2
(38 kDa)
2：N=1
Mannitol+nLDL
HG+oxLDL
Mannitol+nLDL
HG+oxLDL
-Vc
-Vc
70 kDa
55 kDa
Tubulin (50 kDa)
40 kDa
Total GNAI2 (38 kDa)
35 kDa
25 kDa
SNO-GNAI2
(38 kDa)
15 kDa
3: N=1
Mannitol+nLDL
HG+oxLDL
Mannitol+nLDL
HG+oxLDL
-Vc
-Vc
70 kDa
55 kDa
Tubulin (50 kDa)
40 kDa
Total GNAI2 (38 kDa)
35 kDa
25 kDa
15 kDa
SNO-GNAI2
(38 kDa)

## Slide 4
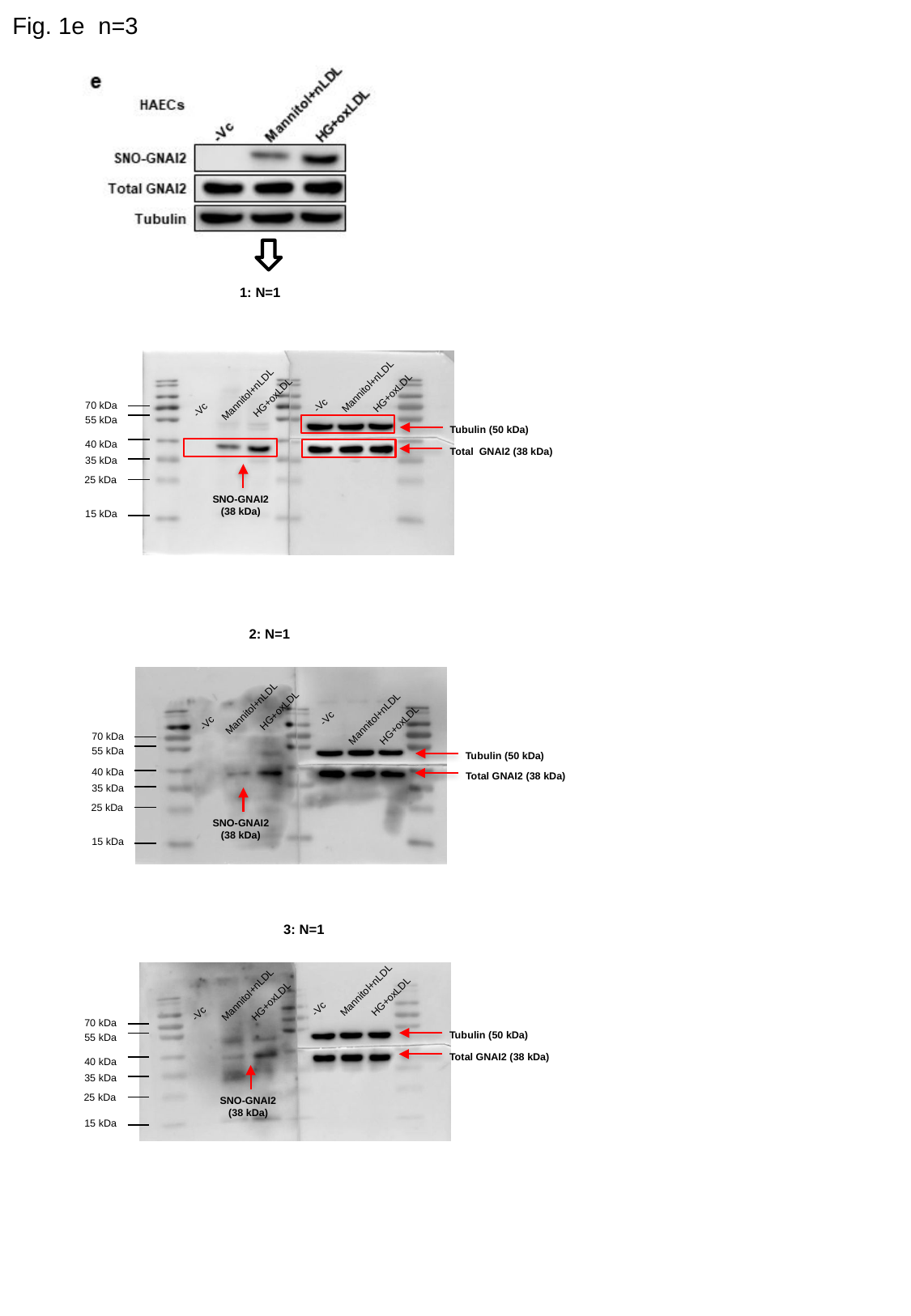

Fig. 1e n=3
1: N=1
Mannitol+nLDL
HG+oxLDL
HG+oxLDL
Mannitol+nLDL
-Vc
-Vc
70 kDa
55 kDa
Tubulin (50 kDa)
40 kDa
Total GNAI2 (38 kDa)
35 kDa
25 kDa
SNO-GNAI2
(38 kDa)
15 kDa
2: N=1
HG+oxLDL
Mannitol+nLDL
Mannitol+nLDL
HG+oxLDL
-Vc
-Vc
70 kDa
55 kDa
Tubulin (50 kDa)
40 kDa
Total GNAI2 (38 kDa)
35 kDa
25 kDa
SNO-GNAI2
(38 kDa)
15 kDa
3: N=1
Mannitol+nLDL
HG+oxLDL
Mannitol+nLDL
HG+oxLDL
-Vc
-Vc
70 kDa
Tubulin (50 kDa)
55 kDa
Total GNAI2 (38 kDa)
40 kDa
35 kDa
25 kDa
SNO-GNAI2
(38 kDa)
15 kDa

## Slide 5
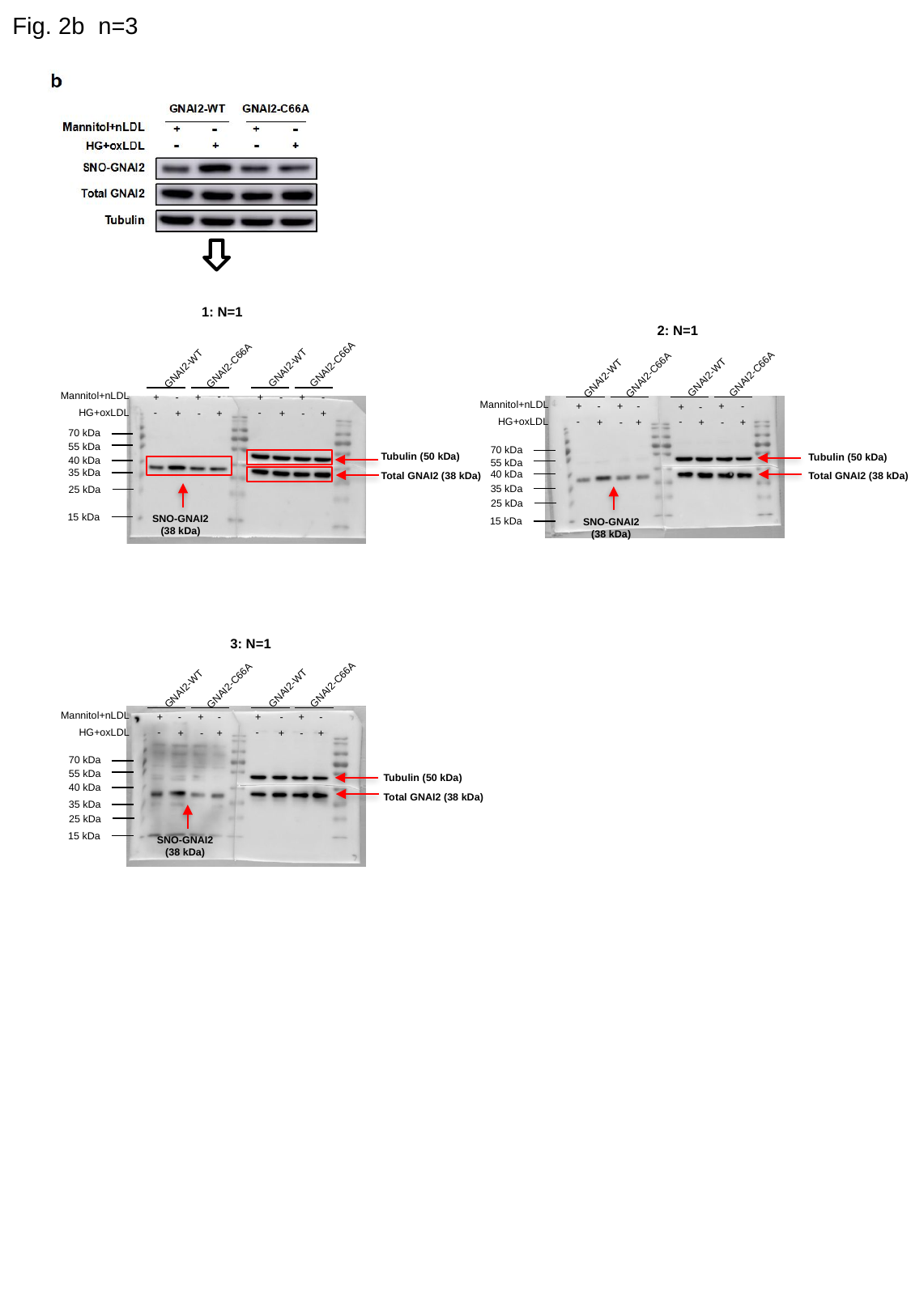

Fig. 2b n=3
1: N=1
2: N=1
GNAI2-C66A
GNAI2-C66A
GNAI2-WT
GNAI2-WT
Mannitol+nLDL
-
+
-
+
+
-
+
-
-
HG+oxLDL
-
-
+
-
+
+
+
70 kDa
55 kDa
Tubulin (50 kDa)
40 kDa
35 kDa
Total GNAI2 (38 kDa)
25 kDa
15 kDa
SNO-GNAI2
(38 kDa)
GNAI2-C66A
GNAI2-C66A
GNAI2-WT
GNAI2-WT
Mannitol+nLDL
-
+
-
+
+
-
+
-
-
HG+oxLDL
-
-
+
-
+
+
+
70 kDa
Tubulin (50 kDa)
55 kDa
40 kDa
Total GNAI2 (38 kDa)
35 kDa
25 kDa
15 kDa
SNO-GNAI2
(38 kDa)
3: N=1
GNAI2-C66A
GNAI2-C66A
GNAI2-WT
GNAI2-WT
Mannitol+nLDL
-
+
-
+
+
-
+
-
-
HG+oxLDL
-
-
+
-
+
+
+
70 kDa
55 kDa
Tubulin (50 kDa)
40 kDa
Total GNAI2 (38 kDa)
35 kDa
25 kDa
15 kDa
SNO-GNAI2
(38 kDa)

## Slide 6
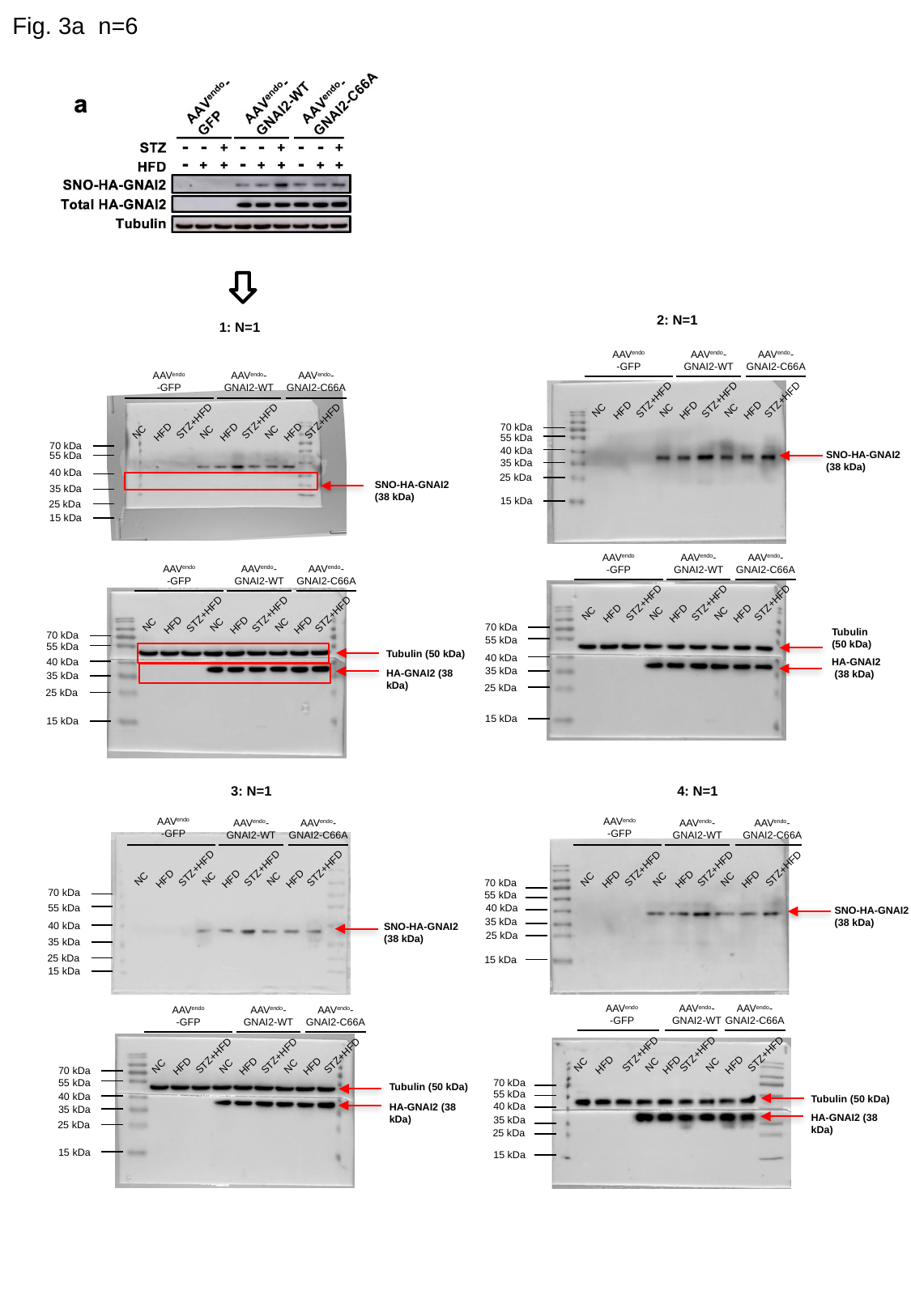

Fig. 3a n=6
2: N=1
1: N=1
AAVendo
-GFP
AAVendo-
GNAI2-WT
AAVendo-
GNAI2-C66A
AAVendo
-GFP
AAVendo-
GNAI2-WT
AAVendo-
GNAI2-C66A
STZ+HFD
STZ+HFD
STZ+HFD
NC
HFD
NC
HFD
NC
HFD
STZ+HFD
STZ+HFD
STZ+HFD
70 kDa
NC
HFD
NC
HFD
NC
HFD
55 kDa
70 kDa
40 kDa
SNO-HA-GNAI2
(38 kDa)
55 kDa
35 kDa
40 kDa
25 kDa
SNO-HA-GNAI2
(38 kDa)
35 kDa
15 kDa
25 kDa
15 kDa
AAVendo
-GFP
AAVendo-
GNAI2-WT
AAVendo-
GNAI2-C66A
AAVendo
-GFP
AAVendo-
GNAI2-WT
AAVendo-
GNAI2-C66A
STZ+HFD
STZ+HFD
STZ+HFD
NC
HFD
NC
HFD
NC
HFD
STZ+HFD
STZ+HFD
STZ+HFD
NC
HFD
NC
HFD
NC
HFD
70 kDa
Tubulin
(50 kDa)
70 kDa
55 kDa
55 kDa
Tubulin (50 kDa)
40 kDa
HA-GNAI2
 (38 kDa)
40 kDa
35 kDa
HA-GNAI2 (38 kDa)
35 kDa
25 kDa
25 kDa
15 kDa
15 kDa
3: N=1
4: N=1
AAVendo
-GFP
AAVendo
-GFP
AAVendo-
GNAI2-WT
AAVendo-
GNAI2-C66A
AAVendo-
GNAI2-WT
AAVendo-
GNAI2-C66A
STZ+HFD
STZ+HFD
STZ+HFD
STZ+HFD
STZ+HFD
STZ+HFD
NC
HFD
NC
HFD
NC
HFD
NC
HFD
NC
HFD
NC
HFD
70 kDa
70 kDa
55 kDa
55 kDa
40 kDa
SNO-HA-GNAI2
(38 kDa)
35 kDa
40 kDa
SNO-HA-GNAI2
(38 kDa)
25 kDa
35 kDa
25 kDa
15 kDa
15 kDa
AAVendo
-GFP
AAVendo-
GNAI2-WT
AAVendo-
GNAI2-C66A
AAVendo
-GFP
AAVendo-
GNAI2-WT
AAVendo-
GNAI2-C66A
STZ+HFD
STZ+HFD
STZ+HFD
STZ+HFD
STZ+HFD
STZ+HFD
NC
HFD
NC
HFD
NC
HFD
NC
HFD
NC
HFD
NC
HFD
70 kDa
55 kDa
70 kDa
Tubulin (50 kDa)
55 kDa
40 kDa
Tubulin (50 kDa)
40 kDa
HA-GNAI2 (38 kDa)
35 kDa
HA-GNAI2 (38 kDa)
35 kDa
25 kDa
25 kDa
15 kDa
15 kDa

## Slide 7
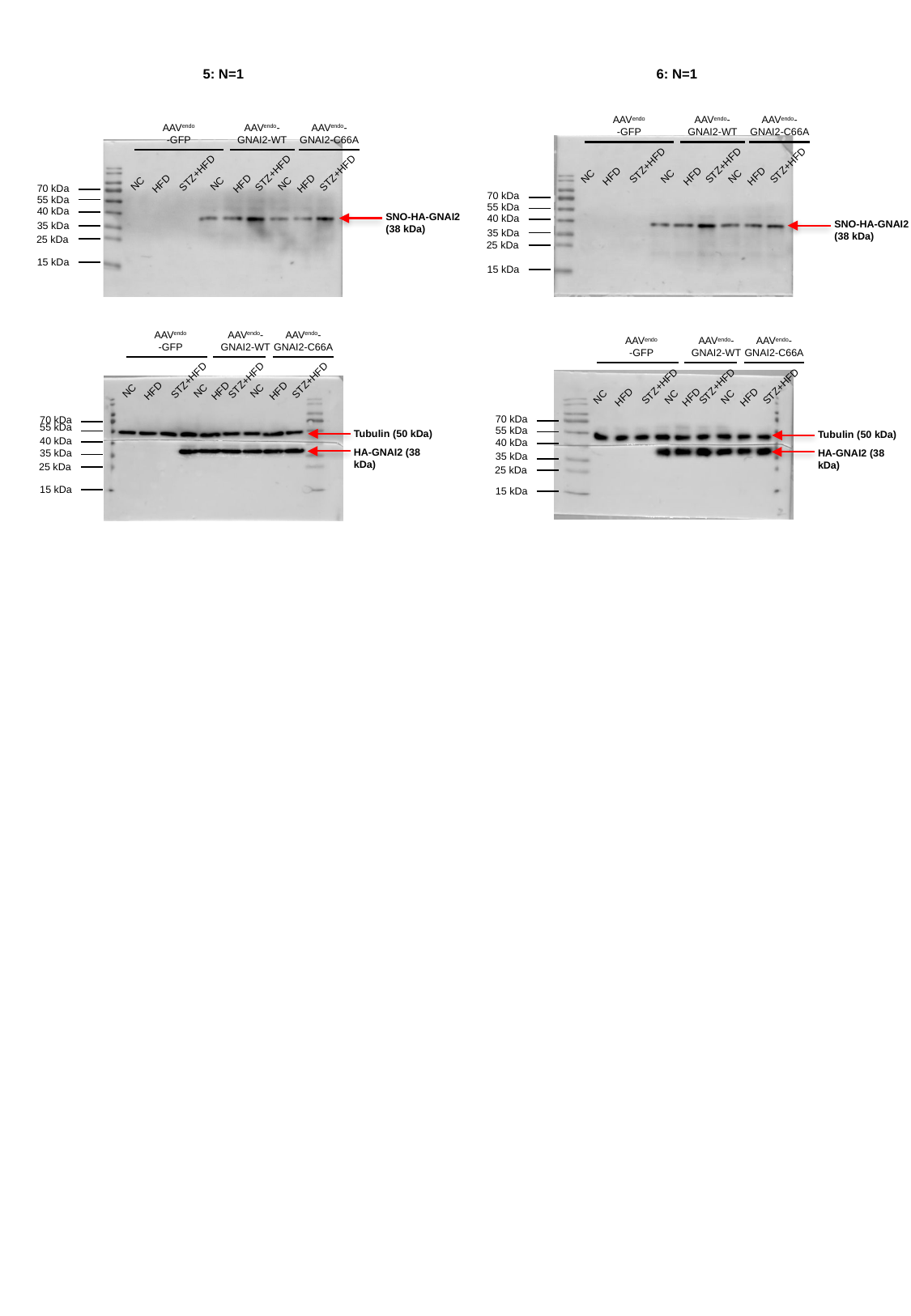

6: N=1
5: N=1
AAVendo
-GFP
AAVendo-
GNAI2-WT
AAVendo-
GNAI2-C66A
AAVendo
-GFP
AAVendo-
GNAI2-WT
AAVendo-
GNAI2-C66A
STZ+HFD
STZ+HFD
STZ+HFD
STZ+HFD
STZ+HFD
STZ+HFD
NC
HFD
NC
HFD
NC
HFD
NC
HFD
NC
HFD
NC
HFD
70 kDa
70 kDa
55 kDa
55 kDa
40 kDa
SNO-HA-GNAI2
(38 kDa)
40 kDa
SNO-HA-GNAI2
(38 kDa)
35 kDa
35 kDa
25 kDa
25 kDa
15 kDa
15 kDa
AAVendo
-GFP
AAVendo-
GNAI2-WT
AAVendo-
GNAI2-C66A
AAVendo
-GFP
AAVendo-
GNAI2-WT
AAVendo-
GNAI2-C66A
STZ+HFD
STZ+HFD
STZ+HFD
STZ+HFD
STZ+HFD
STZ+HFD
NC
HFD
NC
HFD
NC
HFD
NC
HFD
NC
HFD
NC
HFD
70 kDa
70 kDa
55 kDa
55 kDa
Tubulin (50 kDa)
Tubulin (50 kDa)
40 kDa
40 kDa
HA-GNAI2 (38 kDa)
HA-GNAI2 (38 kDa)
35 kDa
35 kDa
25 kDa
25 kDa
15 kDa
15 kDa

## Slide 8
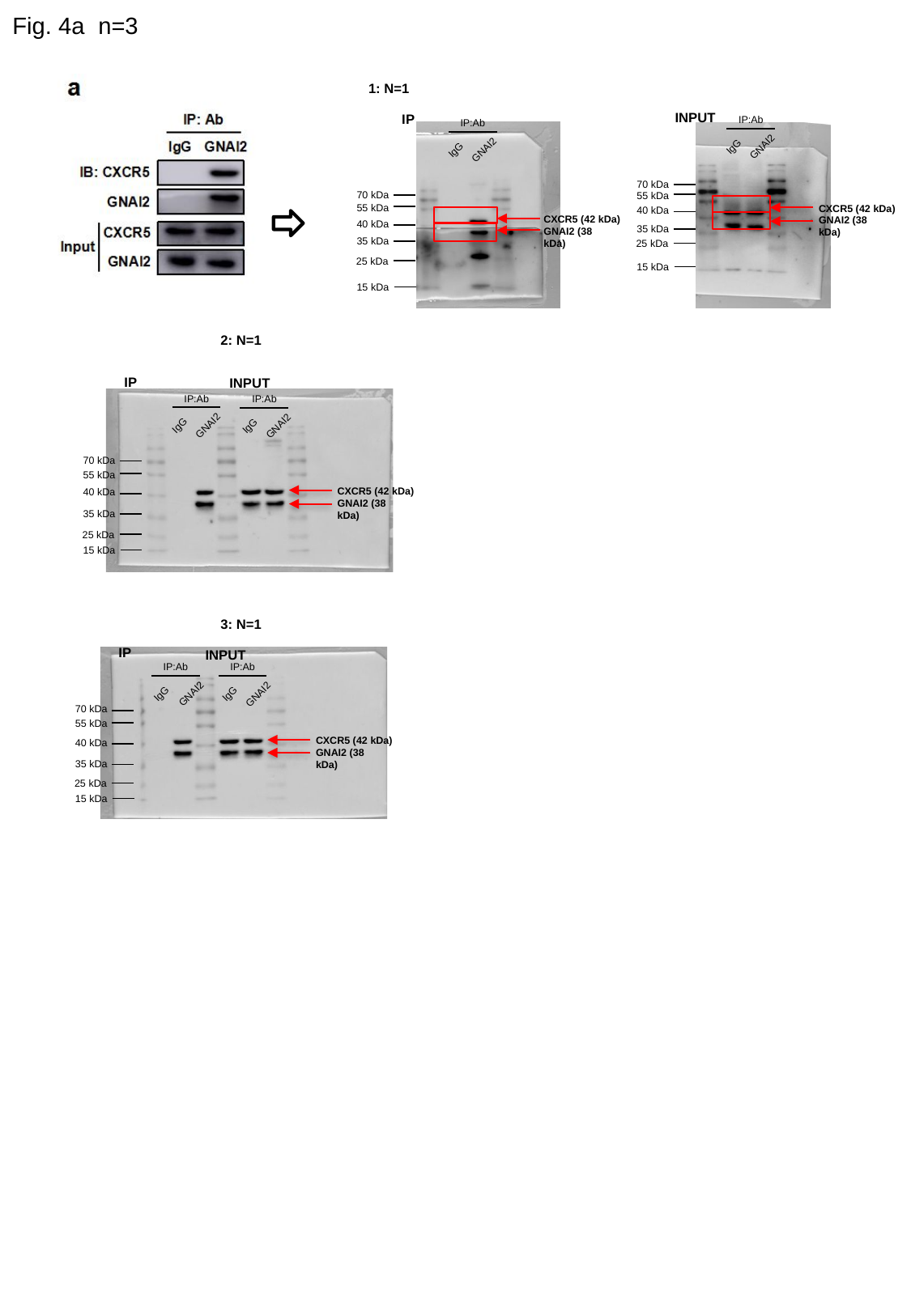

Fig. 4a n=3
1: N=1
INPUT
IP
IP:Ab
IP:Ab
IgG
GNAI2
IgG
GNAI2
70 kDa
70 kDa
55 kDa
55 kDa
CXCR5 (42 kDa)
40 kDa
CXCR5 (42 kDa)
GNAI2 (38 kDa)
40 kDa
35 kDa
GNAI2 (38 kDa)
35 kDa
25 kDa
25 kDa
15 kDa
15 kDa
2: N=1
IP
INPUT
IP:Ab
IP:Ab
IgG
GNAI2
IgG
GNAI2
70 kDa
55 kDa
CXCR5 (42 kDa)
40 kDa
GNAI2 (38 kDa)
35 kDa
25 kDa
15 kDa
3: N=1
IP
INPUT
IP:Ab
IP:Ab
IgG
GNAI2
IgG
GNAI2
70 kDa
55 kDa
CXCR5 (42 kDa)
40 kDa
GNAI2 (38 kDa)
35 kDa
25 kDa
15 kDa

## Slide 9
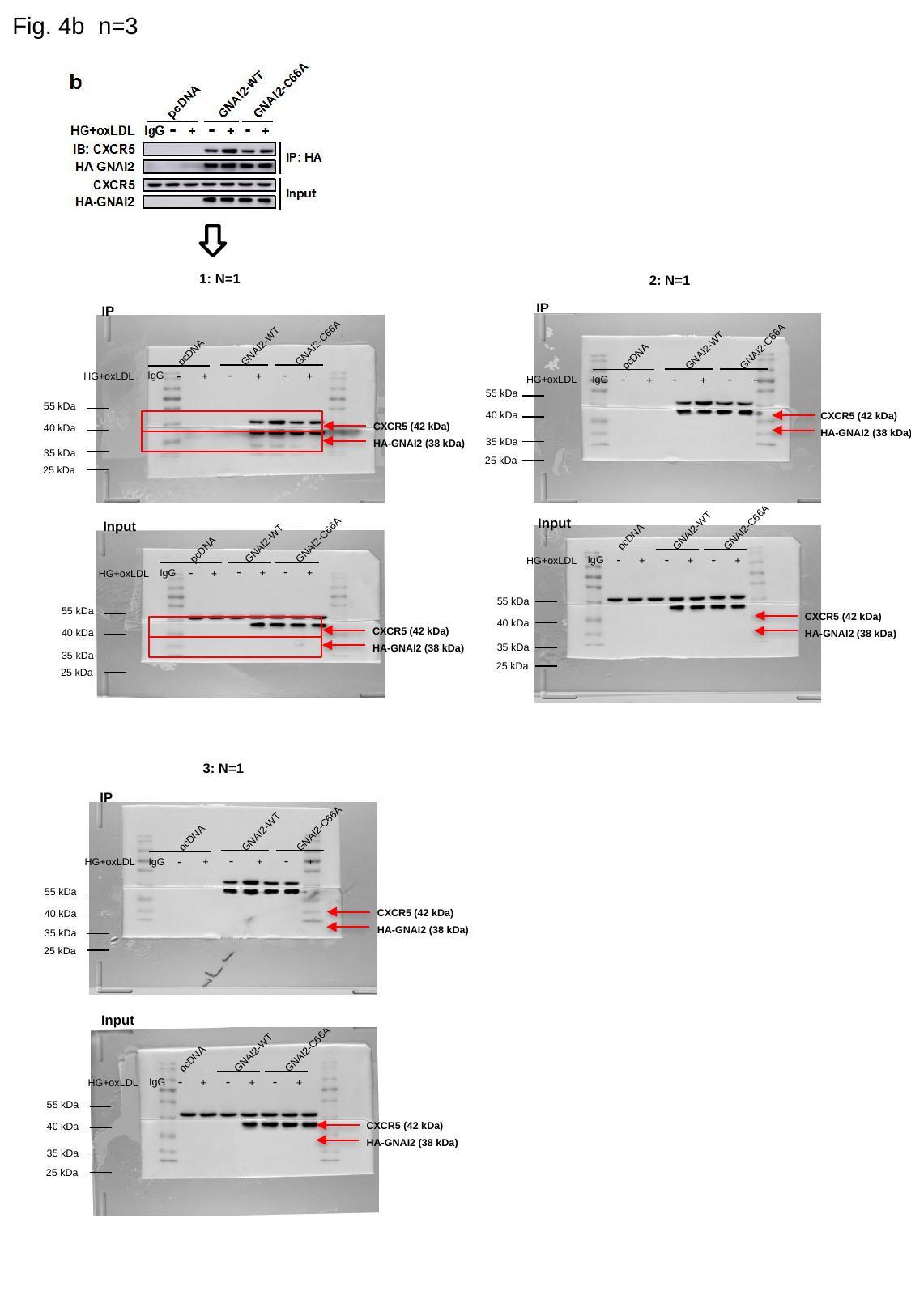

Fig. 4b n=3
1: N=1
2: N=1
IP
IP
GNAI2-C66A
pcDNA
GNAI2-WT
GNAI2-C66A
pcDNA
GNAI2-WT
-
-
-
IgG
+
+
HG+oxLDL
+
-
-
-
HG+oxLDL
IgG
+
+
+
55 kDa
55 kDa
40 kDa
CXCR5 (42 kDa)
CXCR5 (42 kDa)
40 kDa
HA-GNAI2 (38 kDa)
35 kDa
HA-GNAI2 (38 kDa)
35 kDa
25 kDa
25 kDa
pcDNA
GNAI2-WT
Input
Input
GNAI2-C66A
pcDNA
GNAI2-WT
GNAI2-C66A
-
-
-
IgG
+
+
HG+oxLDL
+
-
-
-
IgG
+
+
HG+oxLDL
+
55 kDa
55 kDa
CXCR5 (42 kDa)
40 kDa
CXCR5 (42 kDa)
40 kDa
HA-GNAI2 (38 kDa)
35 kDa
HA-GNAI2 (38 kDa)
35 kDa
25 kDa
25 kDa
3: N=1
IP
GNAI2-C66A
pcDNA
GNAI2-WT
-
-
-
IgG
+
+
HG+oxLDL
+
55 kDa
CXCR5 (42 kDa)
40 kDa
HA-GNAI2 (38 kDa)
35 kDa
25 kDa
Input
pcDNA
GNAI2-WT
GNAI2-C66A
-
-
-
IgG
+
+
HG+oxLDL
+
55 kDa
CXCR5 (42 kDa)
40 kDa
HA-GNAI2 (38 kDa)
35 kDa
25 kDa

## Slide 10
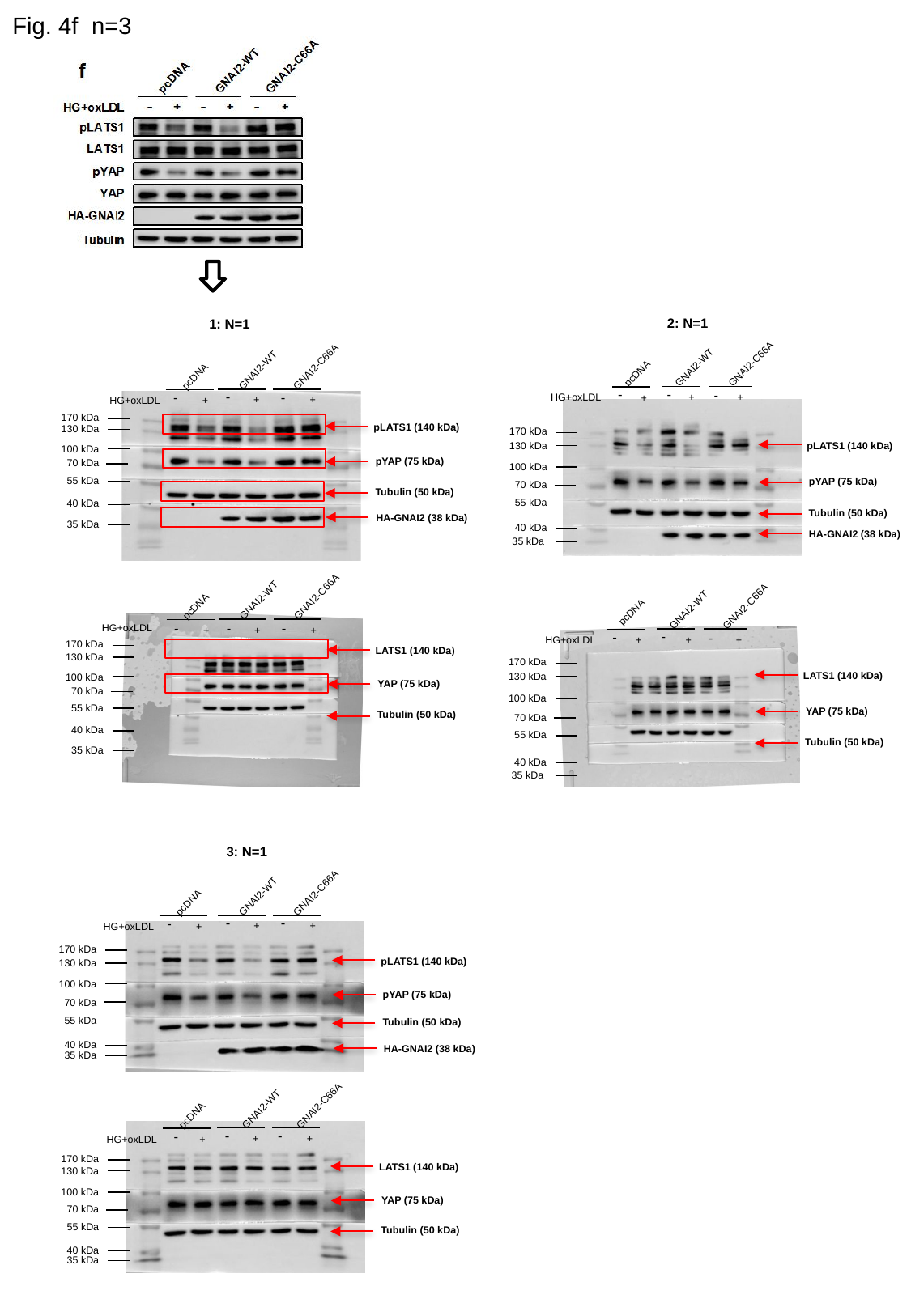

Fig. 4f n=3
2: N=1
1: N=1
GNAI2-C66A
pcDNA
GNAI2-WT
GNAI2-C66A
GNAI2-WT
pcDNA
-
-
-
-
-
-
+
+
HG+oxLDL
+
+
+
HG+oxLDL
+
170 kDa
pLATS1 (140 kDa)
130 kDa
170 kDa
130 kDa
pLATS1 (140 kDa)
100 kDa
pYAP (75 kDa)
70 kDa
100 kDa
55 kDa
pYAP (75 kDa)
70 kDa
Tubulin (50 kDa)
55 kDa
40 kDa
Tubulin (50 kDa)
HA-GNAI2 (38 kDa)
35 kDa
40 kDa
HA-GNAI2 (38 kDa)
35 kDa
GNAI2-C66A
pcDNA
GNAI2-WT
GNAI2-C66A
GNAI2-WT
pcDNA
-
-
-
HG+oxLDL
+
+
+
-
-
-
+
+
HG+oxLDL
+
170 kDa
LATS1 (140 kDa)
130 kDa
170 kDa
LATS1 (140 kDa)
130 kDa
100 kDa
YAP (75 kDa)
70 kDa
100 kDa
55 kDa
YAP (75 kDa)
Tubulin (50 kDa)
70 kDa
40 kDa
55 kDa
Tubulin (50 kDa)
35 kDa
40 kDa
35 kDa
3: N=1
GNAI2-C66A
pcDNA
GNAI2-WT
-
-
-
+
+
HG+oxLDL
+
170 kDa
pLATS1 (140 kDa)
130 kDa
100 kDa
pYAP (75 kDa)
70 kDa
55 kDa
Tubulin (50 kDa)
40 kDa
HA-GNAI2 (38 kDa)
35 kDa
GNAI2-C66A
pcDNA
GNAI2-WT
-
-
-
+
+
HG+oxLDL
+
170 kDa
LATS1 (140 kDa)
130 kDa
100 kDa
YAP (75 kDa)
70 kDa
55 kDa
Tubulin (50 kDa)
40 kDa
35 kDa

## Slide 11
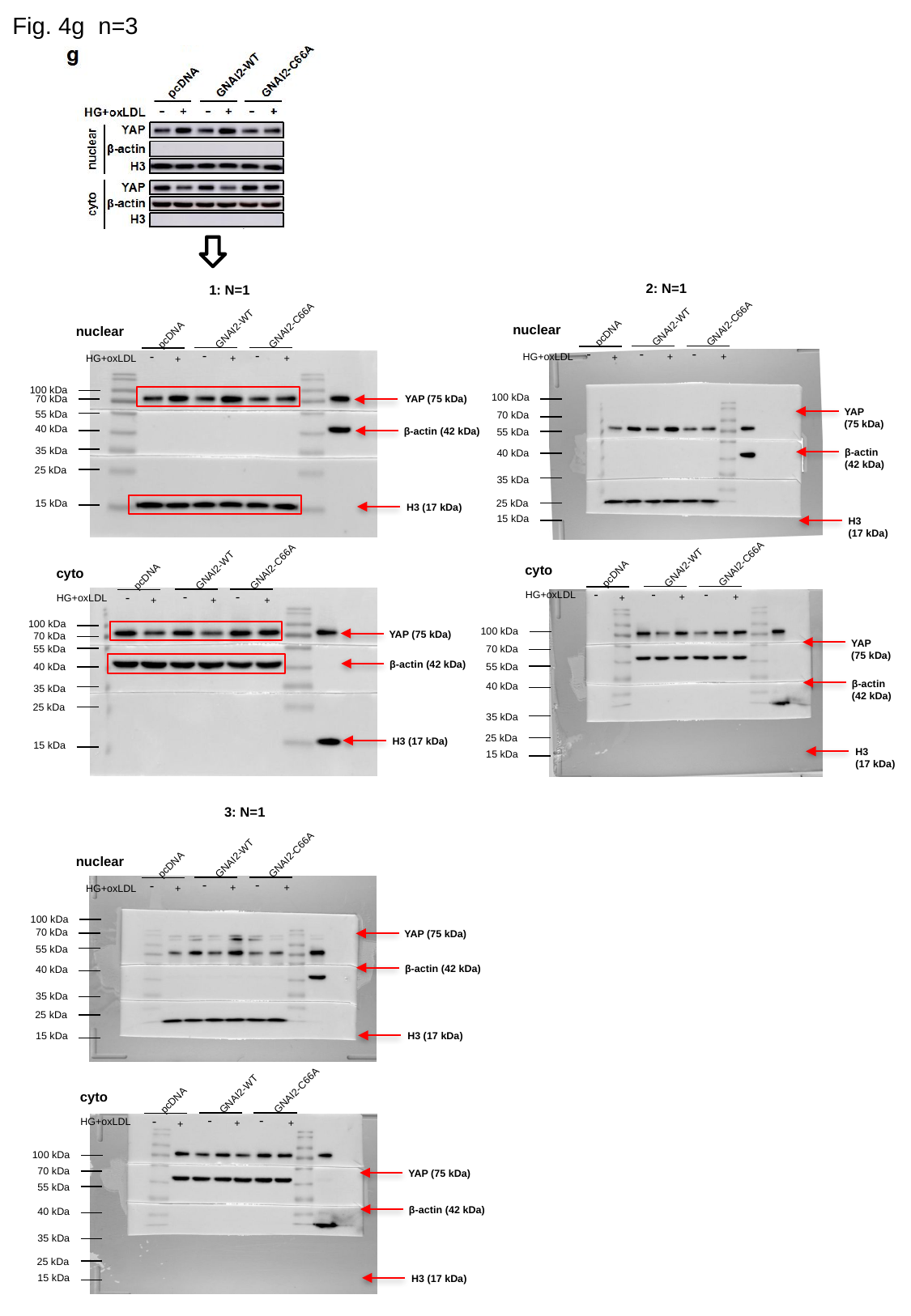

Fig. 4g n=3
2: N=1
1: N=1
GNAI2-C66A
GNAI2-C66A
pcDNA
GNAI2-WT
pcDNA
GNAI2-WT
nuclear
nuclear
-
-
-
-
-
-
+
+
HG+oxLDL
+
+
+
HG+oxLDL
+
100 kDa
100 kDa
70 kDa
YAP (75 kDa)
YAP
(75 kDa)
55 kDa
70 kDa
40 kDa
β-actin (42 kDa)
55 kDa
35 kDa
β-actin
(42 kDa)
40 kDa
25 kDa
35 kDa
15 kDa
25 kDa
H3 (17 kDa)
15 kDa
H3
(17 kDa)
GNAI2-C66A
pcDNA
GNAI2-WT
GNAI2-C66A
pcDNA
GNAI2-WT
cyto
cyto
-
-
-
-
-
HG+oxLDL
-
+
+
+
HG+oxLDL
+
+
+
100 kDa
100 kDa
YAP (75 kDa)
70 kDa
YAP
(75 kDa)
55 kDa
70 kDa
β-actin (42 kDa)
40 kDa
55 kDa
β-actin
(42 kDa)
40 kDa
35 kDa
25 kDa
35 kDa
25 kDa
H3 (17 kDa)
15 kDa
H3
(17 kDa)
15 kDa
3: N=1
GNAI2-C66A
pcDNA
GNAI2-WT
nuclear
-
-
-
+
+
HG+oxLDL
+
100 kDa
70 kDa
YAP (75 kDa)
55 kDa
β-actin (42 kDa)
40 kDa
35 kDa
25 kDa
15 kDa
H3 (17 kDa)
GNAI2-C66A
pcDNA
GNAI2-WT
cyto
-
-
-
HG+oxLDL
+
+
+
100 kDa
70 kDa
YAP (75 kDa)
55 kDa
β-actin (42 kDa)
40 kDa
35 kDa
25 kDa
15 kDa
H3 (17 kDa)

## Slide 12
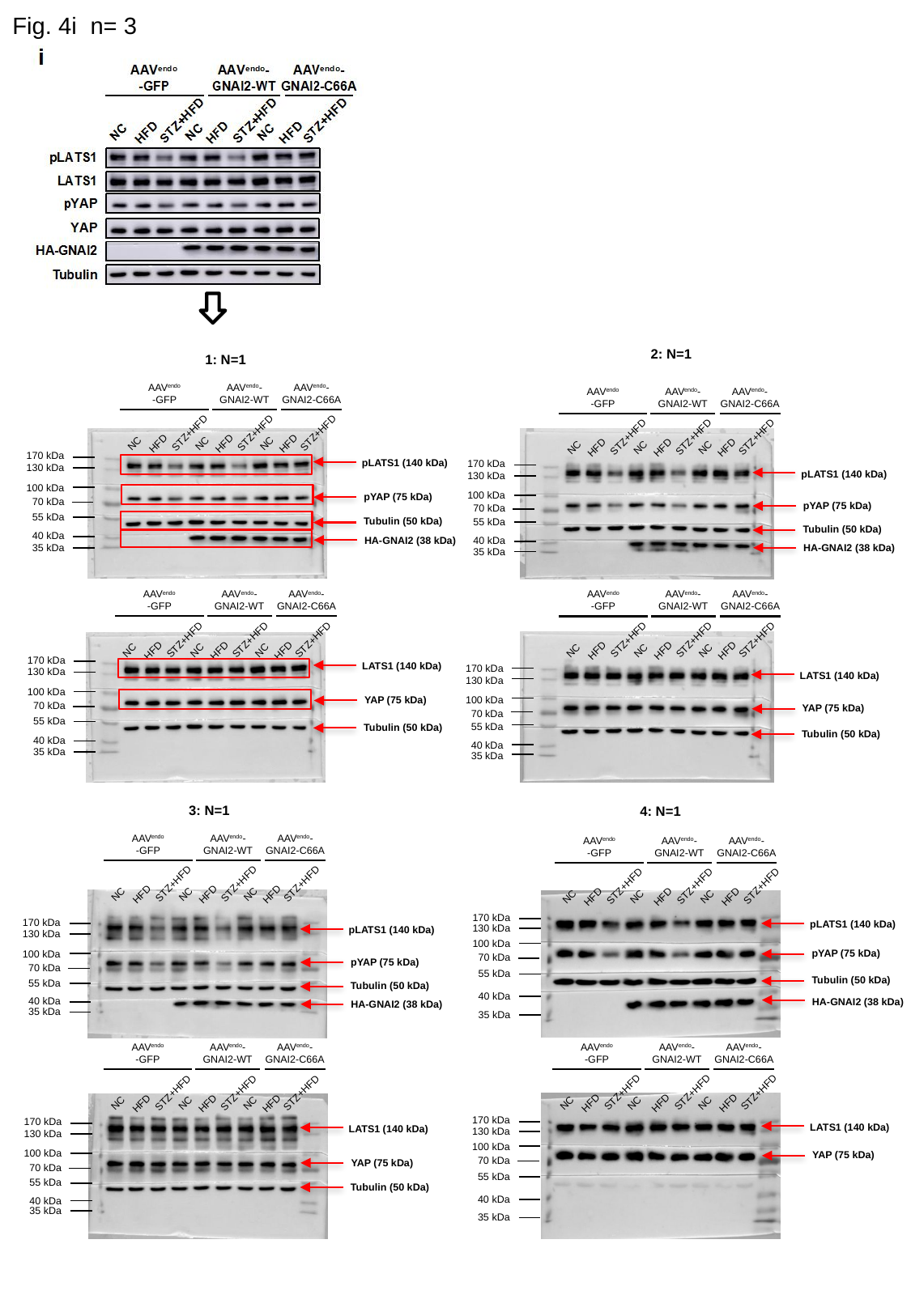

Fig. 4i n= 3
2: N=1
1: N=1
AAVendo
-GFP
AAVendo-
GNAI2-WT
AAVendo-
GNAI2-C66A
AAVendo
-GFP
AAVendo-
GNAI2-WT
AAVendo-
GNAI2-C66A
STZ+HFD
STZ+HFD
STZ+HFD
STZ+HFD
STZ+HFD
STZ+HFD
NC
HFD
NC
HFD
NC
HFD
NC
HFD
NC
HFD
NC
HFD
170 kDa
pLATS1 (140 kDa)
170 kDa
130 kDa
pLATS1 (140 kDa)
130 kDa
100 kDa
100 kDa
pYAP (75 kDa)
70 kDa
pYAP (75 kDa)
70 kDa
55 kDa
Tubulin (50 kDa)
55 kDa
Tubulin (50 kDa)
40 kDa
HA-GNAI2 (38 kDa)
40 kDa
35 kDa
HA-GNAI2 (38 kDa)
35 kDa
AAVendo
-GFP
AAVendo-
GNAI2-WT
AAVendo-
GNAI2-C66A
AAVendo
-GFP
AAVendo-
GNAI2-WT
AAVendo-
GNAI2-C66A
STZ+HFD
STZ+HFD
STZ+HFD
STZ+HFD
STZ+HFD
STZ+HFD
NC
HFD
NC
HFD
NC
HFD
NC
HFD
NC
HFD
NC
HFD
170 kDa
LATS1 (140 kDa)
170 kDa
130 kDa
LATS1 (140 kDa)
130 kDa
100 kDa
YAP (75 kDa)
100 kDa
70 kDa
YAP (75 kDa)
70 kDa
55 kDa
55 kDa
Tubulin (50 kDa)
Tubulin (50 kDa)
40 kDa
40 kDa
35 kDa
35 kDa
3: N=1
4: N=1
AAVendo
-GFP
AAVendo-
GNAI2-WT
AAVendo-
GNAI2-C66A
AAVendo
-GFP
AAVendo-
GNAI2-WT
AAVendo-
GNAI2-C66A
STZ+HFD
STZ+HFD
STZ+HFD
STZ+HFD
STZ+HFD
STZ+HFD
NC
HFD
NC
HFD
NC
HFD
NC
HFD
NC
HFD
NC
HFD
170 kDa
170 kDa
pLATS1 (140 kDa)
130 kDa
pLATS1 (140 kDa)
130 kDa
100 kDa
pYAP (75 kDa)
100 kDa
70 kDa
pYAP (75 kDa)
70 kDa
55 kDa
Tubulin (50 kDa)
55 kDa
Tubulin (50 kDa)
40 kDa
40 kDa
HA-GNAI2 (38 kDa)
HA-GNAI2 (38 kDa)
35 kDa
35 kDa
AAVendo
-GFP
AAVendo-
GNAI2-WT
AAVendo-
GNAI2-C66A
AAVendo
-GFP
AAVendo-
GNAI2-WT
AAVendo-
GNAI2-C66A
STZ+HFD
STZ+HFD
STZ+HFD
STZ+HFD
STZ+HFD
STZ+HFD
NC
HFD
NC
HFD
NC
HFD
NC
HFD
NC
HFD
NC
HFD
170 kDa
170 kDa
LATS1 (140 kDa)
LATS1 (140 kDa)
130 kDa
130 kDa
100 kDa
100 kDa
YAP (75 kDa)
70 kDa
YAP (75 kDa)
70 kDa
55 kDa
55 kDa
Tubulin (50 kDa)
40 kDa
40 kDa
35 kDa
35 kDa

## Slide 13
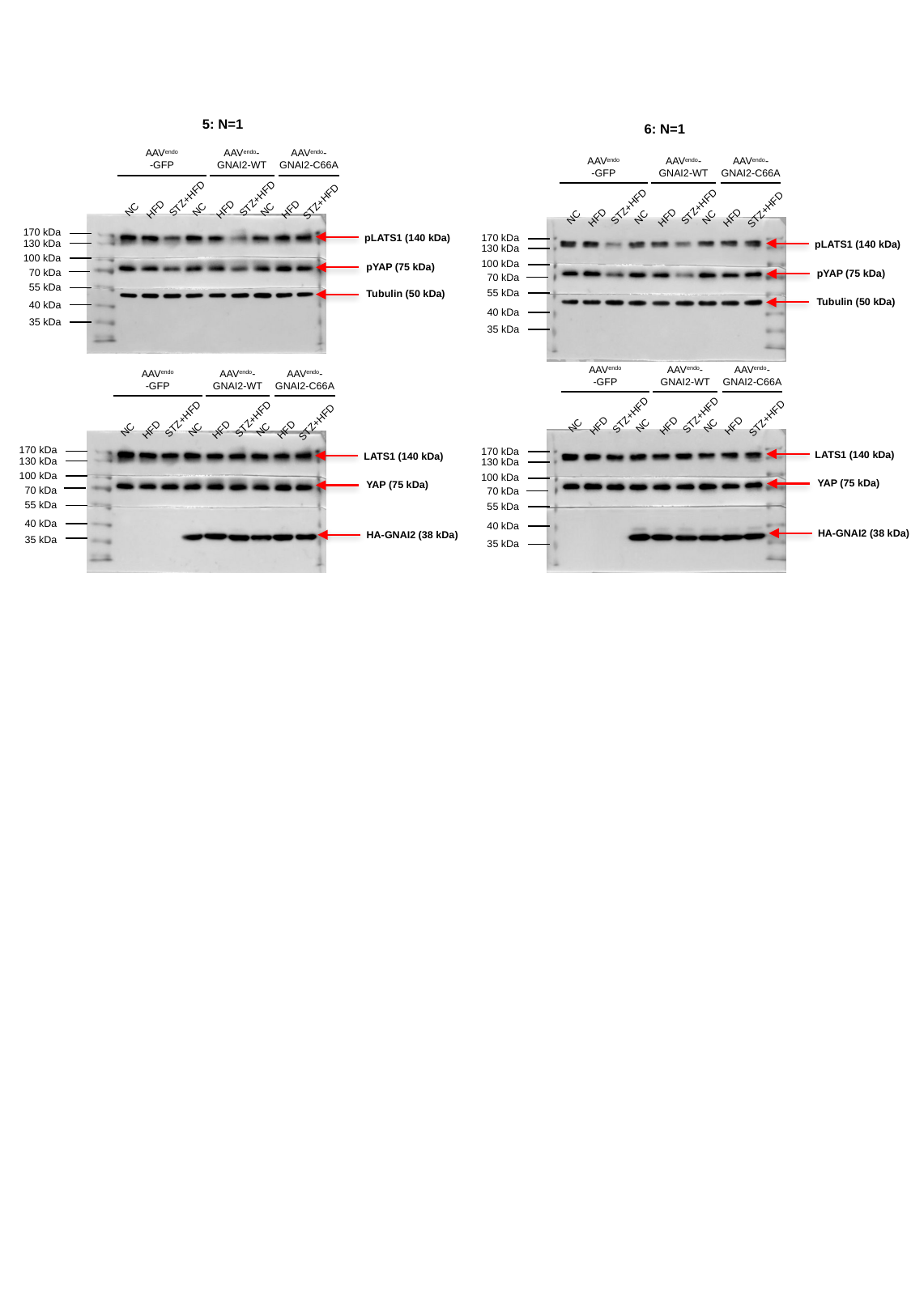

5: N=1
6: N=1
AAVendo
-GFP
AAVendo-
GNAI2-WT
AAVendo-
GNAI2-C66A
AAVendo
-GFP
AAVendo-
GNAI2-WT
AAVendo-
GNAI2-C66A
STZ+HFD
STZ+HFD
STZ+HFD
STZ+HFD
STZ+HFD
NC
HFD
NC
HFD
NC
HFD
STZ+HFD
NC
HFD
NC
HFD
NC
HFD
170 kDa
170 kDa
pLATS1 (140 kDa)
130 kDa
pLATS1 (140 kDa)
130 kDa
100 kDa
100 kDa
pYAP (75 kDa)
70 kDa
pYAP (75 kDa)
70 kDa
55 kDa
55 kDa
Tubulin (50 kDa)
Tubulin (50 kDa)
40 kDa
40 kDa
35 kDa
35 kDa
AAVendo
-GFP
AAVendo-
GNAI2-WT
AAVendo-
GNAI2-C66A
AAVendo
-GFP
AAVendo-
GNAI2-WT
AAVendo-
GNAI2-C66A
STZ+HFD
STZ+HFD
STZ+HFD
STZ+HFD
STZ+HFD
STZ+HFD
NC
HFD
NC
HFD
NC
HFD
NC
HFD
NC
HFD
NC
HFD
170 kDa
170 kDa
LATS1 (140 kDa)
LATS1 (140 kDa)
130 kDa
130 kDa
100 kDa
100 kDa
YAP (75 kDa)
YAP (75 kDa)
70 kDa
70 kDa
55 kDa
55 kDa
40 kDa
40 kDa
HA-GNAI2 (38 kDa)
HA-GNAI2 (38 kDa)
35 kDa
35 kDa

## Slide 14
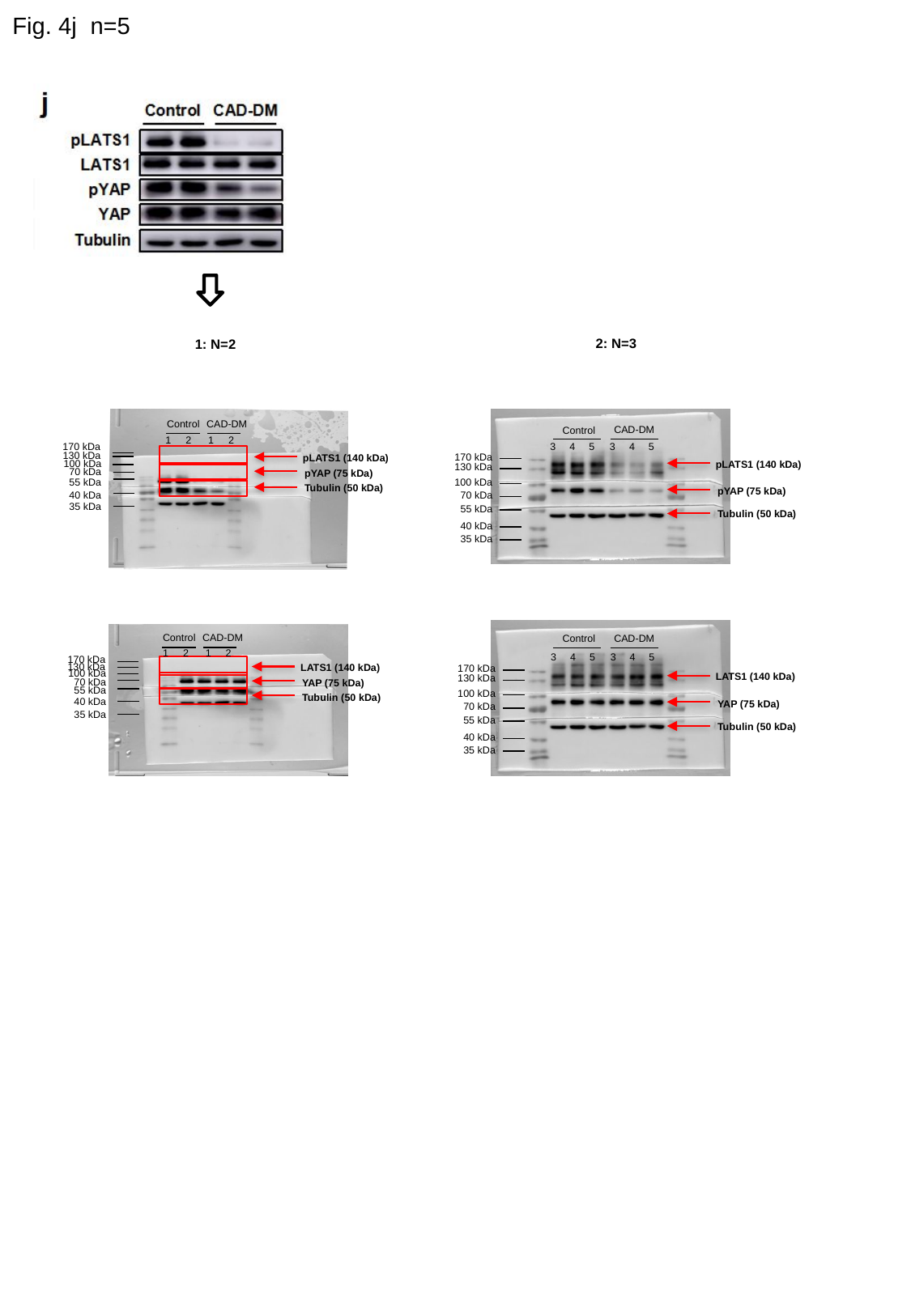

Fig. 4j n=5
2: N=3
1: N=2
Control
CAD-DM
CAD-DM
Control
1
2
1
2
3
4
5
3
4
5
170 kDa
130 kDa
170 kDa
pLATS1 (140 kDa)
100 kDa
pLATS1 (140 kDa)
130 kDa
70 kDa
pYAP (75 kDa)
100 kDa
55 kDa
Tubulin (50 kDa)
pYAP (75 kDa)
40 kDa
70 kDa
35 kDa
55 kDa
Tubulin (50 kDa)
40 kDa
35 kDa
Control
CAD-DM
Control
CAD-DM
1
2
1
2
3
4
5
3
4
5
170 kDa
130 kDa
LATS1 (140 kDa)
170 kDa
100 kDa
LATS1 (140 kDa)
130 kDa
70 kDa
YAP (75 kDa)
55 kDa
100 kDa
Tubulin (50 kDa)
40 kDa
YAP (75 kDa)
70 kDa
35 kDa
55 kDa
Tubulin (50 kDa)
40 kDa
35 kDa

## Slide 15
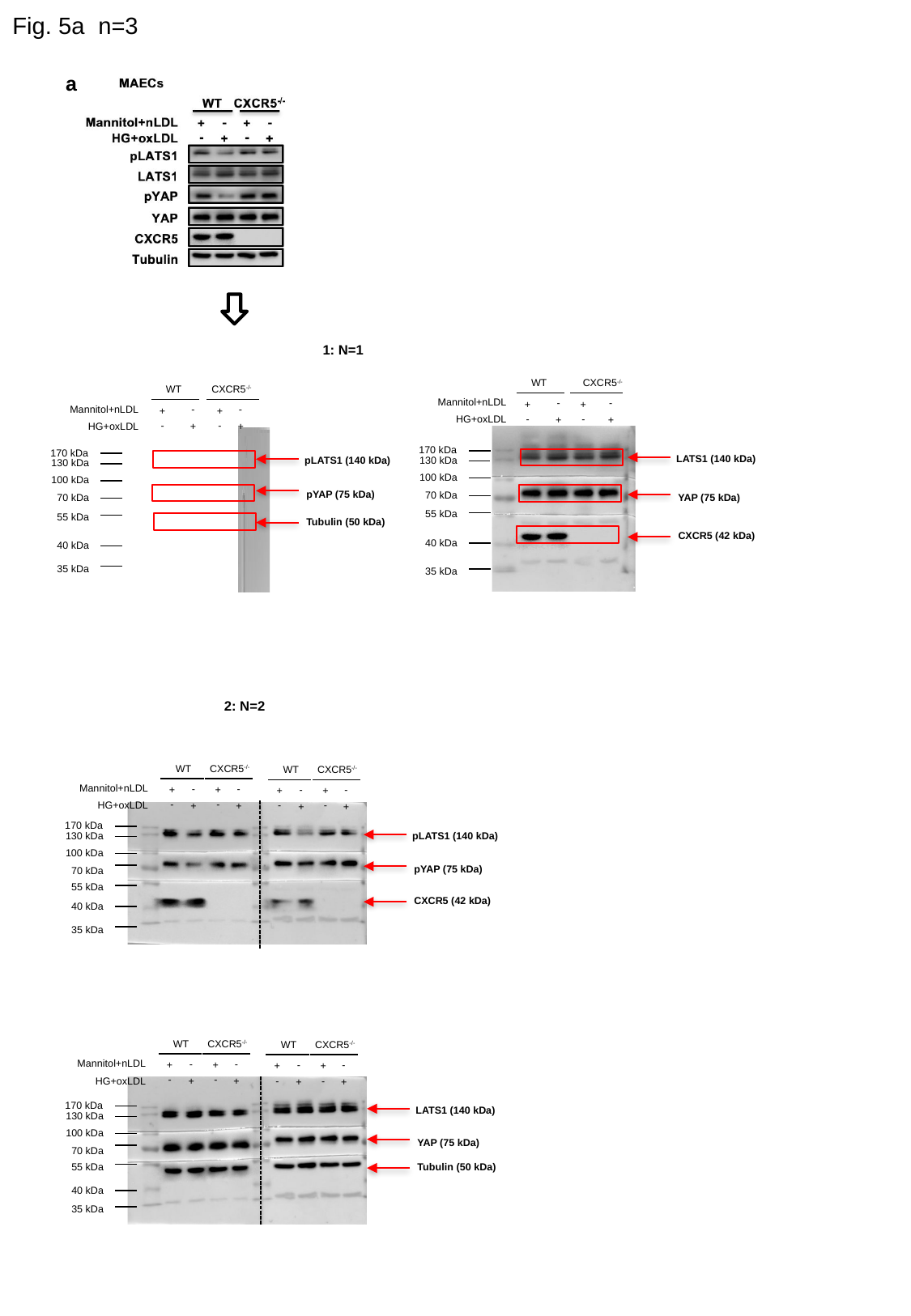

Fig. 5a n=3
a
1: N=1
WT
CXCR5-/-
WT
CXCR5-/-
Mannitol+nLDL
-
-
+
+
Mannitol+nLDL
-
-
+
+
-
-
HG+oxLDL
+
+
-
-
HG+oxLDL
+
+
170 kDa
170 kDa
LATS1 (140 kDa)
130 kDa
pLATS1 (140 kDa)
130 kDa
100 kDa
100 kDa
pYAP (75 kDa)
70 kDa
70 kDa
YAP (75 kDa)
55 kDa
55 kDa
Tubulin (50 kDa)
CXCR5 (42 kDa)
40 kDa
40 kDa
35 kDa
35 kDa
2: N=2
WT
CXCR5-/-
WT
CXCR5-/-
Mannitol+nLDL
-
-
-
-
+
+
+
+
-
-
-
-
HG+oxLDL
+
+
+
+
170 kDa
130 kDa
pLATS1 (140 kDa)
100 kDa
pYAP (75 kDa)
70 kDa
55 kDa
CXCR5 (42 kDa)
40 kDa
35 kDa
WT
CXCR5-/-
WT
CXCR5-/-
Mannitol+nLDL
-
-
-
-
+
+
+
+
-
-
-
-
HG+oxLDL
+
+
+
+
170 kDa
LATS1 (140 kDa)
130 kDa
100 kDa
YAP (75 kDa)
70 kDa
55 kDa
Tubulin (50 kDa)
40 kDa
35 kDa

## Slide 16
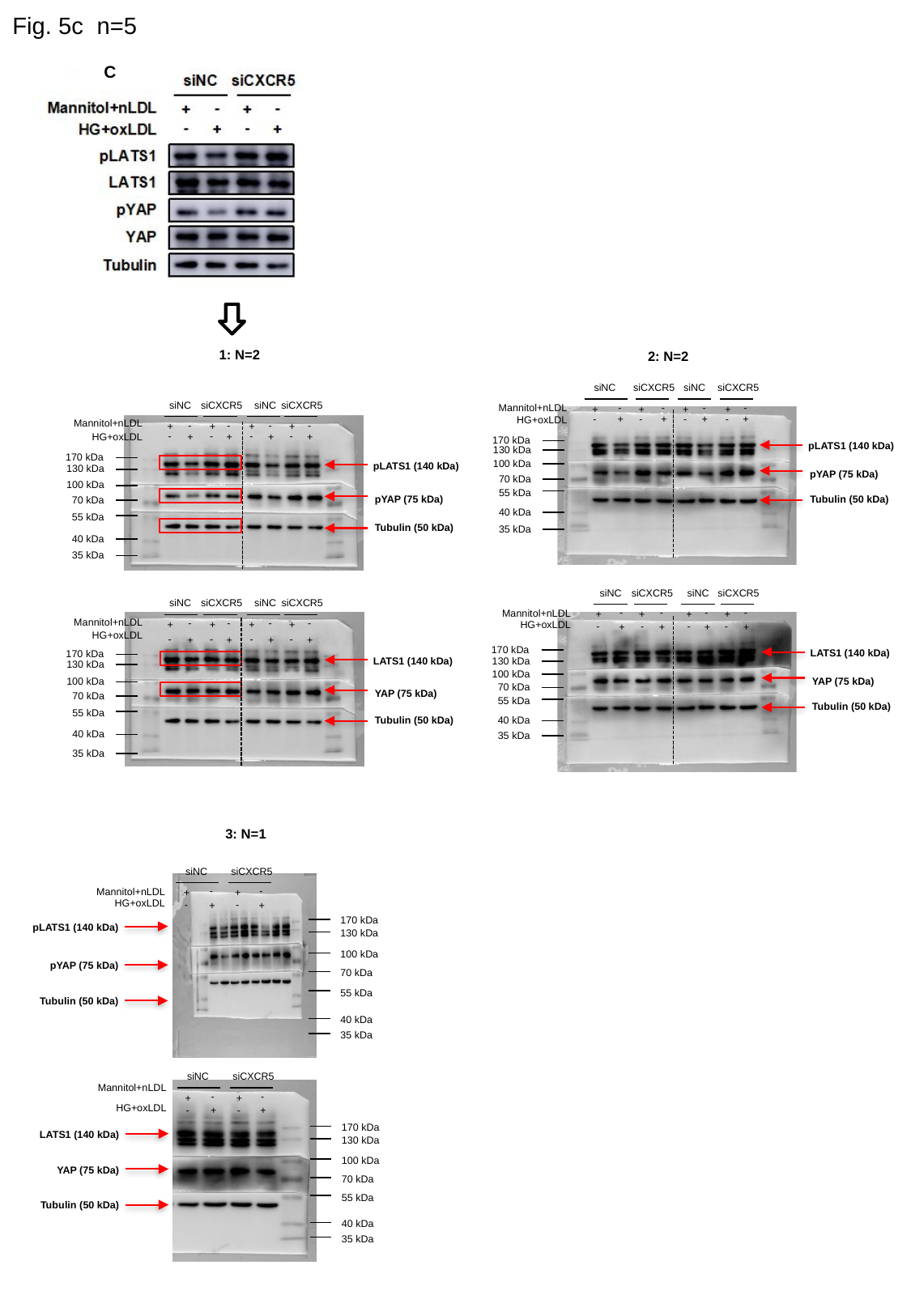

Fig. 5c n=5
C
1: N=2
2: N=2
siNC
siCXCR5
siNC
siCXCR5
siNC
siCXCR5
siNC
siCXCR5
-
-
-
-
Mannitol+nLDL
+
+
+
+
-
-
-
-
+
+
+
+
HG+oxLDL
Mannitol+nLDL
-
-
-
-
+
+
+
+
-
-
-
-
+
+
+
+
HG+oxLDL
170 kDa
pLATS1 (140 kDa)
130 kDa
170 kDa
100 kDa
pLATS1 (140 kDa)
130 kDa
pYAP (75 kDa)
70 kDa
100 kDa
55 kDa
Tubulin (50 kDa)
pYAP (75 kDa)
70 kDa
40 kDa
55 kDa
Tubulin (50 kDa)
35 kDa
40 kDa
35 kDa
siNC
siCXCR5
siNC
siCXCR5
siNC
siCXCR5
siNC
siCXCR5
-
-
-
-
Mannitol+nLDL
+
+
+
+
Mannitol+nLDL
-
-
-
-
+
+
+
+
HG+oxLDL
-
-
-
-
+
+
+
+
HG+oxLDL
-
-
-
-
+
+
+
+
170 kDa
LATS1 (140 kDa)
170 kDa
LATS1 (140 kDa)
130 kDa
130 kDa
100 kDa
YAP (75 kDa)
100 kDa
70 kDa
YAP (75 kDa)
70 kDa
55 kDa
Tubulin (50 kDa)
55 kDa
Tubulin (50 kDa)
40 kDa
40 kDa
35 kDa
35 kDa
3: N=1
siNC
siCXCR5
-
-
Mannitol+nLDL
+
+
HG+oxLDL
-
-
+
+
170 kDa
pLATS1 (140 kDa)
130 kDa
100 kDa
pYAP (75 kDa)
70 kDa
55 kDa
Tubulin (50 kDa)
40 kDa
35 kDa
siNC
siCXCR5
Mannitol+nLDL
-
-
+
+
HG+oxLDL
-
-
+
+
170 kDa
LATS1 (140 kDa)
130 kDa
100 kDa
YAP (75 kDa)
70 kDa
55 kDa
Tubulin (50 kDa)
40 kDa
35 kDa

## Slide 17
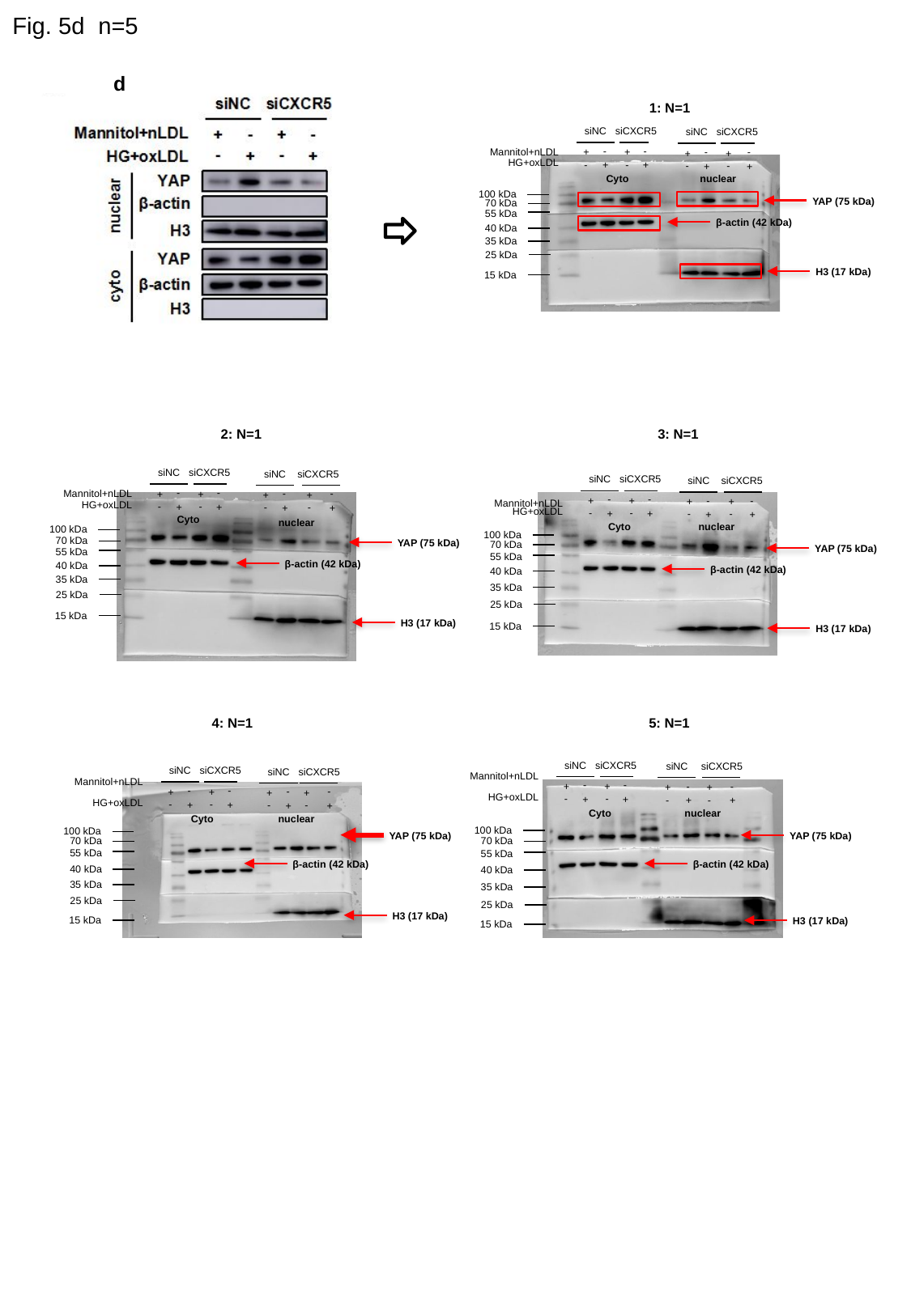

Fig. 5d n=5
d
1: N=1
siNC
siCXCR5
siNC
siCXCR5
-
-
-
-
Mannitol+nLDL
+
+
+
+
HG+oxLDL
-
-
+
+
-
-
+
+
Cyto
nuclear
100 kDa
YAP (75 kDa)
70 kDa
55 kDa
β-actin (42 kDa)
40 kDa
35 kDa
25 kDa
H3 (17 kDa)
15 kDa
2: N=1
3: N=1
siNC
siCXCR5
siNC
siCXCR5
siNC
siCXCR5
siNC
siCXCR5
-
-
Mannitol+nLDL
-
-
+
+
+
+
-
-
-
-
+
+
+
+
Mannitol+nLDL
HG+oxLDL
-
-
+
+
-
-
+
+
HG+oxLDL
-
-
+
+
-
-
+
+
Cyto
nuclear
Cyto
nuclear
100 kDa
100 kDa
70 kDa
YAP (75 kDa)
70 kDa
YAP (75 kDa)
55 kDa
55 kDa
β-actin (42 kDa)
40 kDa
β-actin (42 kDa)
40 kDa
35 kDa
35 kDa
25 kDa
25 kDa
15 kDa
H3 (17 kDa)
15 kDa
H3 (17 kDa)
4: N=1
5: N=1
siNC
siCXCR5
siNC
siCXCR5
siNC
siCXCR5
siNC
siCXCR5
Mannitol+nLDL
Mannitol+nLDL
-
-
-
-
+
+
+
+
-
-
-
-
+
+
+
+
HG+oxLDL
-
-
+
+
-
-
+
+
HG+oxLDL
-
-
+
+
-
-
+
+
Cyto
nuclear
Cyto
nuclear
100 kDa
100 kDa
YAP (75 kDa)
YAP (75 kDa)
70 kDa
70 kDa
55 kDa
55 kDa
β-actin (42 kDa)
β-actin (42 kDa)
40 kDa
40 kDa
35 kDa
35 kDa
25 kDa
25 kDa
H3 (17 kDa)
15 kDa
H3 (17 kDa)
15 kDa

## Slide 18
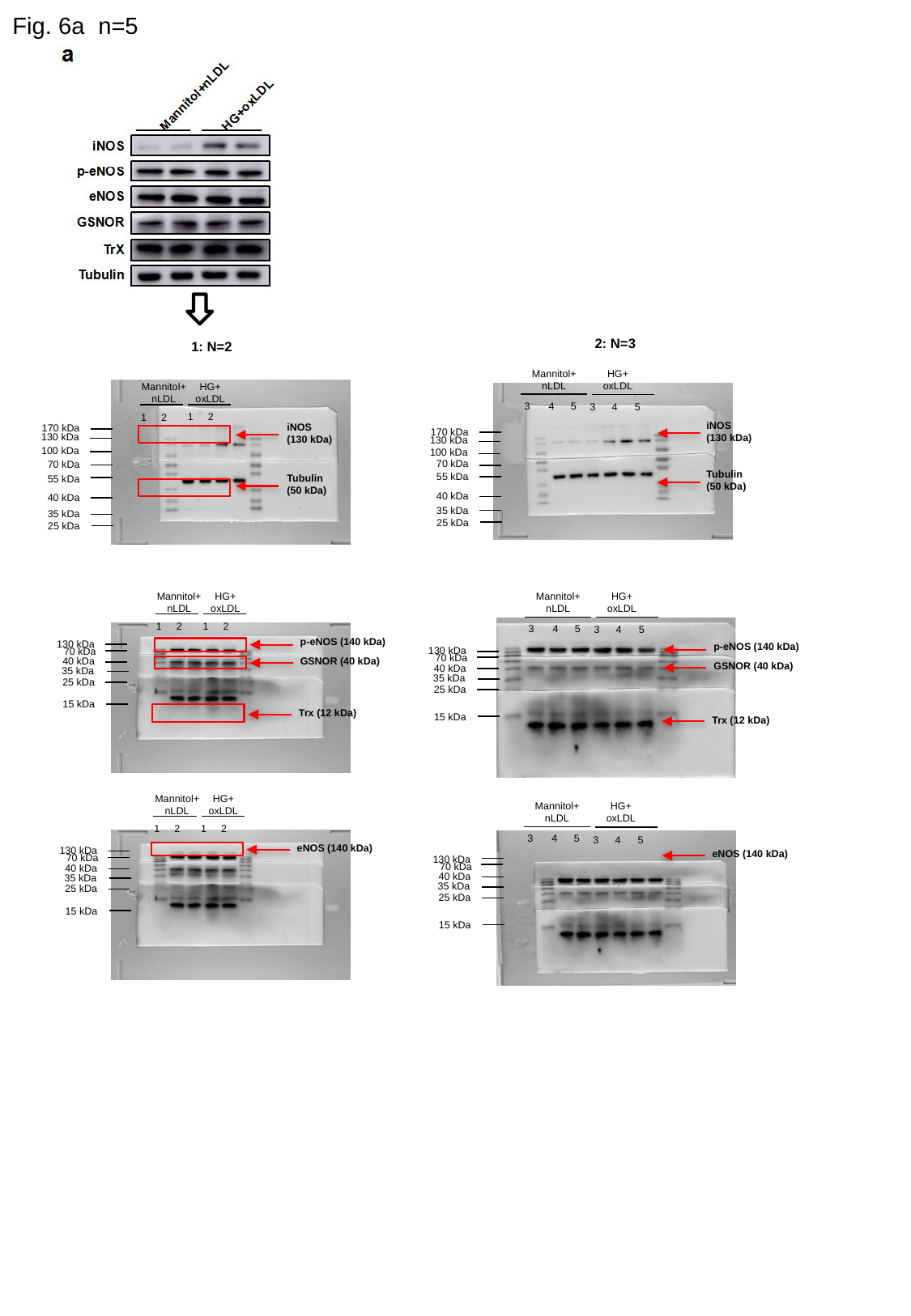

Fig. 6a n=5
2: N=3
1: N=2
Mannitol+
nLDL
HG+
oxLDL
Mannitol+
nLDL
HG+
oxLDL
3
4
5
3
4
5
1
2
1
2
iNOS
(130 kDa)
iNOS
(130 kDa)
170 kDa
170 kDa
130 kDa
130 kDa
100 kDa
100 kDa
70 kDa
70 kDa
Tubulin
(50 kDa)
55 kDa
Tubulin
(50 kDa)
55 kDa
40 kDa
40 kDa
35 kDa
35 kDa
25 kDa
25 kDa
Mannitol+
nLDL
HG+
oxLDL
Mannitol+
nLDL
HG+
oxLDL
1
2
1
2
3
4
5
3
4
5
p-eNOS (140 kDa)
130 kDa
p-eNOS (140 kDa)
130 kDa
70 kDa
70 kDa
40 kDa
GSNOR (40 kDa)
GSNOR (40 kDa)
40 kDa
35 kDa
35 kDa
25 kDa
25 kDa
15 kDa
Trx (12 kDa)
15 kDa
Trx (12 kDa)
Mannitol+
nLDL
HG+
oxLDL
Mannitol+
nLDL
HG+
oxLDL
1
2
1
2
3
4
5
3
4
5
eNOS (140 kDa)
130 kDa
eNOS (140 kDa)
70 kDa
130 kDa
70 kDa
40 kDa
40 kDa
35 kDa
35 kDa
25 kDa
25 kDa
15 kDa
15 kDa

## Slide 19
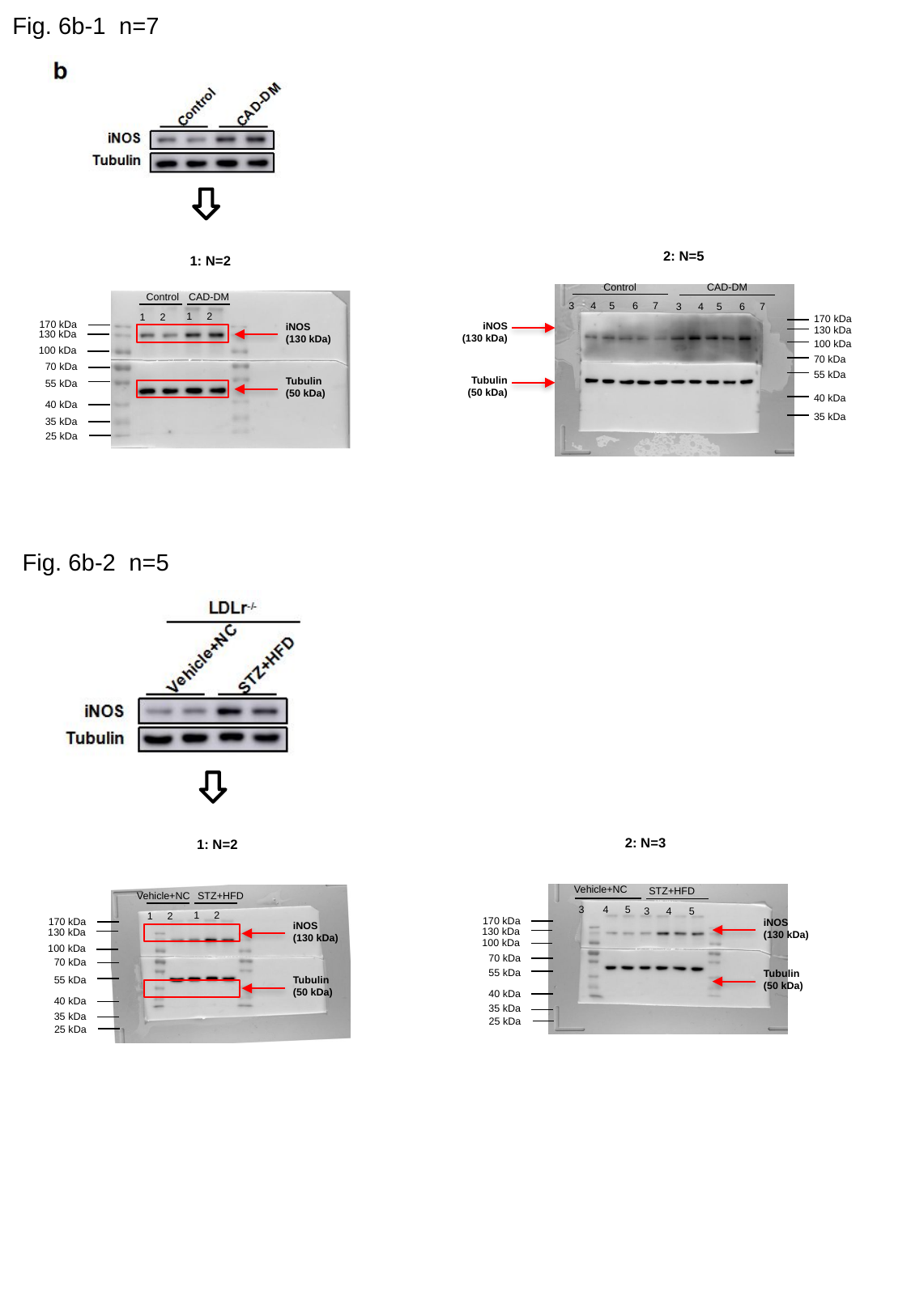

Fig. 6b-1 n=7
2: N=5
1: N=2
Control
CAD-DM
Control
CAD-DM
3
4
5
6
7
6
7
3
4
5
1
2
1
2
170 kDa
170 kDa
iNOS
(130 kDa)
iNOS
(130 kDa)
130 kDa
130 kDa
100 kDa
100 kDa
70 kDa
70 kDa
55 kDa
Tubulin
(50 kDa)
Tubulin
(50 kDa)
55 kDa
40 kDa
40 kDa
35 kDa
35 kDa
25 kDa
Fig. 6b-2 n=5
2: N=3
1: N=2
Vehicle+NC
STZ+HFD
Vehicle+NC
STZ+HFD
3
4
5
3
4
5
1
2
1
2
170 kDa
170 kDa
iNOS
(130 kDa)
iNOS
(130 kDa)
130 kDa
130 kDa
100 kDa
100 kDa
70 kDa
70 kDa
55 kDa
Tubulin
(50 kDa)
55 kDa
Tubulin
(50 kDa)
40 kDa
40 kDa
35 kDa
35 kDa
25 kDa
25 kDa

## Slide 20
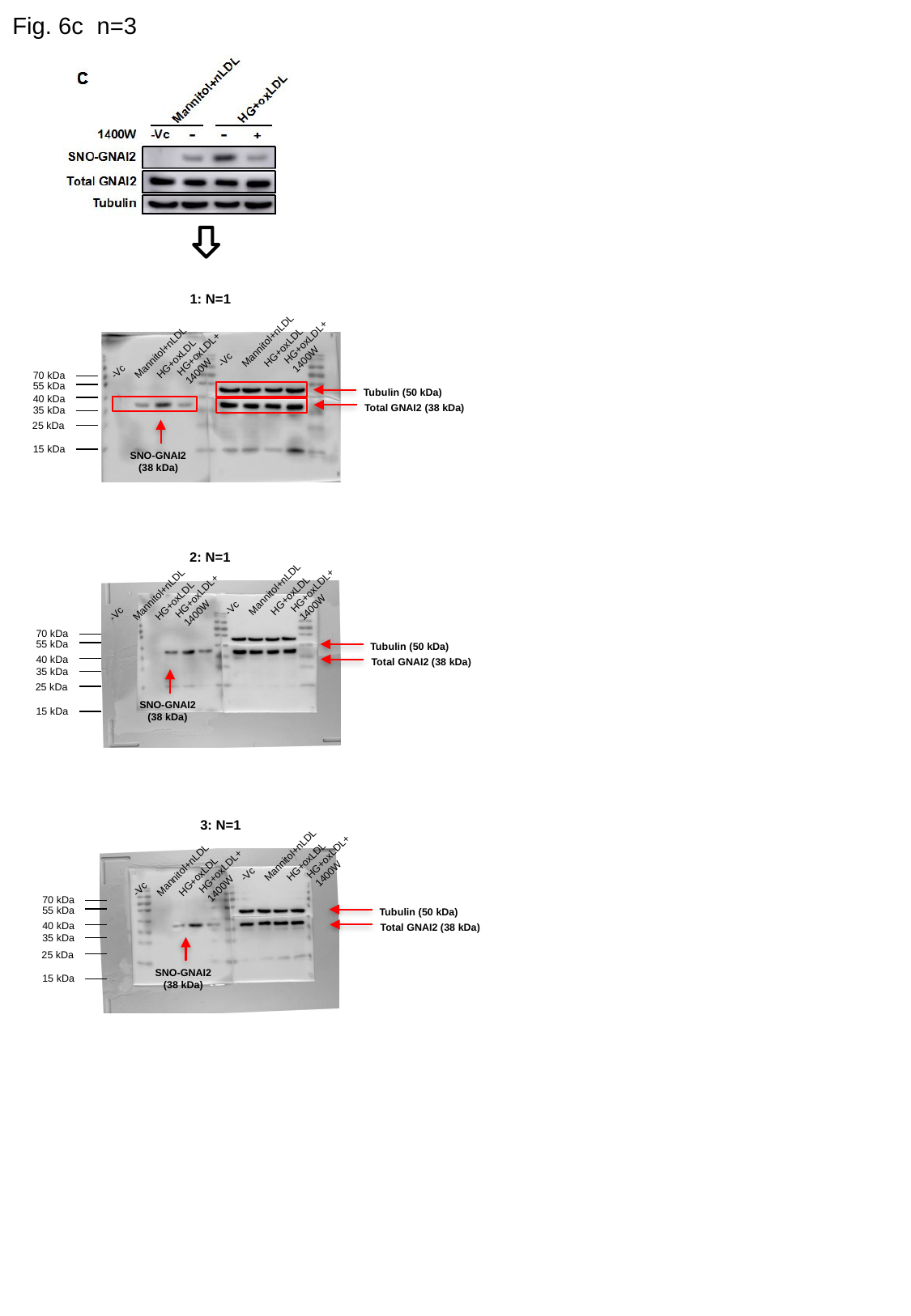

Fig. 6c n=3
1: N=1
Mannitol+nLDL
HG+oxLDL+1400W
HG+oxLDL
-Vc
Mannitol+nLDL
HG+oxLDL+1400W
HG+oxLDL
-Vc
70 kDa
55 kDa
Tubulin (50 kDa)
40 kDa
Total GNAI2 (38 kDa)
35 kDa
25 kDa
15 kDa
SNO-GNAI2
(38 kDa)
2: N=1
Mannitol+nLDL
HG+oxLDL+1400W
HG+oxLDL
Mannitol+nLDL
HG+oxLDL+1400W
-Vc
HG+oxLDL
-Vc
70 kDa
55 kDa
Tubulin (50 kDa)
40 kDa
Total GNAI2 (38 kDa)
35 kDa
25 kDa
SNO-GNAI2
(38 kDa)
15 kDa
3: N=1
Mannitol+nLDL
HG+oxLDL+1400W
HG+oxLDL
-Vc
Mannitol+nLDL
HG+oxLDL+1400W
HG+oxLDL
-Vc
70 kDa
55 kDa
Tubulin (50 kDa)
40 kDa
Total GNAI2 (38 kDa)
35 kDa
25 kDa
SNO-GNAI2
(38 kDa)
15 kDa

## Slide 21
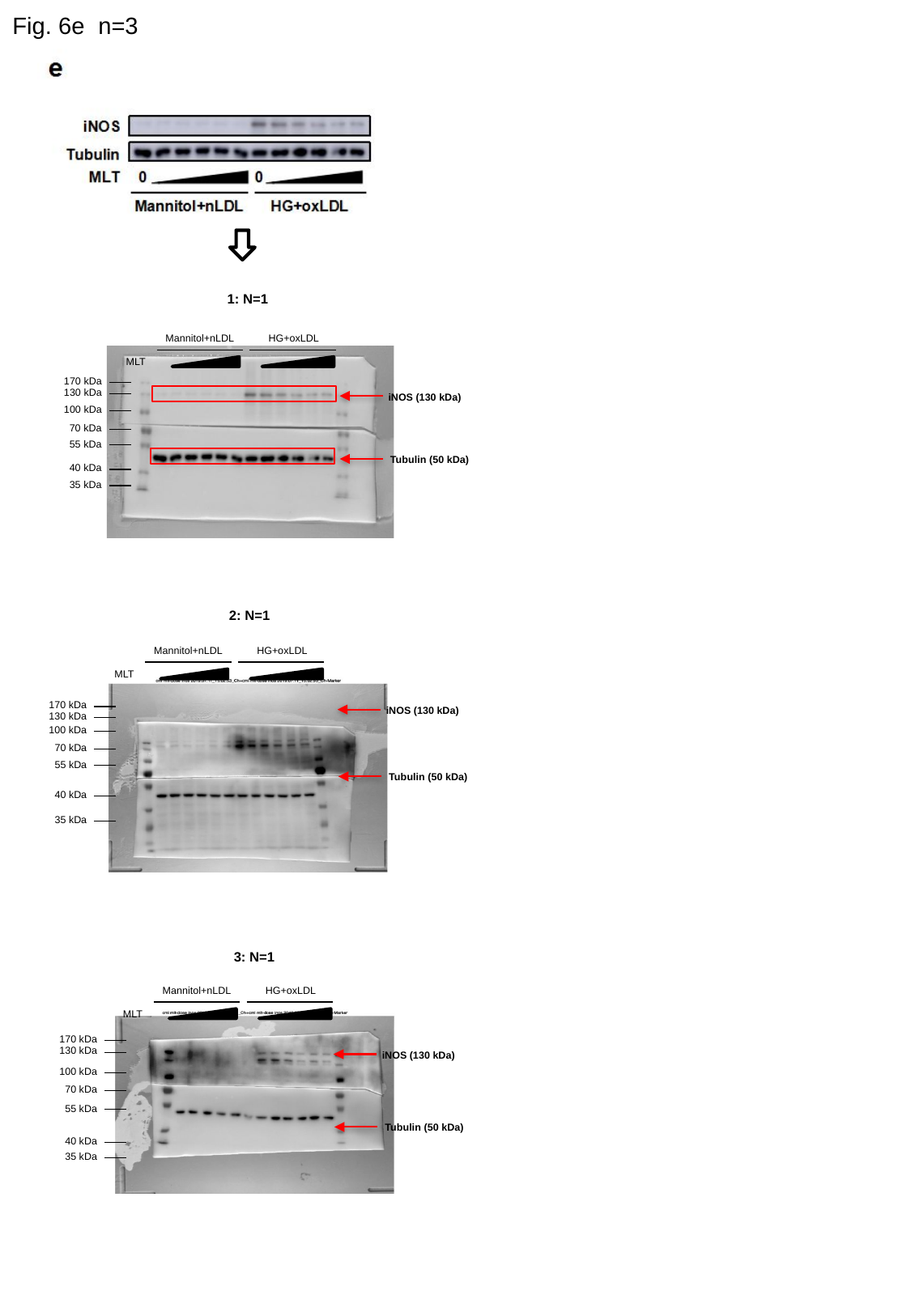

Fig. 6e n=3
1: N=1
Mannitol+nLDL
HG+oxLDL
MLT
170 kDa
130 kDa
iNOS (130 kDa)
100 kDa
70 kDa
55 kDa
Tubulin (50 kDa)
40 kDa
35 kDa
2: N=1
Mannitol+nLDL
HG+oxLDL
MLT
170 kDa
iNOS (130 kDa)
130 kDa
100 kDa
70 kDa
55 kDa
Tubulin (50 kDa)
40 kDa
35 kDa
3: N=1
Mannitol+nLDL
HG+oxLDL
MLT
170 kDa
130 kDa
iNOS (130 kDa)
100 kDa
70 kDa
55 kDa
Tubulin (50 kDa)
40 kDa
35 kDa

## Slide 22
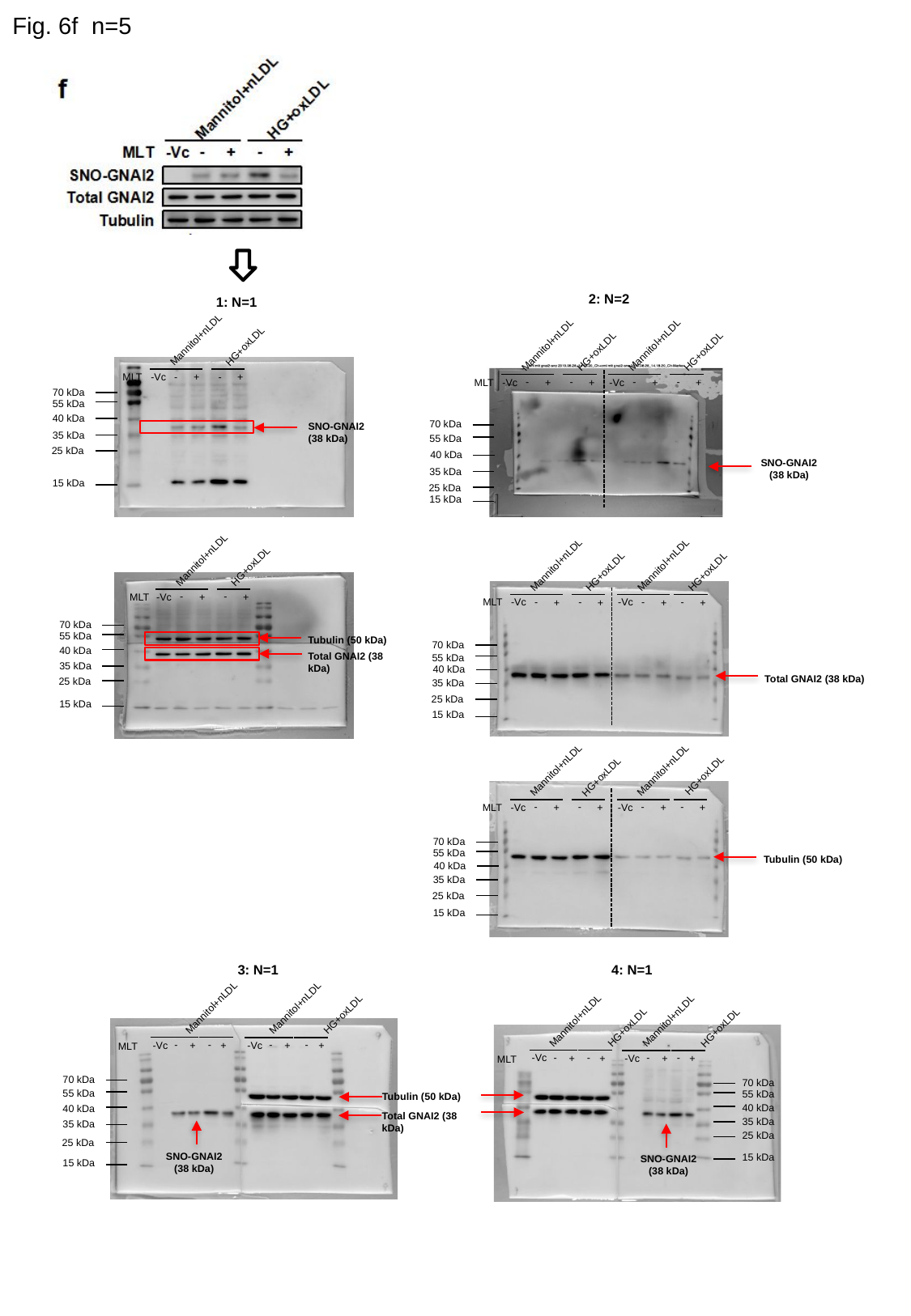

Fig. 6f n=5
2: N=2
1: N=1
HG+oxLDL
Mannitol+nLDL
HG+oxLDL
HG+oxLDL
Mannitol+nLDL
Mannitol+nLDL
-
-
MLT
-Vc
+
+
-
-
-
-
-Vc
MLT
-Vc
+
+
+
+
70 kDa
55 kDa
40 kDa
70 kDa
SNO-GNAI2
(38 kDa)
35 kDa
55 kDa
25 kDa
40 kDa
SNO-GNAI2
(38 kDa)
35 kDa
15 kDa
25 kDa
15 kDa
HG+oxLDL
Mannitol+nLDL
HG+oxLDL
HG+oxLDL
Mannitol+nLDL
Mannitol+nLDL
-
-
MLT
-Vc
+
+
-
-
-
-
-Vc
MLT
-Vc
+
+
+
+
70 kDa
55 kDa
Tubulin (50 kDa)
70 kDa
40 kDa
Total GNAI2 (38 kDa)
55 kDa
35 kDa
40 kDa
Total GNAI2 (38 kDa)
25 kDa
35 kDa
25 kDa
15 kDa
15 kDa
HG+oxLDL
HG+oxLDL
Mannitol+nLDL
Mannitol+nLDL
-
-
-
-
-Vc
MLT
-Vc
+
+
+
+
70 kDa
55 kDa
Tubulin (50 kDa)
40 kDa
35 kDa
25 kDa
15 kDa
3: N=1
4: N=1
HG+oxLDL
Mannitol+nLDL
Mannitol+nLDL
HG+oxLDL
HG+oxLDL
Mannitol+nLDL
Mannitol+nLDL
-
-
-
-
-Vc
-Vc
+
+
+
+
MLT
-
-
-
-
-Vc
-Vc
+
+
+
+
MLT
70 kDa
70 kDa
55 kDa
55 kDa
Tubulin (50 kDa)
40 kDa
40 kDa
Total GNAI2 (38 kDa)
35 kDa
35 kDa
25 kDa
25 kDa
SNO-GNAI2
(38 kDa)
15 kDa
SNO-GNAI2
(38 kDa)
15 kDa

## Slide 23
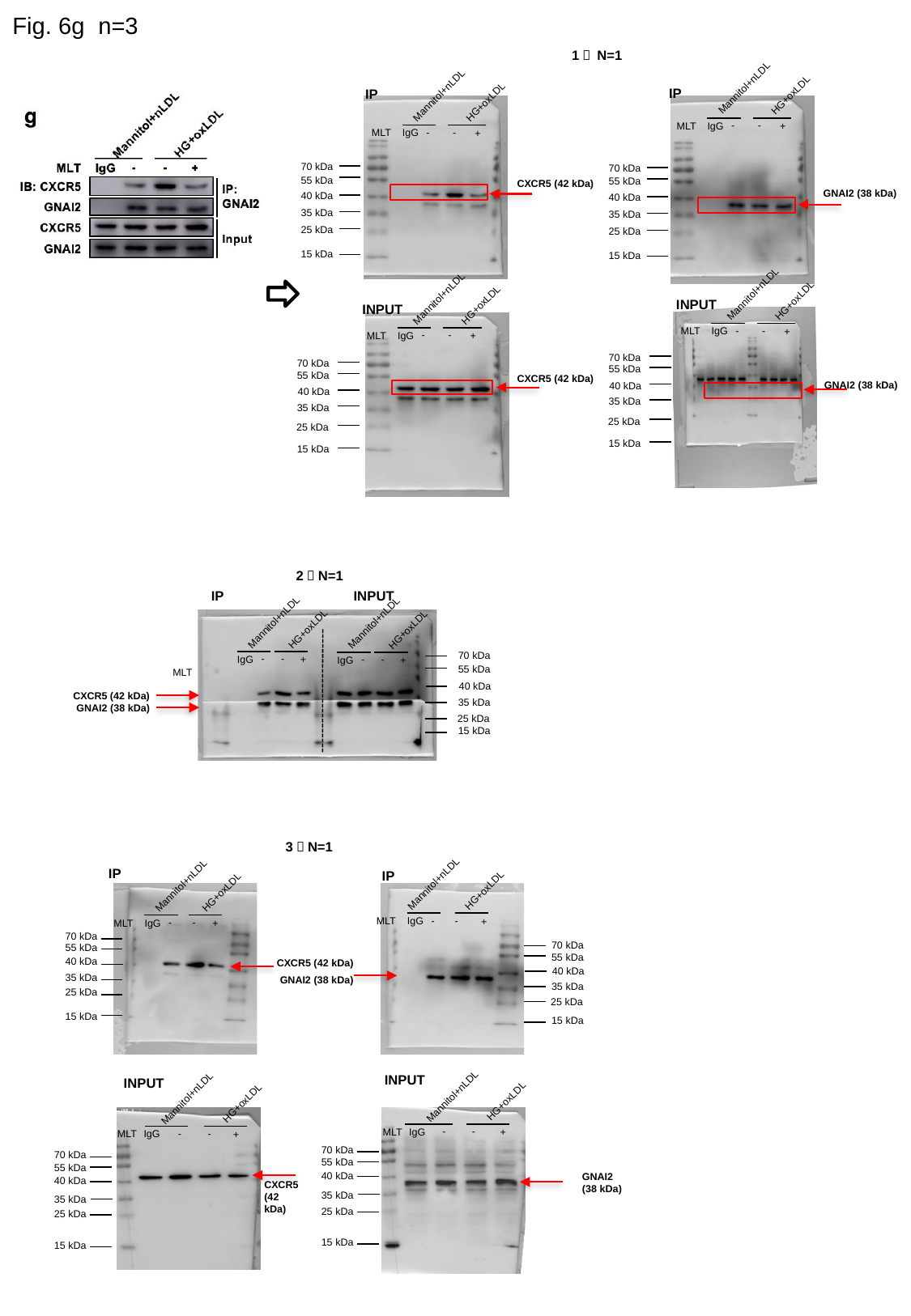

Fig. 6g n=3
1： N=1
Mannitol+nLDL
IP
IP
HG+oxLDL
Mannitol+nLDL
HG+oxLDL
-
-
MLT
IgG
+
-
-
MLT
IgG
+
70 kDa
70 kDa
55 kDa
55 kDa
CXCR5 (42 kDa)
GNAI2 (38 kDa)
40 kDa
40 kDa
35 kDa
35 kDa
25 kDa
25 kDa
15 kDa
15 kDa
Mannitol+nLDL
Mannitol+nLDL
HG+oxLDL
HG+oxLDL
INPUT
INPUT
-
-
MLT
IgG
+
-
-
MLT
IgG
+
70 kDa
70 kDa
55 kDa
55 kDa
CXCR5 (42 kDa)
GNAI2 (38 kDa)
40 kDa
40 kDa
35 kDa
35 kDa
25 kDa
25 kDa
15 kDa
15 kDa
2：N=1
IP
INPUT
HG+oxLDL
HG+oxLDL
Mannitol+nLDL
Mannitol+nLDL
70 kDa
-
-
IgG
-
-
+
IgG
+
55 kDa
MLT
40 kDa
CXCR5 (42 kDa)
35 kDa
GNAI2 (38 kDa)
25 kDa
15 kDa
3：N=1
IP
IP
Mannitol+nLDL
Mannitol+nLDL
HG+oxLDL
HG+oxLDL
-
-
MLT
IgG
+
-
-
MLT
IgG
+
70 kDa
70 kDa
55 kDa
55 kDa
40 kDa
CXCR5 (42 kDa)
40 kDa
35 kDa
GNAI2 (38 kDa)
35 kDa
25 kDa
25 kDa
15 kDa
15 kDa
INPUT
INPUT
Mannitol+nLDL
Mannitol+nLDL
HG+oxLDL
HG+oxLDL
-
-
MLT
IgG
+
-
-
MLT
IgG
+
70 kDa
70 kDa
55 kDa
55 kDa
40 kDa
GNAI2
(38 kDa)
40 kDa
CXCR5
(42 kDa)
35 kDa
35 kDa
25 kDa
25 kDa
15 kDa
15 kDa

## Slide 24
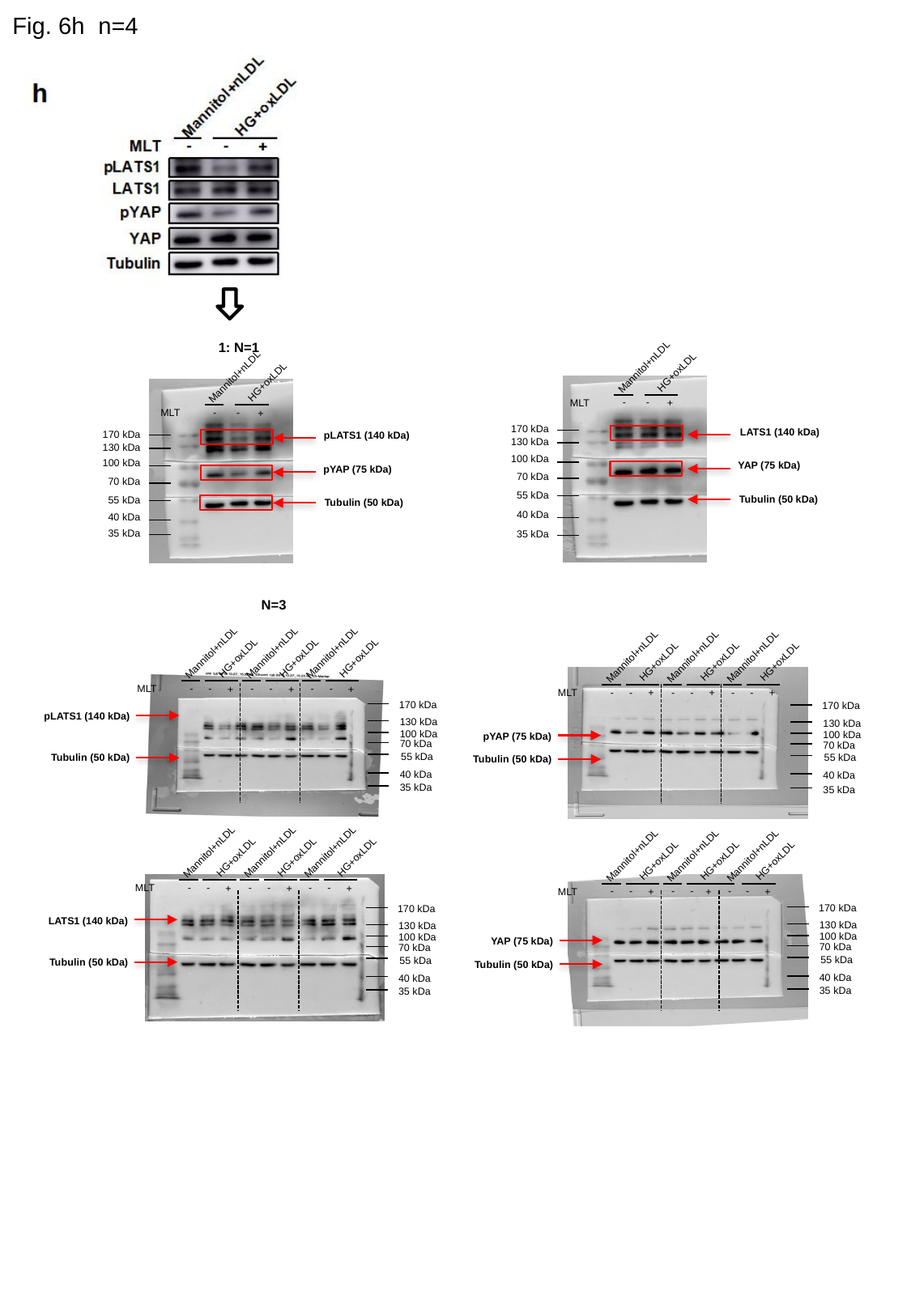

Fig. 6h n=4
1: N=1
Mannitol+nLDL
HG+oxLDL
Mannitol+nLDL
HG+oxLDL
-
-
MLT
+
-
-
MLT
+
170 kDa
LATS1 (140 kDa)
170 kDa
pLATS1 (140 kDa)
130 kDa
130 kDa
100 kDa
100 kDa
YAP (75 kDa)
pYAP (75 kDa)
70 kDa
70 kDa
55 kDa
Tubulin (50 kDa)
55 kDa
Tubulin (50 kDa)
40 kDa
40 kDa
35 kDa
35 kDa
N=3
Mannitol+nLDL
Mannitol+nLDL
Mannitol+nLDL
Mannitol+nLDL
Mannitol+nLDL
Mannitol+nLDL
HG+oxLDL
HG+oxLDL
HG+oxLDL
HG+oxLDL
HG+oxLDL
HG+oxLDL
-
-
-
-
-
-
MLT
+
+
+
-
-
-
-
-
-
MLT
+
+
+
170 kDa
170 kDa
pLATS1 (140 kDa)
130 kDa
130 kDa
100 kDa
100 kDa
pYAP (75 kDa)
70 kDa
70 kDa
55 kDa
Tubulin (50 kDa)
55 kDa
Tubulin (50 kDa)
40 kDa
40 kDa
35 kDa
35 kDa
Mannitol+nLDL
Mannitol+nLDL
Mannitol+nLDL
Mannitol+nLDL
Mannitol+nLDL
Mannitol+nLDL
HG+oxLDL
HG+oxLDL
HG+oxLDL
HG+oxLDL
HG+oxLDL
HG+oxLDL
-
-
-
-
-
-
MLT
+
+
+
-
-
-
-
-
-
MLT
+
+
+
170 kDa
170 kDa
LATS1 (140 kDa)
130 kDa
130 kDa
100 kDa
100 kDa
YAP (75 kDa)
70 kDa
70 kDa
55 kDa
55 kDa
Tubulin (50 kDa)
Tubulin (50 kDa)
40 kDa
40 kDa
35 kDa
35 kDa

## Slide 25
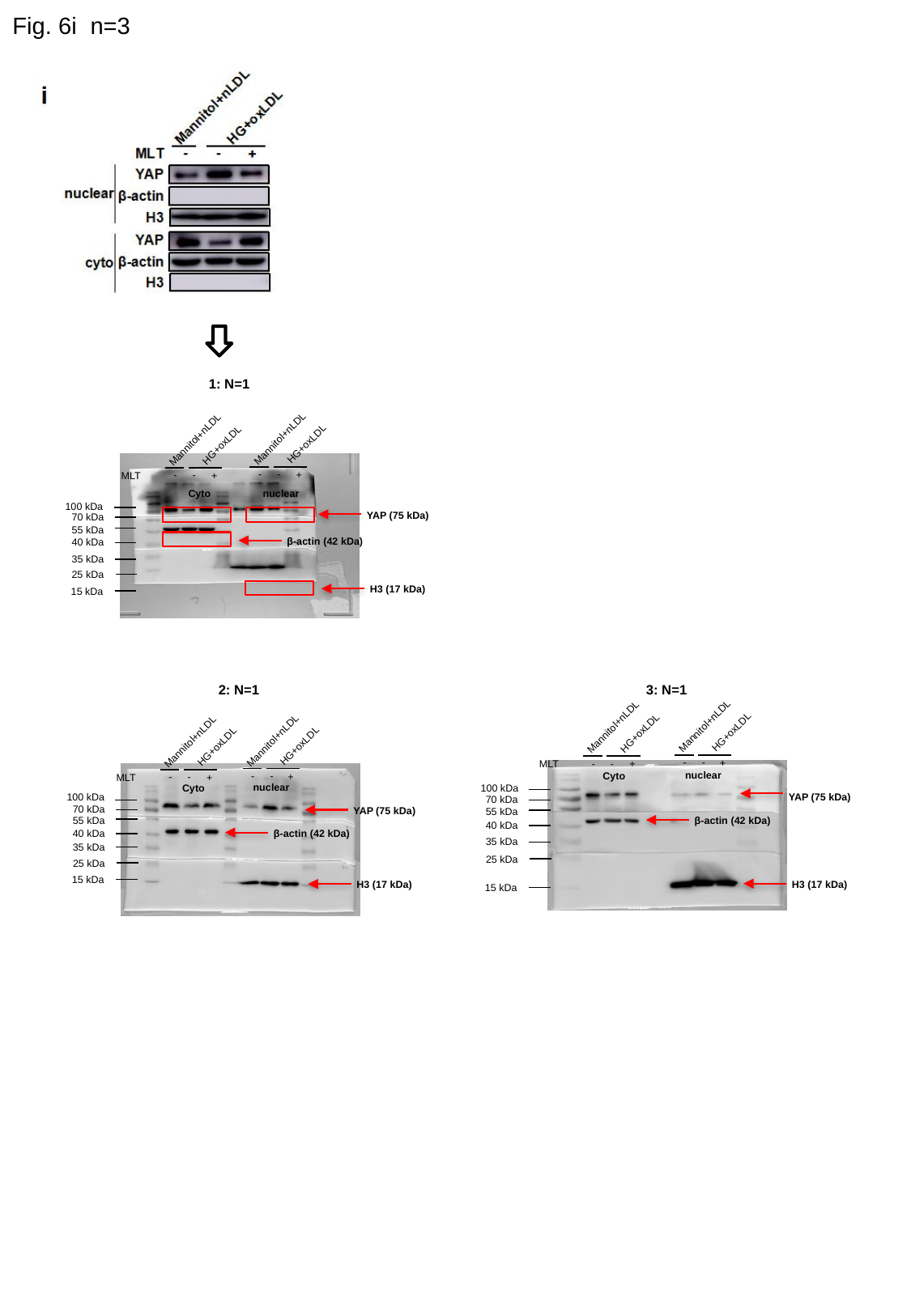

Fig. 6i n=3
1: N=1
Mannitol+nLDL
Mannitol+nLDL
HG+oxLDL
HG+oxLDL
-
-
+
-
-
MLT
+
nuclear
Cyto
100 kDa
YAP (75 kDa)
70 kDa
55 kDa
β-actin (42 kDa)
40 kDa
35 kDa
25 kDa
H3 (17 kDa)
15 kDa
2: N=1
3: N=1
Mannitol+nLDL
Mannitol+nLDL
HG+oxLDL
HG+oxLDL
Mannitol+nLDL
Mannitol+nLDL
HG+oxLDL
HG+oxLDL
-
-
+
-
-
MLT
+
nuclear
Cyto
-
-
+
-
-
MLT
+
nuclear
Cyto
100 kDa
YAP (75 kDa)
100 kDa
70 kDa
70 kDa
YAP (75 kDa)
55 kDa
55 kDa
β-actin (42 kDa)
40 kDa
40 kDa
β-actin (42 kDa)
35 kDa
35 kDa
25 kDa
25 kDa
15 kDa
H3 (17 kDa)
H3 (17 kDa)
15 kDa

## Slide 26
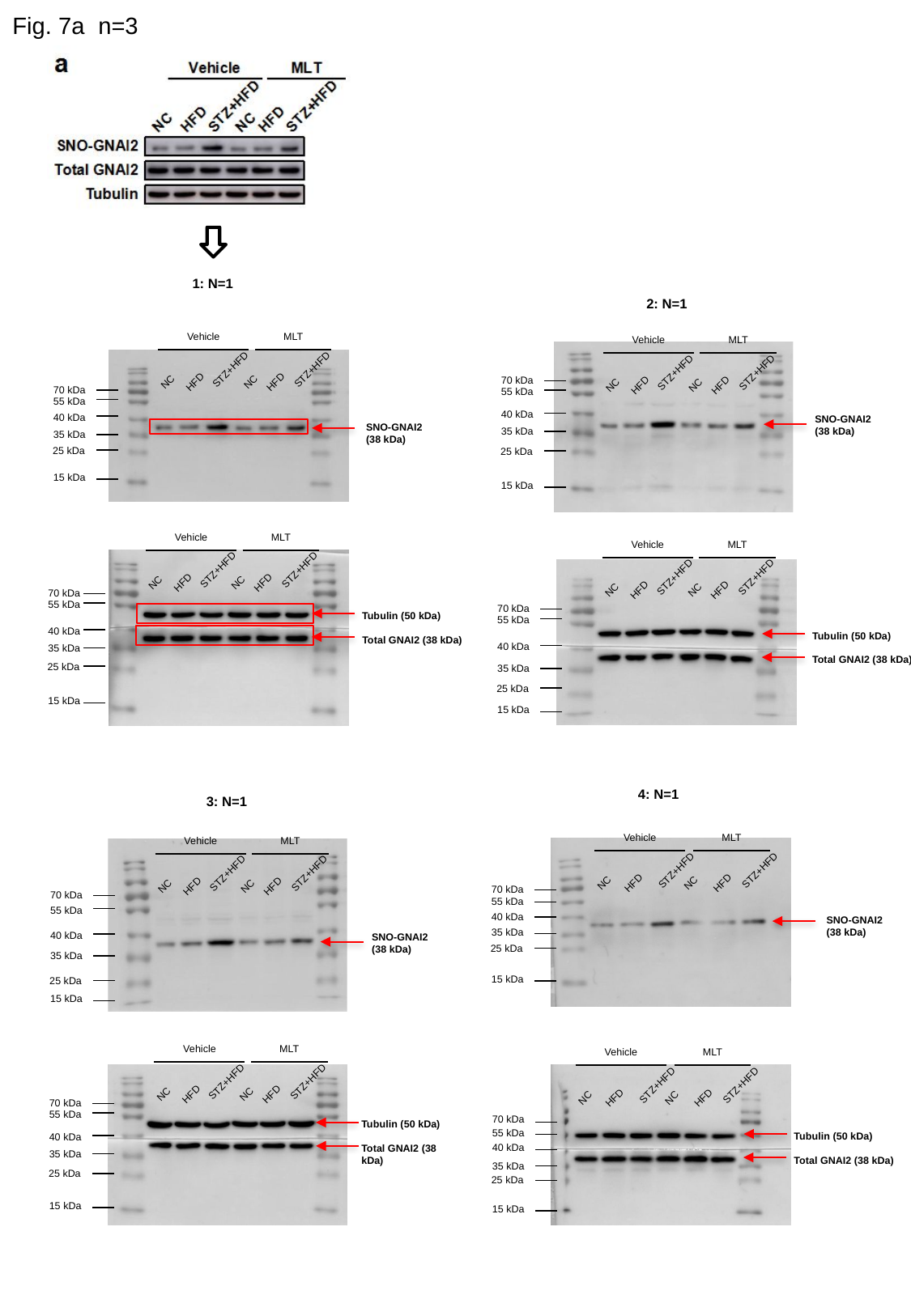

Fig. 7a n=3
1: N=1
2: N=1
Vehicle
MLT
Vehicle
MLT
STZ+HFD
STZ+HFD
STZ+HFD
STZ+HFD
70 kDa
NC
HFD
NC
HFD
NC
HFD
NC
HFD
70 kDa
55 kDa
55 kDa
40 kDa
40 kDa
SNO-GNAI2
(38 kDa)
SNO-GNAI2
(38 kDa)
35 kDa
35 kDa
25 kDa
25 kDa
15 kDa
15 kDa
Vehicle
MLT
Vehicle
MLT
STZ+HFD
STZ+HFD
STZ+HFD
STZ+HFD
NC
HFD
NC
HFD
NC
HFD
NC
HFD
70 kDa
55 kDa
70 kDa
Tubulin (50 kDa)
55 kDa
40 kDa
Tubulin (50 kDa)
Total GNAI2 (38 kDa)
40 kDa
35 kDa
Total GNAI2 (38 kDa)
25 kDa
35 kDa
25 kDa
15 kDa
15 kDa
4: N=1
3: N=1
Vehicle
MLT
Vehicle
MLT
STZ+HFD
STZ+HFD
STZ+HFD
STZ+HFD
NC
HFD
NC
HFD
NC
HFD
NC
HFD
70 kDa
70 kDa
55 kDa
55 kDa
40 kDa
SNO-GNAI2
(38 kDa)
35 kDa
40 kDa
SNO-GNAI2
(38 kDa)
25 kDa
35 kDa
15 kDa
25 kDa
15 kDa
Vehicle
MLT
Vehicle
MLT
STZ+HFD
STZ+HFD
STZ+HFD
STZ+HFD
NC
HFD
NC
HFD
NC
HFD
NC
HFD
70 kDa
55 kDa
70 kDa
Tubulin (50 kDa)
55 kDa
Tubulin (50 kDa)
40 kDa
40 kDa
Total GNAI2 (38 kDa)
35 kDa
Total GNAI2 (38 kDa)
35 kDa
25 kDa
25 kDa
15 kDa
15 kDa

## Slide 27
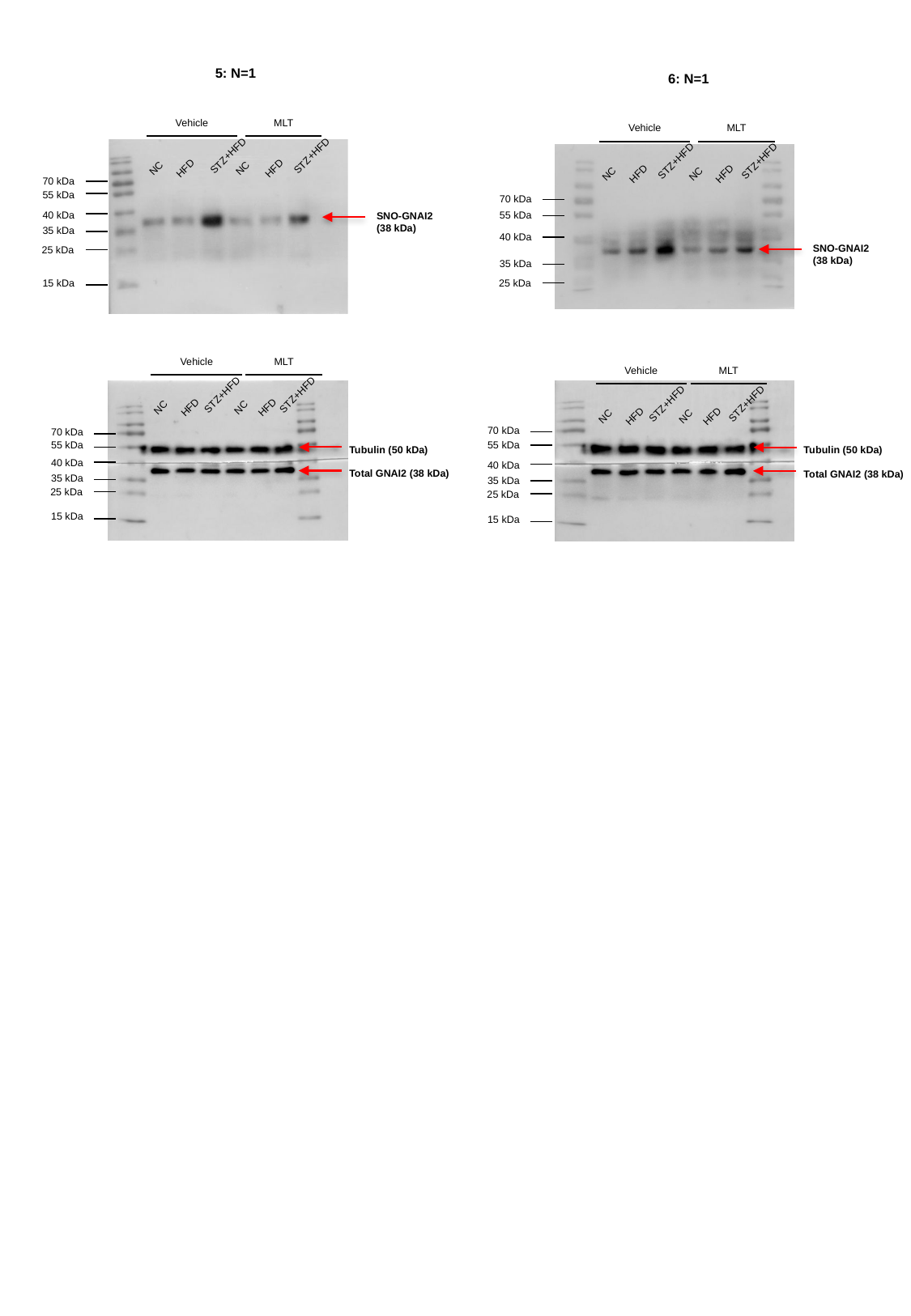

5: N=1
6: N=1
Vehicle
MLT
Vehicle
MLT
STZ+HFD
STZ+HFD
STZ+HFD
STZ+HFD
NC
HFD
NC
HFD
NC
HFD
NC
HFD
70 kDa
55 kDa
70 kDa
40 kDa
55 kDa
SNO-GNAI2
(38 kDa)
35 kDa
40 kDa
SNO-GNAI2
(38 kDa)
25 kDa
35 kDa
25 kDa
15 kDa
Vehicle
MLT
Vehicle
MLT
STZ+HFD
STZ+HFD
STZ+HFD
STZ+HFD
NC
HFD
NC
HFD
NC
HFD
NC
HFD
70 kDa
70 kDa
55 kDa
55 kDa
Tubulin (50 kDa)
Tubulin (50 kDa)
40 kDa
40 kDa
Total GNAI2 (38 kDa)
Total GNAI2 (38 kDa)
35 kDa
35 kDa
25 kDa
25 kDa
15 kDa
15 kDa

## Slide 28
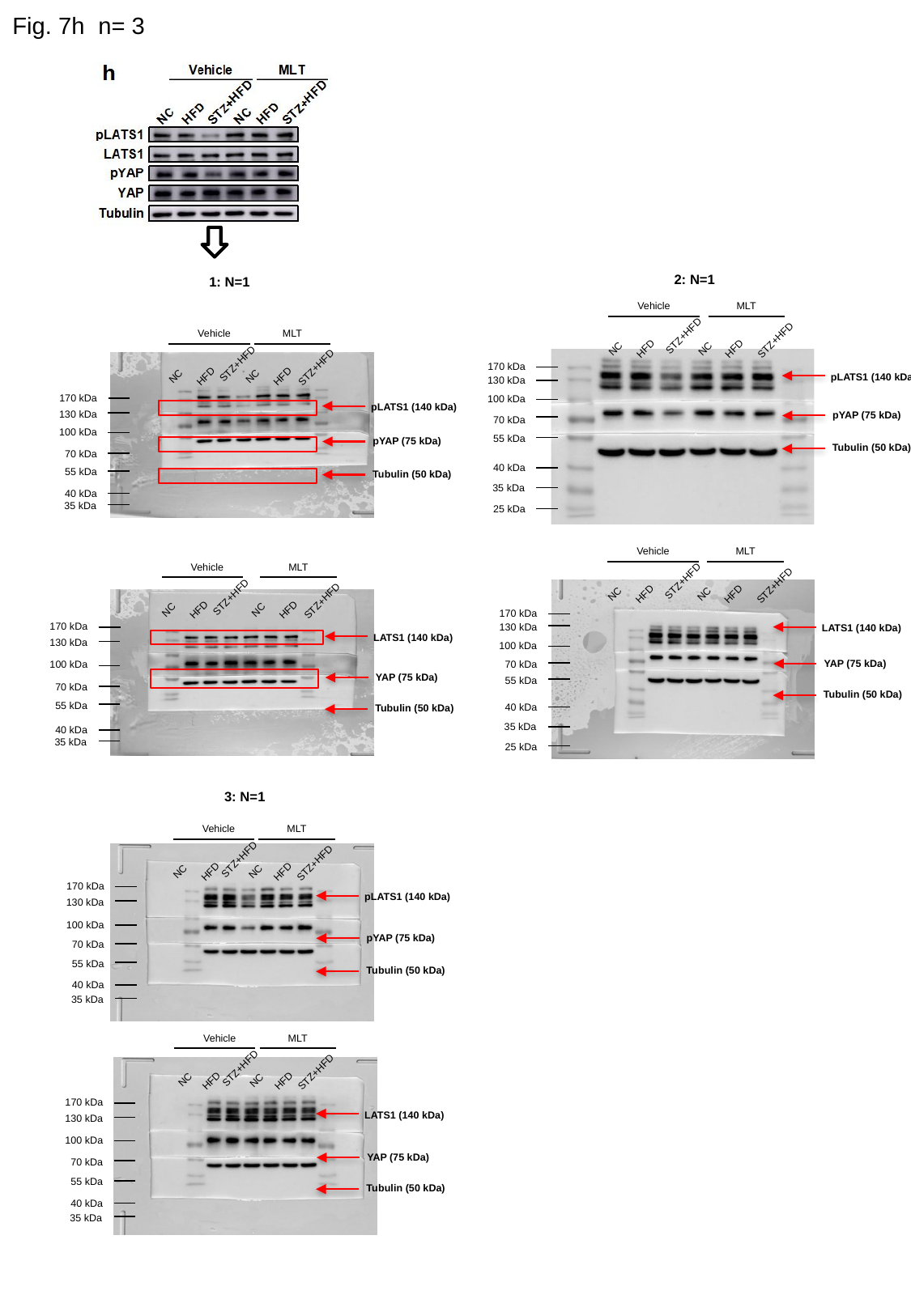

Fig. 7h n= 3
2: N=1
1: N=1
Vehicle
MLT
Vehicle
MLT
STZ+HFD
STZ+HFD
NC
HFD
NC
HFD
STZ+HFD
170 kDa
STZ+HFD
NC
HFD
NC
HFD
pLATS1 (140 kDa)
130 kDa
170 kDa
100 kDa
pLATS1 (140 kDa)
130 kDa
pYAP (75 kDa)
70 kDa
100 kDa
55 kDa
pYAP (75 kDa)
Tubulin (50 kDa)
70 kDa
40 kDa
55 kDa
Tubulin (50 kDa)
35 kDa
40 kDa
35 kDa
25 kDa
Vehicle
MLT
Vehicle
MLT
STZ+HFD
STZ+HFD
NC
HFD
NC
HFD
STZ+HFD
STZ+HFD
NC
HFD
NC
HFD
170 kDa
170 kDa
130 kDa
LATS1 (140 kDa)
LATS1 (140 kDa)
130 kDa
100 kDa
YAP (75 kDa)
70 kDa
100 kDa
YAP (75 kDa)
55 kDa
70 kDa
Tubulin (50 kDa)
55 kDa
40 kDa
Tubulin (50 kDa)
35 kDa
40 kDa
35 kDa
25 kDa
3: N=1
Vehicle
MLT
STZ+HFD
STZ+HFD
NC
HFD
NC
HFD
170 kDa
pLATS1 (140 kDa)
130 kDa
100 kDa
pYAP (75 kDa)
70 kDa
55 kDa
Tubulin (50 kDa)
40 kDa
35 kDa
Vehicle
MLT
STZ+HFD
STZ+HFD
NC
HFD
NC
HFD
170 kDa
LATS1 (140 kDa)
130 kDa
100 kDa
YAP (75 kDa)
70 kDa
55 kDa
Tubulin (50 kDa)
40 kDa
35 kDa

## Slide 29
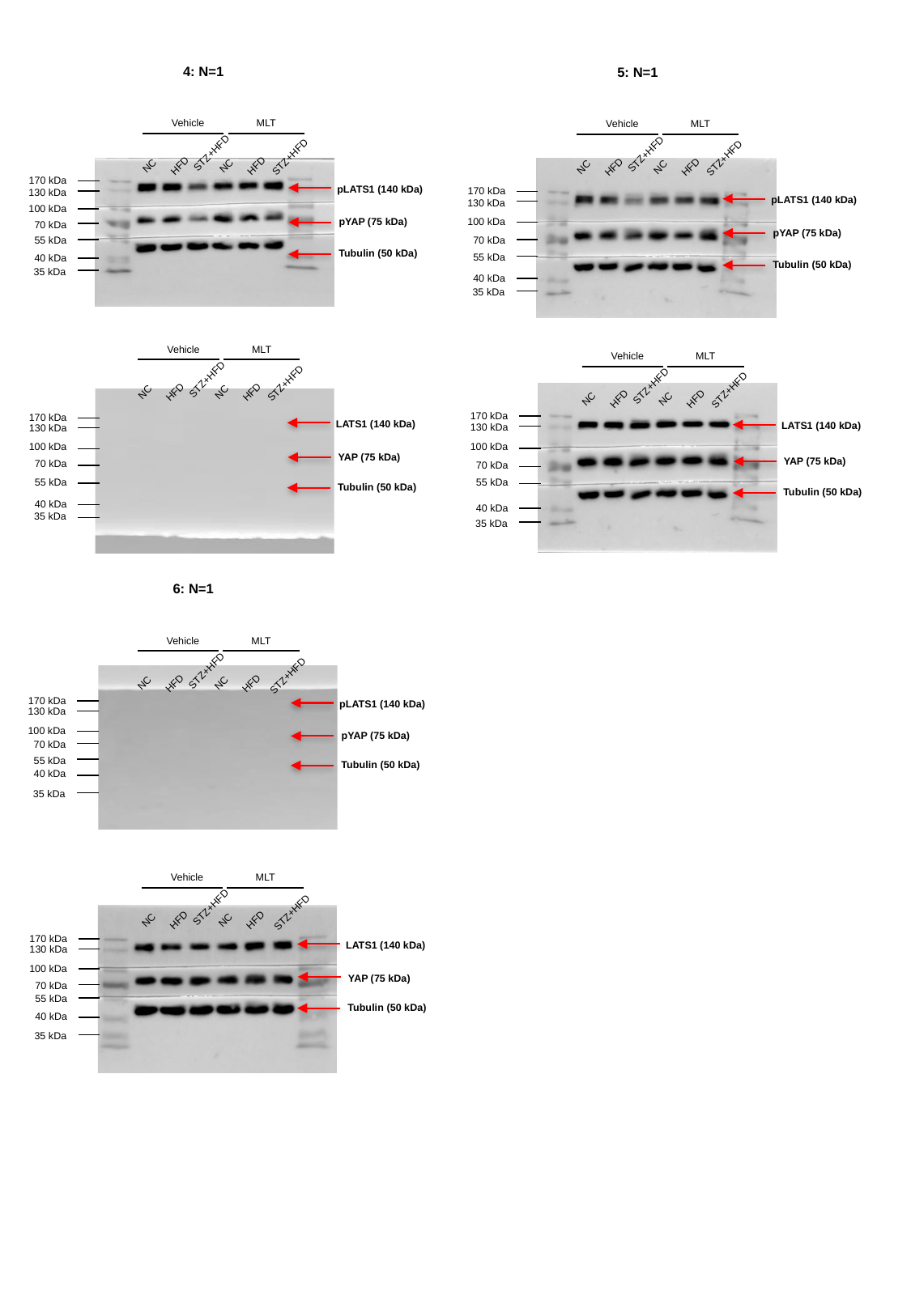

4: N=1
5: N=1
Vehicle
MLT
Vehicle
MLT
STZ+HFD
STZ+HFD
STZ+HFD
STZ+HFD
NC
HFD
NC
HFD
NC
HFD
NC
HFD
170 kDa
pLATS1 (140 kDa)
170 kDa
130 kDa
pLATS1 (140 kDa)
130 kDa
100 kDa
100 kDa
pYAP (75 kDa)
70 kDa
pYAP (75 kDa)
55 kDa
70 kDa
Tubulin (50 kDa)
55 kDa
40 kDa
Tubulin (50 kDa)
35 kDa
40 kDa
35 kDa
Vehicle
MLT
Vehicle
MLT
STZ+HFD
STZ+HFD
STZ+HFD
STZ+HFD
NC
HFD
NC
HFD
NC
HFD
NC
HFD
170 kDa
170 kDa
LATS1 (140 kDa)
LATS1 (140 kDa)
130 kDa
130 kDa
100 kDa
100 kDa
YAP (75 kDa)
YAP (75 kDa)
70 kDa
70 kDa
55 kDa
55 kDa
Tubulin (50 kDa)
Tubulin (50 kDa)
40 kDa
40 kDa
35 kDa
35 kDa
6: N=1
Vehicle
MLT
STZ+HFD
STZ+HFD
NC
HFD
NC
HFD
170 kDa
pLATS1 (140 kDa)
130 kDa
100 kDa
pYAP (75 kDa)
70 kDa
55 kDa
Tubulin (50 kDa)
40 kDa
35 kDa
Vehicle
MLT
STZ+HFD
STZ+HFD
NC
HFD
NC
HFD
170 kDa
LATS1 (140 kDa)
130 kDa
100 kDa
YAP (75 kDa)
70 kDa
55 kDa
Tubulin (50 kDa)
40 kDa
35 kDa

## Slide 30
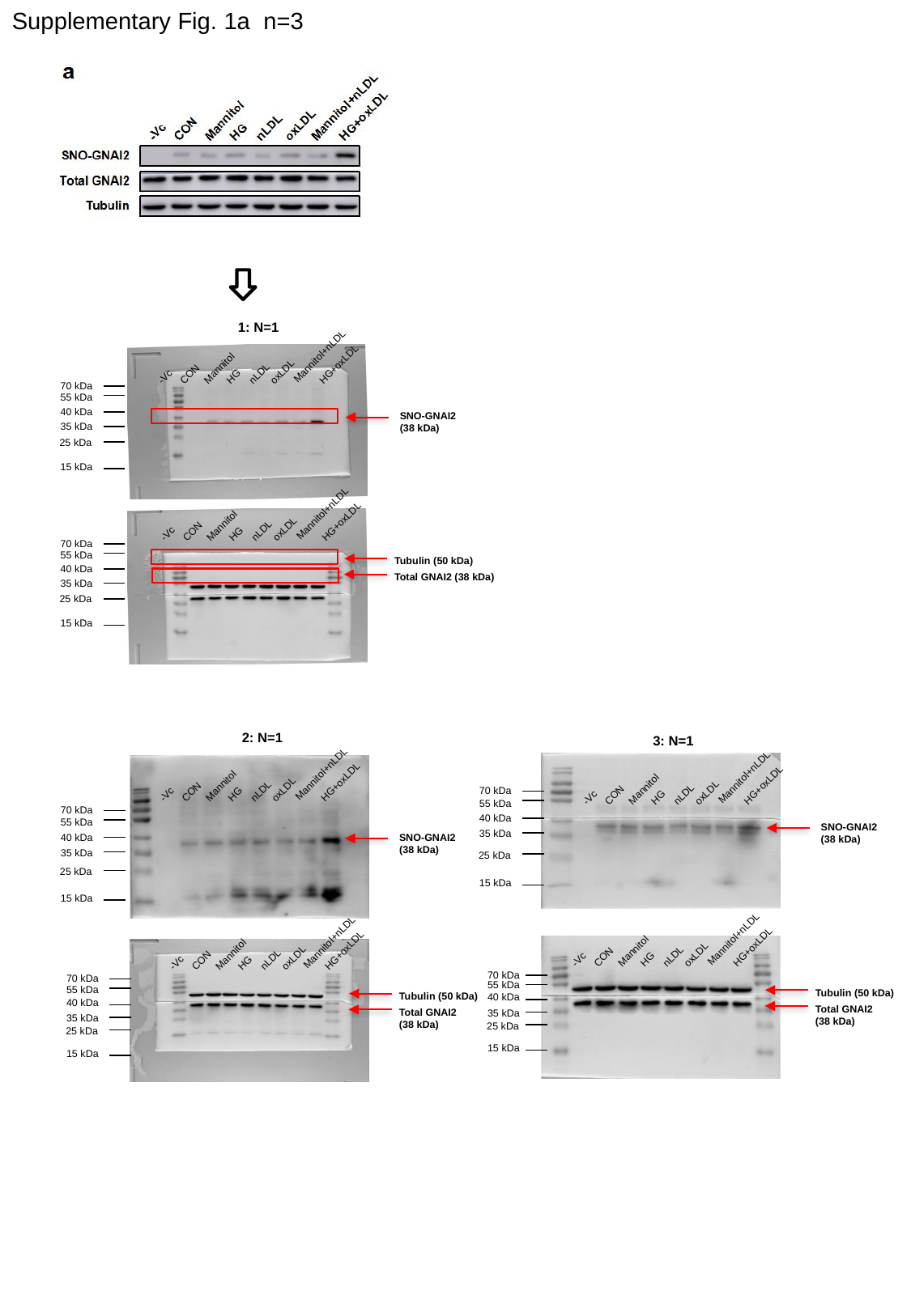

Supplementary Fig. 1a n=3
1: N=1
Mannitol+nLDL
Mannitol
HG
nLDL
oxLDL
HG+oxLDL
-Vc
CON
70 kDa
55 kDa
40 kDa
SNO-GNAI2
(38 kDa)
35 kDa
25 kDa
15 kDa
Mannitol+nLDL
Mannitol
HG
nLDL
oxLDL
HG+oxLDL
-Vc
CON
70 kDa
55 kDa
Tubulin (50 kDa)
40 kDa
Total GNAI2 (38 kDa)
35 kDa
25 kDa
15 kDa
2: N=1
3: N=1
Mannitol+nLDL
Mannitol
HG
nLDL
oxLDL
Mannitol+nLDL
Mannitol
HG
nLDL
oxLDL
HG+oxLDL
HG+oxLDL
-Vc
CON
-Vc
CON
70 kDa
55 kDa
70 kDa
40 kDa
55 kDa
SNO-GNAI2
(38 kDa)
35 kDa
40 kDa
SNO-GNAI2
(38 kDa)
35 kDa
25 kDa
25 kDa
15 kDa
15 kDa
Mannitol+nLDL
Mannitol
HG
nLDL
oxLDL
Mannitol+nLDL
Mannitol
HG
nLDL
oxLDL
HG+oxLDL
HG+oxLDL
-Vc
CON
-Vc
CON
70 kDa
70 kDa
55 kDa
55 kDa
Tubulin (50 kDa)
Tubulin (50 kDa)
40 kDa
40 kDa
Total GNAI2 (38 kDa)
Total GNAI2 (38 kDa)
35 kDa
35 kDa
25 kDa
25 kDa
15 kDa
15 kDa

## Slide 31
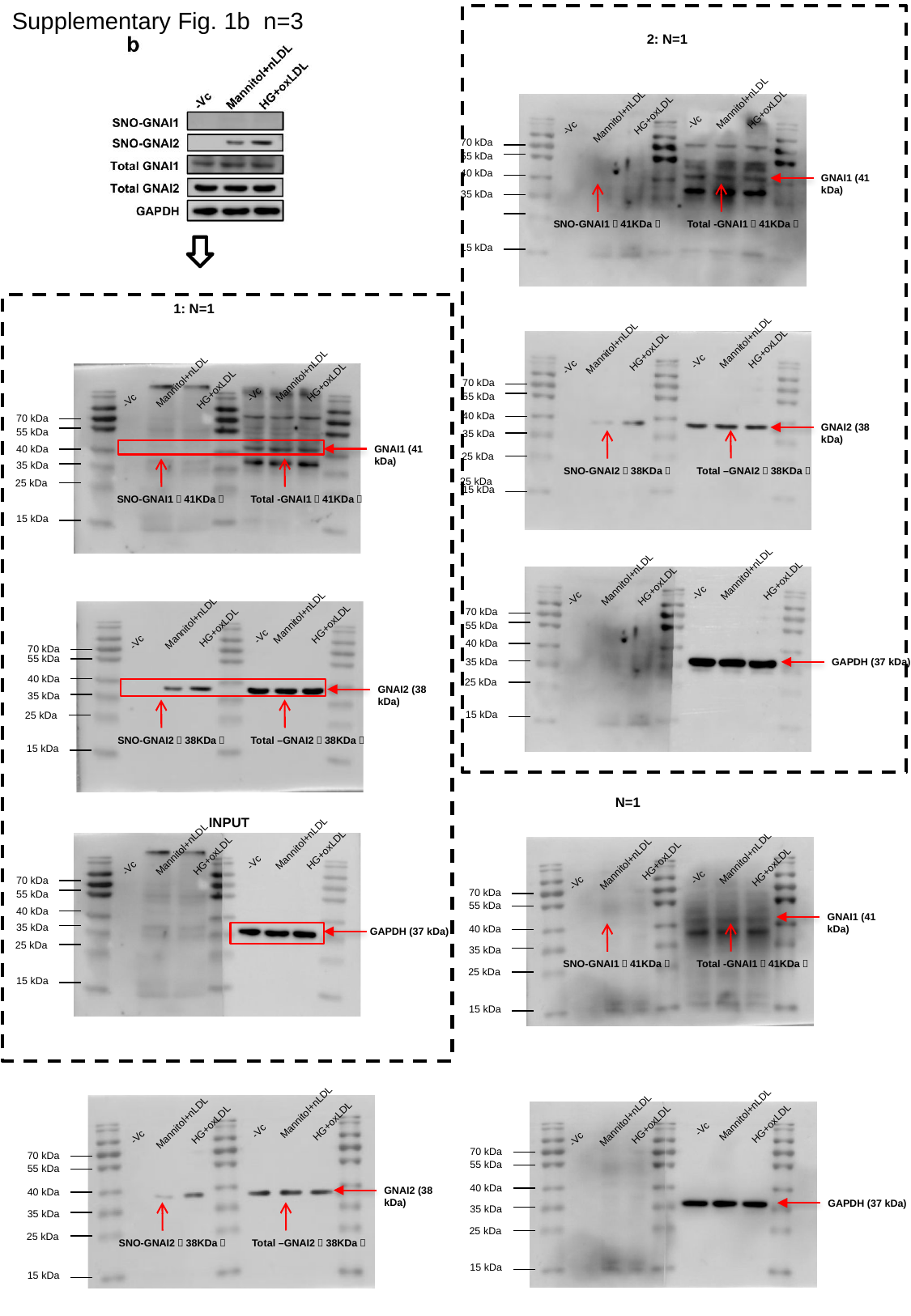

Supplementary Fig. 1b n=3
2: N=1
Mannitol+nLDL
HG+oxLDL
HG+oxLDL
Mannitol+nLDL
-Vc
-Vc
70 kDa
55 kDa
40 kDa
GNAI1 (41 kDa)
35 kDa
SNO-GNAI1（41KDa）
Total -GNAI1（41KDa）
15 kDa
1: N=1
Mannitol+nLDL
HG+oxLDL
HG+oxLDL
Mannitol+nLDL
-Vc
-Vc
Mannitol+nLDL
HG+oxLDL
Mannitol+nLDL
HG+oxLDL
-Vc
-Vc
70 kDa
55 kDa
GNAI1 (41 kDa)
40 kDa
35 kDa
25 kDa
15 kDa
Mannitol+nLDL
HG+oxLDL
HG+oxLDL
Mannitol+nLDL
-Vc
-Vc
70 kDa
55 kDa
40 kDa
GNAI2 (38 kDa)
35 kDa
25 kDa
15 kDa
INPUT
Mannitol+nLDL
HG+oxLDL
Mannitol+nLDL
HG+oxLDL
-Vc
-Vc
70 kDa
55 kDa
40 kDa
35 kDa
GAPDH (37 kDa)
25 kDa
15 kDa
70 kDa
55 kDa
40 kDa
GNAI2 (38 kDa)
35 kDa
25 kDa
SNO-GNAI2（38KDa）
Total –GNAI2（38KDa）
25 kDa
15 kDa
SNO-GNAI1（41KDa）
Total -GNAI1（41KDa）
Mannitol+nLDL
HG+oxLDL
HG+oxLDL
Mannitol+nLDL
-Vc
-Vc
70 kDa
55 kDa
40 kDa
35 kDa
GAPDH (37 kDa)
25 kDa
15 kDa
SNO-GNAI2（38KDa）
Total –GNAI2（38KDa）
N=1
HG+oxLDL
Mannitol+nLDL
HG+oxLDL
Mannitol+nLDL
-Vc
-Vc
70 kDa
55 kDa
GNAI1 (41 kDa)
40 kDa
35 kDa
SNO-GNAI1（41KDa）
Total -GNAI1（41KDa）
25 kDa
15 kDa
Mannitol+nLDL
HG+oxLDL
Mannitol+nLDL
HG+oxLDL
HG+oxLDL
HG+oxLDL
Mannitol+nLDL
Mannitol+nLDL
-Vc
-Vc
-Vc
-Vc
70 kDa
70 kDa
55 kDa
55 kDa
40 kDa
GNAI2 (38 kDa)
40 kDa
GAPDH (37 kDa)
35 kDa
35 kDa
25 kDa
25 kDa
SNO-GNAI2（38KDa）
Total –GNAI2（38KDa）
15 kDa
15 kDa

## Slide 32
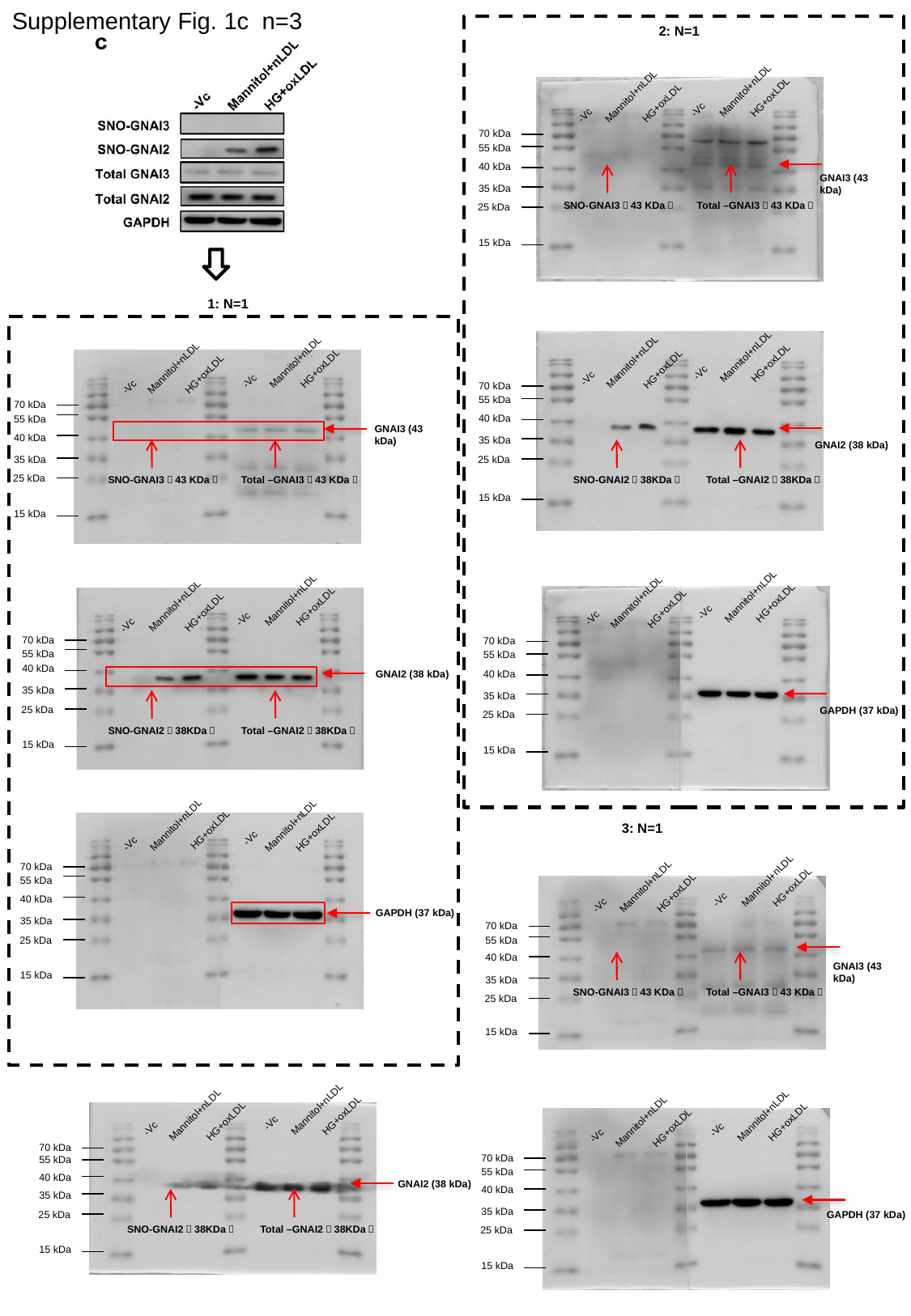

Supplementary Fig. 1c n=3
2: N=1
Mannitol+nLDL
HG+oxLDL
Mannitol+nLDL
-Vc
HG+oxLDL
-Vc
70 kDa
55 kDa
40 kDa
GNAI3 (43 kDa)
35 kDa
SNO-GNAI3（43 KDa）
Total –GNAI3（43 KDa）
25 kDa
15 kDa
1: N=1
Mannitol+nLDL
HG+oxLDL
Mannitol+nLDL
HG+oxLDL
Mannitol+nLDL
-Vc
HG+oxLDL
-Vc
70 kDa
55 kDa
GNAI3 (43 kDa)
40 kDa
35 kDa
25 kDa
15 kDa
Mannitol+nLDL
HG+oxLDL
Mannitol+nLDL
HG+oxLDL
-Vc
-Vc
70 kDa
55 kDa
40 kDa
GNAI2 (38 kDa)
35 kDa
25 kDa
15 kDa
HG+oxLDL
Mannitol+nLDL
Mannitol+nLDL
HG+oxLDL
-Vc
-Vc
70 kDa
55 kDa
40 kDa
GAPDH (37 kDa)
35 kDa
25 kDa
15 kDa
Mannitol+nLDL
-Vc
HG+oxLDL
-Vc
70 kDa
55 kDa
40 kDa
35 kDa
GNAI2 (38 kDa)
25 kDa
SNO-GNAI3（43 KDa）
Total –GNAI3（43 KDa）
SNO-GNAI2（38KDa）
Total –GNAI2（38KDa）
15 kDa
Mannitol+nLDL
HG+oxLDL
Mannitol+nLDL
-Vc
-Vc
HG+oxLDL
70 kDa
55 kDa
40 kDa
35 kDa
GAPDH (37 kDa)
25 kDa
SNO-GNAI2（38KDa）
Total –GNAI2（38KDa）
15 kDa
3: N=1
Mannitol+nLDL
HG+oxLDL
Mannitol+nLDL
-Vc
HG+oxLDL
-Vc
70 kDa
55 kDa
40 kDa
GNAI3 (43 kDa)
35 kDa
SNO-GNAI3（43 KDa）
Total –GNAI3（43 KDa）
25 kDa
15 kDa
Mannitol+nLDL
HG+oxLDL
Mannitol+nLDL
Mannitol+nLDL
HG+oxLDL
Mannitol+nLDL
-Vc
-Vc
-Vc
HG+oxLDL
HG+oxLDL
-Vc
70 kDa
70 kDa
55 kDa
55 kDa
40 kDa
GNAI2 (38 kDa)
40 kDa
35 kDa
35 kDa
25 kDa
GAPDH (37 kDa)
SNO-GNAI2（38KDa）
Total –GNAI2（38KDa）
25 kDa
15 kDa
15 kDa

## Slide 33
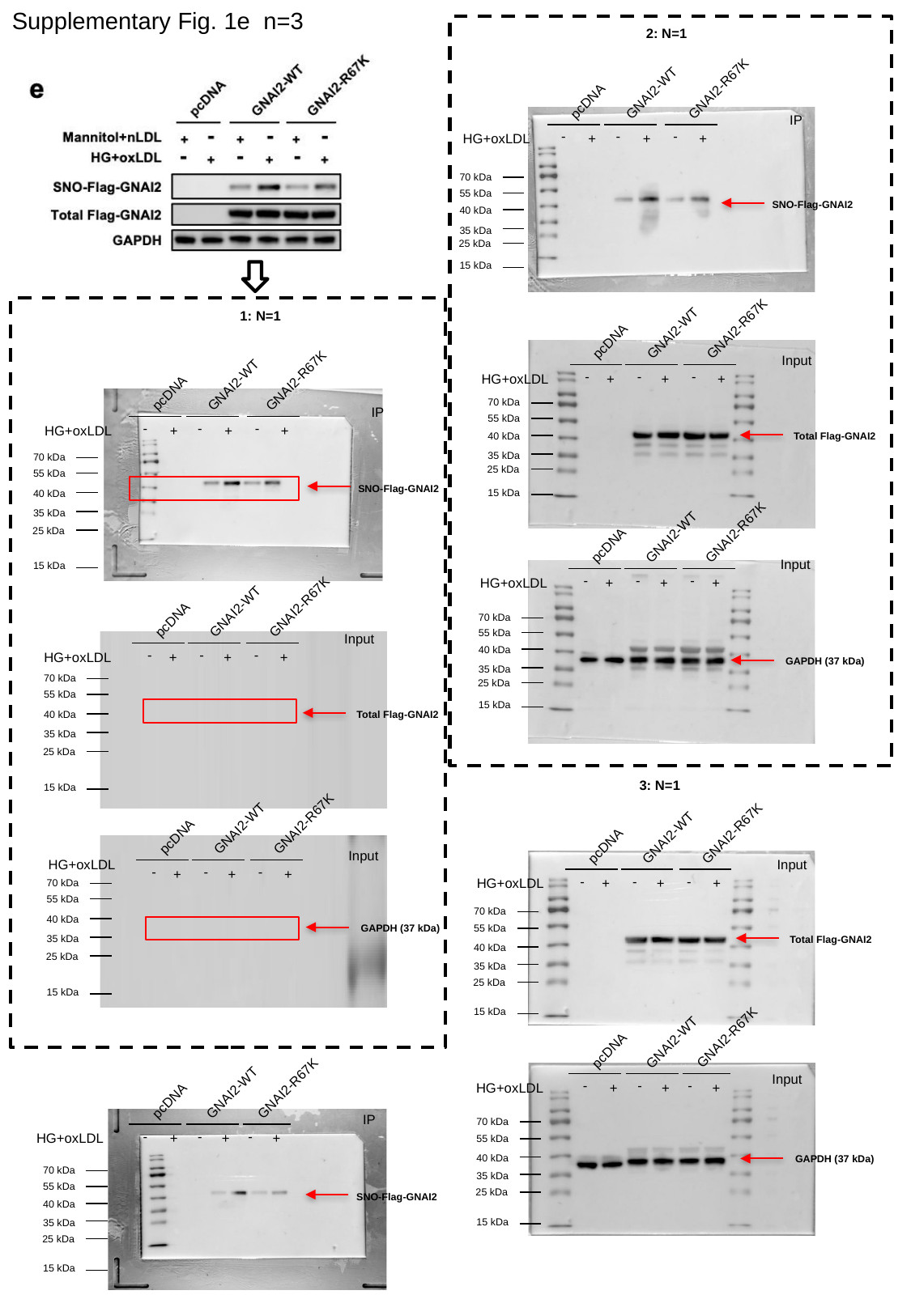

Supplementary Fig. 1e n=3
2: N=1
GNAI2-R67K
pcDNA
GNAI2-WT
IP
-
-
-
HG+oxLDL
+
+
+
70 kDa
55 kDa
SNO-Flag-GNAI2
40 kDa
35 kDa
25 kDa
15 kDa
GNAI2-R67K
1: N=1
pcDNA
GNAI2-WT
GNAI2-R67K
Input
pcDNA
GNAI2-WT
-
-
-
HG+oxLDL
+
+
+
70 kDa
IP
55 kDa
-
-
-
HG+oxLDL
+
+
+
40 kDa
Total Flag-GNAI2
35 kDa
70 kDa
25 kDa
55 kDa
SNO-Flag-GNAI2
15 kDa
40 kDa
GNAI2-R67K
35 kDa
pcDNA
GNAI2-WT
25 kDa
Input
15 kDa
-
-
-
HG+oxLDL
+
+
+
GNAI2-R67K
pcDNA
GNAI2-WT
70 kDa
55 kDa
Input
40 kDa
-
-
-
HG+oxLDL
+
+
+
GAPDH (37 kDa)
35 kDa
70 kDa
25 kDa
55 kDa
15 kDa
Total Flag-GNAI2
40 kDa
35 kDa
25 kDa
3: N=1
15 kDa
GNAI2-R67K
GNAI2-R67K
pcDNA
GNAI2-WT
pcDNA
GNAI2-WT
Input
Input
HG+oxLDL
-
-
-
+
+
+
-
-
-
HG+oxLDL
+
+
+
70 kDa
55 kDa
70 kDa
40 kDa
GAPDH (37 kDa)
55 kDa
35 kDa
Total Flag-GNAI2
40 kDa
25 kDa
35 kDa
25 kDa
15 kDa
15 kDa
pcDNA
GNAI2-WT
GNAI2-R67K
GNAI2-R67K
pcDNA
GNAI2-WT
Input
-
-
-
HG+oxLDL
+
+
+
IP
70 kDa
-
-
-
HG+oxLDL
+
+
+
55 kDa
40 kDa
GAPDH (37 kDa)
70 kDa
35 kDa
55 kDa
25 kDa
SNO-Flag-GNAI2
40 kDa
15 kDa
35 kDa
25 kDa
15 kDa

## Slide 34
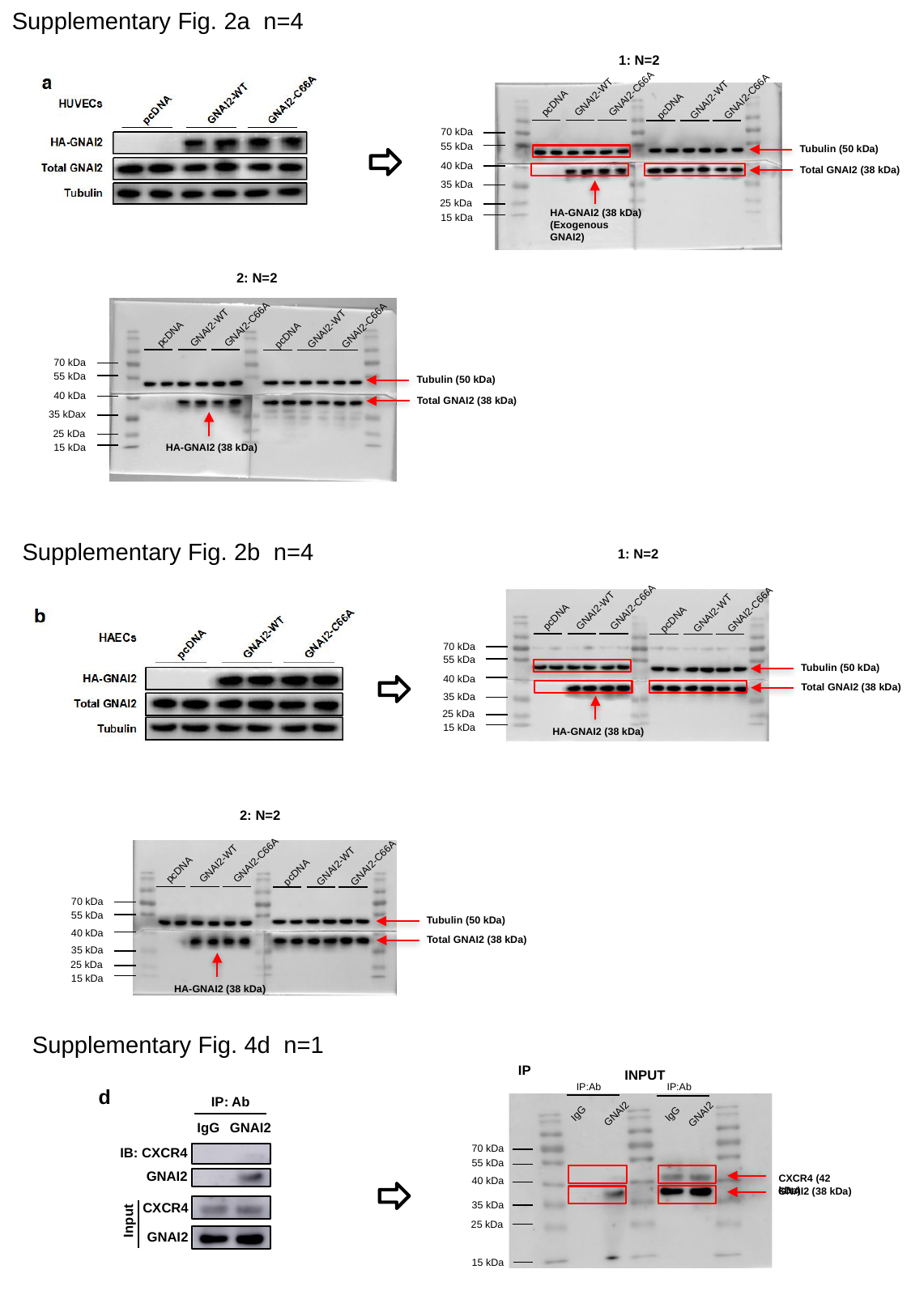

Supplementary Fig. 2a n=4
1: N=2
GNAI2-C66A
pcDNA
GNAI2-WT
GNAI2-C66A
pcDNA
GNAI2-WT
70 kDa
55 kDa
Tubulin (50 kDa)
40 kDa
Total GNAI2 (38 kDa)
35 kDa
25 kDa
HA-GNAI2 (38 kDa)
(Exogenous GNAI2)
15 kDa
2: N=2
GNAI2-C66A
GNAI2-C66A
pcDNA
GNAI2-WT
pcDNA
GNAI2-WT
70 kDa
55 kDa
Tubulin (50 kDa)
40 kDa
Total GNAI2 (38 kDa)
35 kDax
25 kDa
15 kDa
HA-GNAI2 (38 kDa)
Supplementary Fig. 2b n=4
1: N=2
GNAI2-C66A
pcDNA
GNAI2-WT
GNAI2-C66A
pcDNA
GNAI2-WT
70 kDa
55 kDa
Tubulin (50 kDa)
40 kDa
Total GNAI2 (38 kDa)
35 kDa
25 kDa
15 kDa
HA-GNAI2 (38 kDa)
2: N=2
GNAI2-C66A
pcDNA
GNAI2-WT
GNAI2-C66A
pcDNA
GNAI2-WT
70 kDa
55 kDa
Tubulin (50 kDa)
40 kDa
Total GNAI2 (38 kDa)
35 kDa
25 kDa
15 kDa
HA-GNAI2 (38 kDa)
Supplementary Fig. 4d n=1
IP
INPUT
IP:Ab
IP:Ab
d
IP: Ab
IgG
GNAI2
IB: CXCR4
GNAI2
CXCR4
Input
GNAI2
IgG
GNAI2
IgG
GNAI2
70 kDa
55 kDa
CXCR4 (42 kDa)
40 kDa
GNAI2 (38 kDa)
35 kDa
25 kDa
15 kDa

## Slide 35
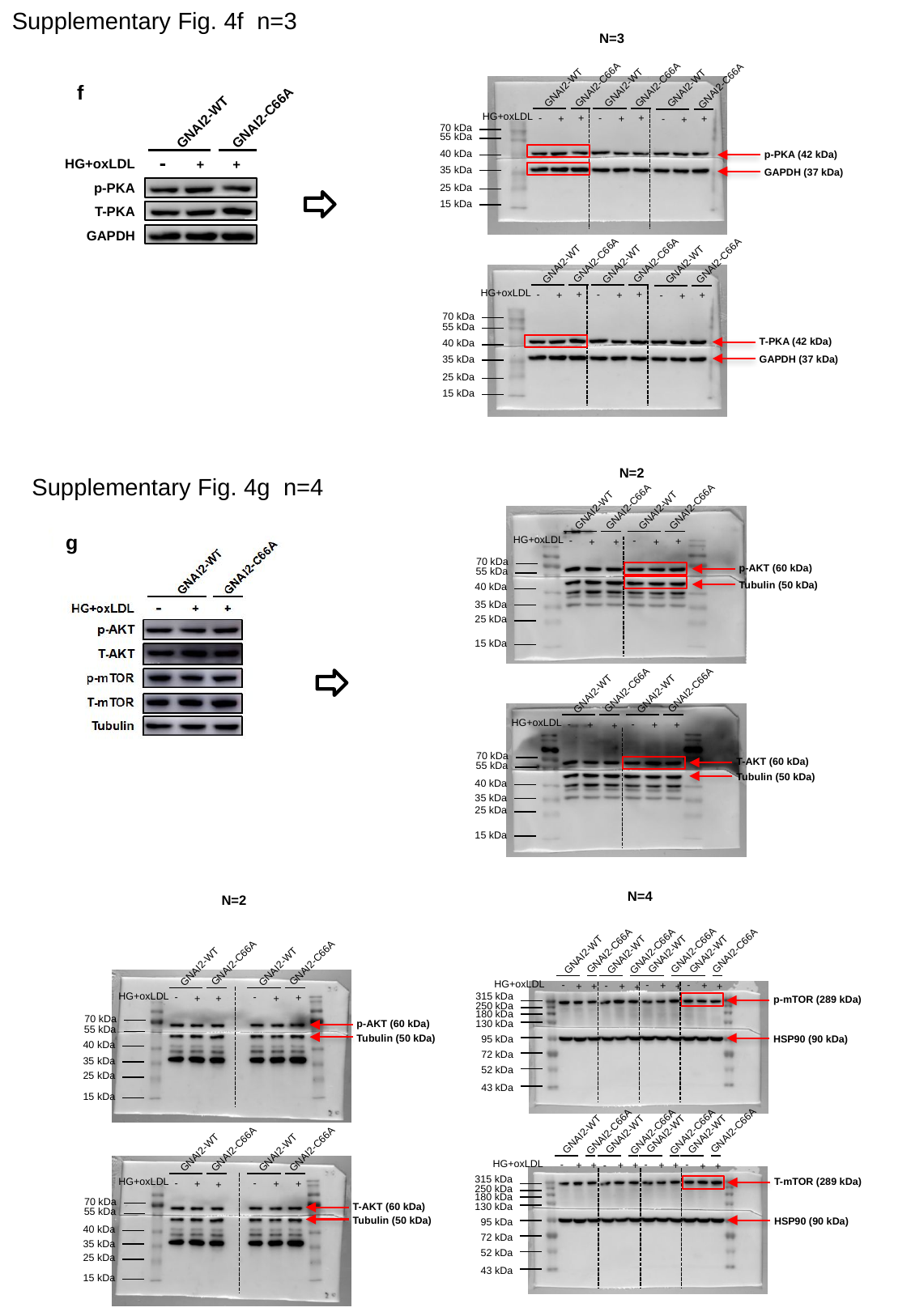

Supplementary Fig. 4f n=3
N=3
GNAI2-C66A
GNAI2-C66A
GNAI2-C66A
GNAI2-WT
GNAI2-WT
f
GNAI2-WT
g
GNAI2-C66A
GNAI2-WT
-
HG+oxLDL
+
+
p-PKA
T-PKA
GAPDH
HG+oxLDL
-
+
-
+
-
+
+
+
+
70 kDa
55 kDa
40 kDa
p-PKA (42 kDa)
35 kDa
GAPDH (37 kDa)
25 kDa
15 kDa
GNAI2-C66A
GNAI2-C66A
GNAI2-C66A
GNAI2-WT
GNAI2-WT
GNAI2-WT
HG+oxLDL
-
+
-
+
-
+
+
+
+
70 kDa
55 kDa
T-PKA (42 kDa)
40 kDa
35 kDa
GAPDH (37 kDa)
25 kDa
15 kDa
N=2
Supplementary Fig. 4g n=4
GNAI2-C66A
GNAI2-C66A
GNAI2-WT
GNAI2-WT
g
HG+oxLDL
-
-
+
+
+
+
70 kDa
p-AKT (60 kDa)
55 kDa
Tubulin (50 kDa)
40 kDa
35 kDa
25 kDa
15 kDa
GNAI2-C66A
GNAI2-C66A
GNAI2-WT
GNAI2-WT
HG+oxLDL
-
-
+
+
+
+
70 kDa
T-AKT (60 kDa)
55 kDa
Tubulin (50 kDa)
40 kDa
35 kDa
25 kDa
15 kDa
N=4
N=2
GNAI2-C66A
GNAI2-C66A
GNAI2-C66A
GNAI2-C66A
GNAI2-WT
GNAI2-WT
GNAI2-WT
GNAI2-WT
GNAI2-C66A
GNAI2-C66A
GNAI2-WT
GNAI2-WT
HG+oxLDL
-
-
-
-
+
+
+
+
+
+
+
+
HG+oxLDL
315 kDa
-
-
+
+
+
+
p-mTOR (289 kDa)
250 kDa
180 kDa
70 kDa
p-AKT (60 kDa)
130 kDa
55 kDa
Tubulin (50 kDa)
95 kDa
HSP90 (90 kDa)
40 kDa
72 kDa
35 kDa
52 kDa
25 kDa
43 kDa
15 kDa
GNAI2-C66A
GNAI2-C66A
GNAI2-C66A
GNAI2-C66A
GNAI2-WT
GNAI2-WT
GNAI2-WT
GNAI2-WT
GNAI2-C66A
GNAI2-C66A
GNAI2-WT
GNAI2-WT
HG+oxLDL
-
-
-
+
+
-
+
+
+
+
+
+
315 kDa
T-mTOR (289 kDa)
HG+oxLDL
-
-
+
+
+
+
250 kDa
180 kDa
70 kDa
T-AKT (60 kDa)
130 kDa
55 kDa
Tubulin (50 kDa)
HSP90 (90 kDa)
95 kDa
40 kDa
72 kDa
35 kDa
52 kDa
25 kDa
43 kDa
15 kDa

## Slide 36
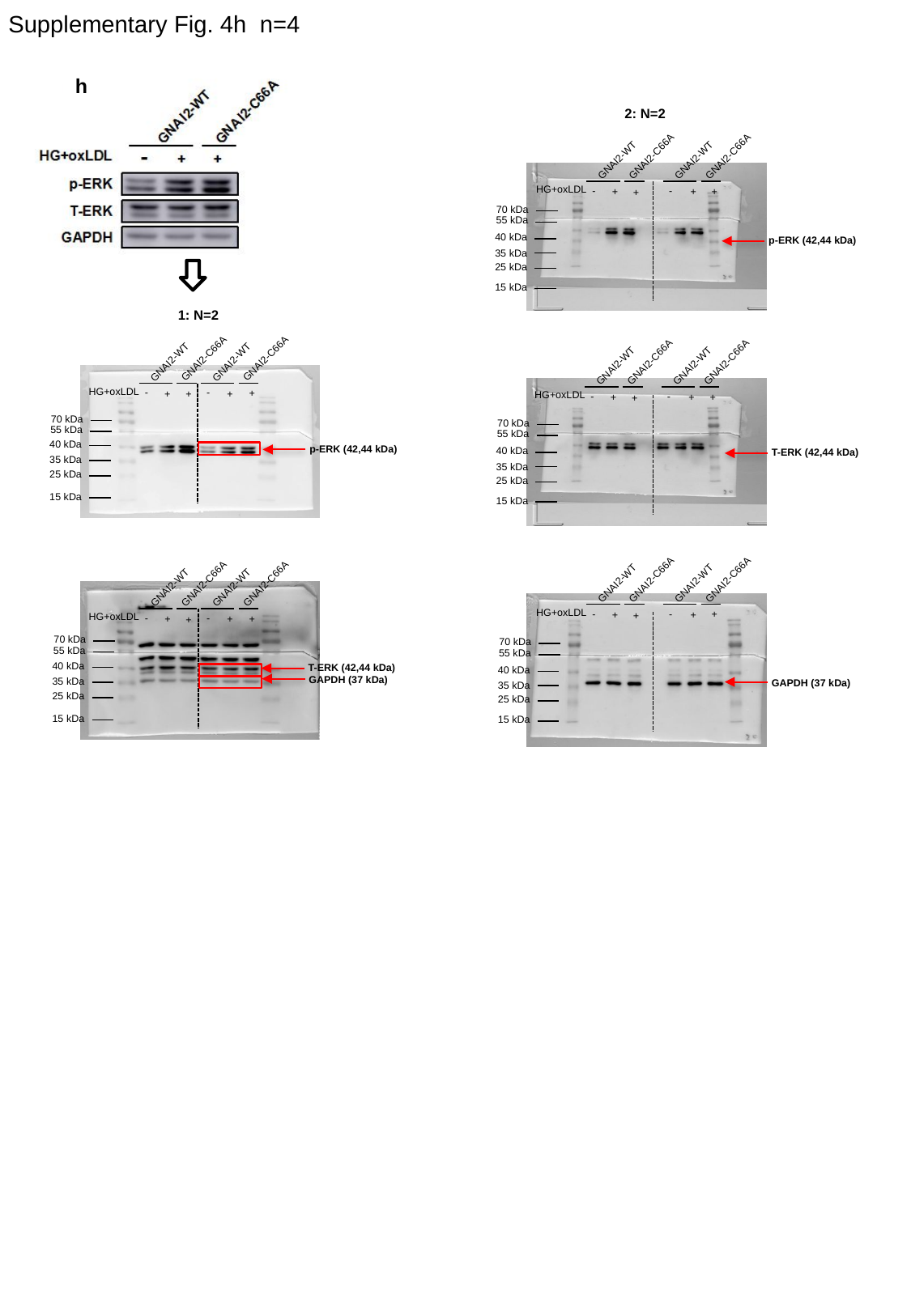

Supplementary Fig. 4h n=4
h
2: N=2
GNAI2-C66A
GNAI2-C66A
GNAI2-WT
GNAI2-WT
HG+oxLDL
-
-
+
+
+
+
70 kDa
55 kDa
40 kDa
p-ERK (42,44 kDa)
35 kDa
25 kDa
15 kDa
1: N=2
GNAI2-C66A
GNAI2-C66A
GNAI2-WT
GNAI2-WT
GNAI2-C66A
GNAI2-C66A
GNAI2-WT
GNAI2-WT
HG+oxLDL
-
-
+
+
+
+
HG+oxLDL
-
-
+
+
+
+
70 kDa
70 kDa
55 kDa
55 kDa
40 kDa
p-ERK (42,44 kDa)
40 kDa
T-ERK (42,44 kDa)
35 kDa
35 kDa
25 kDa
25 kDa
15 kDa
15 kDa
GNAI2-C66A
GNAI2-C66A
GNAI2-WT
GNAI2-WT
GNAI2-C66A
GNAI2-C66A
GNAI2-WT
GNAI2-WT
HG+oxLDL
-
-
+
+
+
+
HG+oxLDL
-
-
+
+
+
+
70 kDa
70 kDa
55 kDa
55 kDa
40 kDa
T-ERK (42,44 kDa)
40 kDa
GAPDH (37 kDa)
35 kDa
GAPDH (37 kDa)
35 kDa
25 kDa
25 kDa
15 kDa
15 kDa

## Slide 37
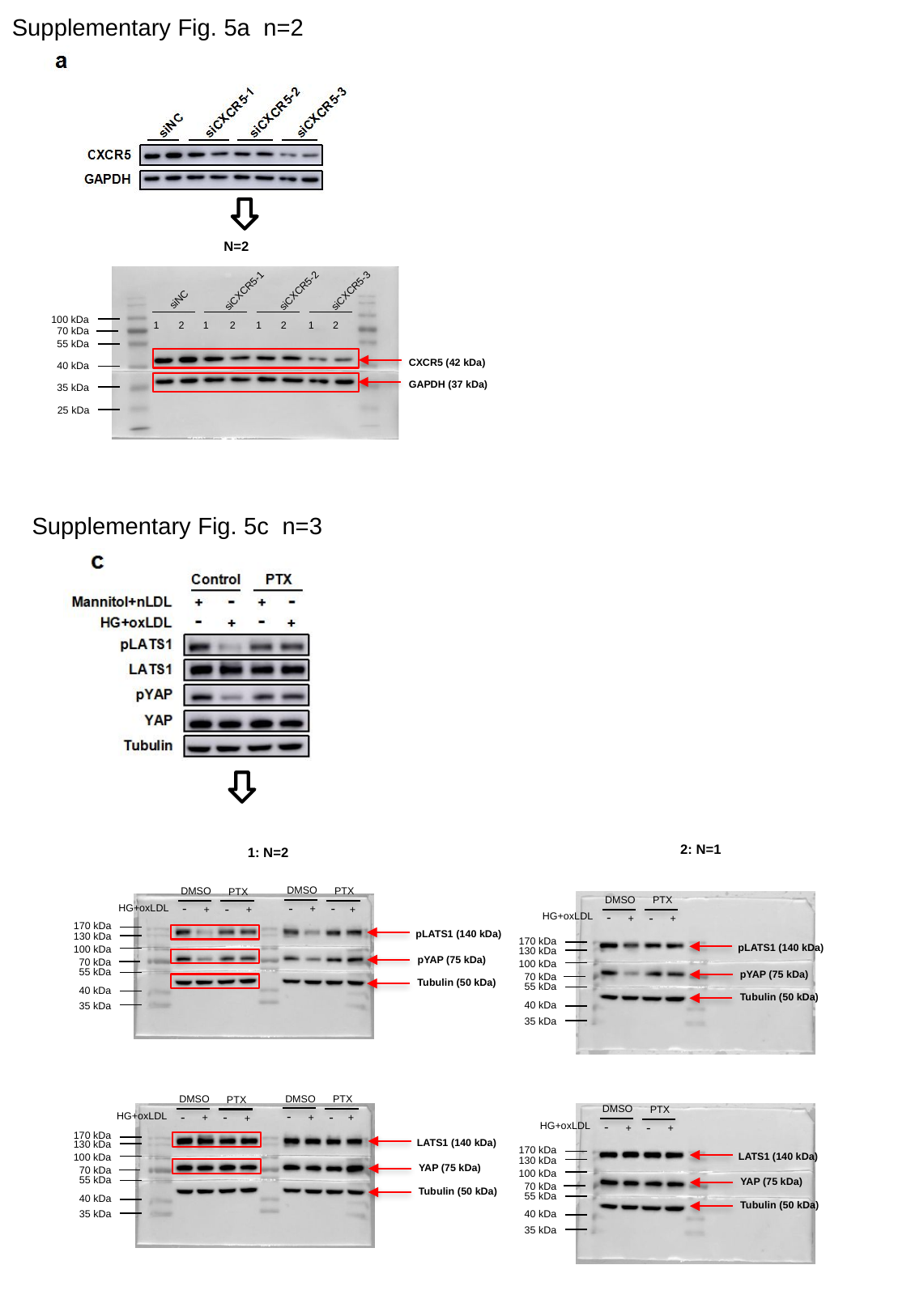

Supplementary Fig. 5a n=2
N=2
siCXCR5-1
siCXCR5-2
siCXCR5-3
siNC
100 kDa
1
2
1
2
1
2
1
2
70 kDa
55 kDa
CXCR5 (42 kDa)
40 kDa
GAPDH (37 kDa)
35 kDa
25 kDa
Supplementary Fig. 5c n=3
2: N=1
1: N=2
DMSO
PTX
DMSO
PTX
DMSO
PTX
-
-
-
-
HG+oxLDL
+
+
+
+
-
-
HG+oxLDL
+
+
170 kDa
pLATS1 (140 kDa)
130 kDa
170 kDa
pLATS1 (140 kDa)
100 kDa
130 kDa
pYAP (75 kDa)
70 kDa
100 kDa
55 kDa
pYAP (75 kDa)
70 kDa
Tubulin (50 kDa)
55 kDa
40 kDa
Tubulin (50 kDa)
40 kDa
35 kDa
35 kDa
DMSO
PTX
DMSO
PTX
DMSO
PTX
-
-
-
-
HG+oxLDL
+
+
+
+
-
-
HG+oxLDL
+
+
170 kDa
LATS1 (140 kDa)
130 kDa
170 kDa
LATS1 (140 kDa)
100 kDa
130 kDa
YAP (75 kDa)
70 kDa
100 kDa
55 kDa
YAP (75 kDa)
70 kDa
Tubulin (50 kDa)
55 kDa
40 kDa
Tubulin (50 kDa)
35 kDa
40 kDa
35 kDa

## Slide 38
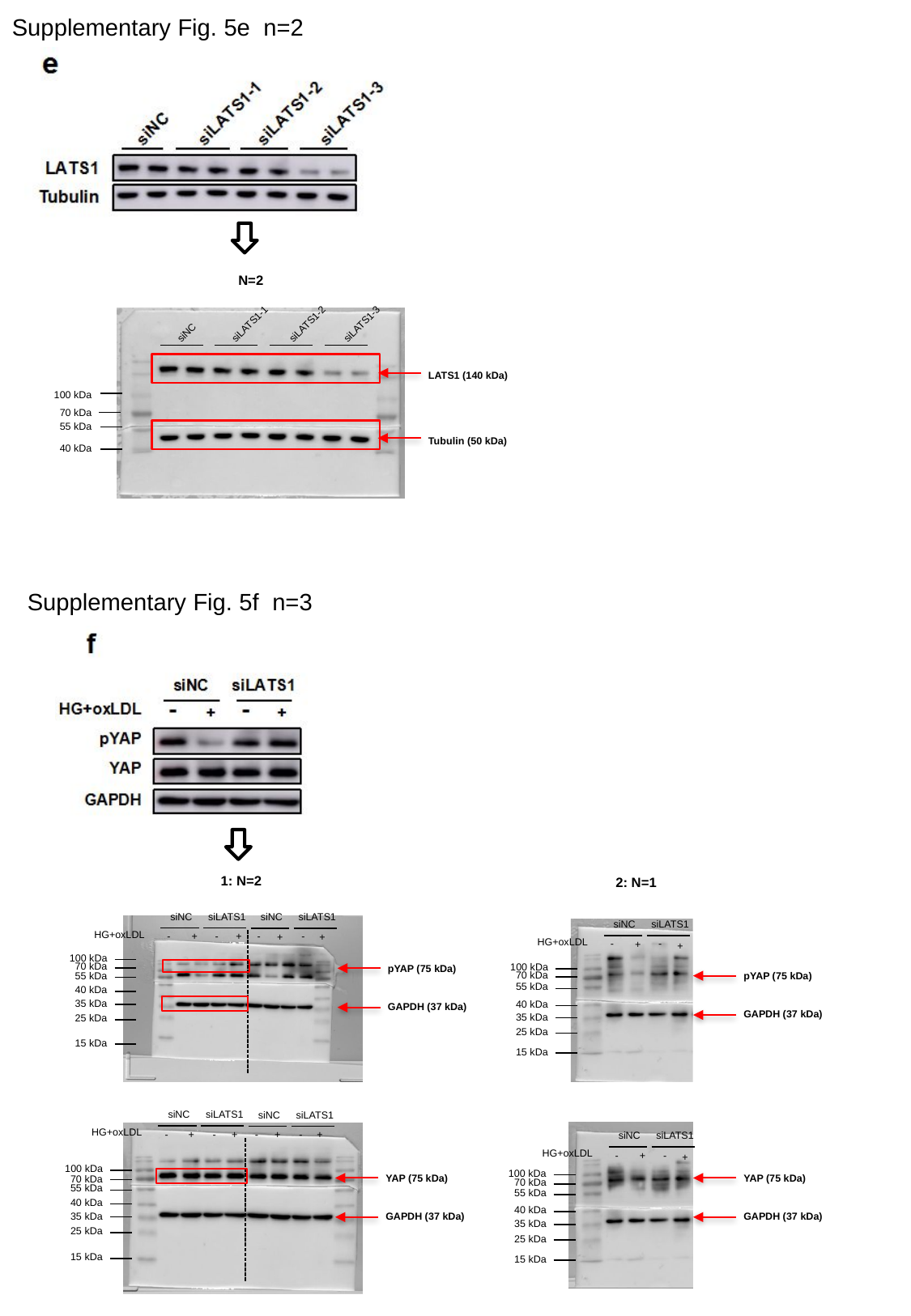

Supplementary Fig. 5e n=2
N=2
siLATS1-1
siLATS1-2
siLATS1-3
siNC
LATS1 (140 kDa)
100 kDa
70 kDa
55 kDa
Tubulin (50 kDa)
40 kDa
Supplementary Fig. 5f n=3
1: N=2
2: N=1
siNC
siLATS1
siNC
siLATS1
siNC
siLATS1
HG+oxLDL
-
-
-
-
+
+
+
+
HG+oxLDL
-
-
+
+
100 kDa
70 kDa
100 kDa
pYAP (75 kDa)
70 kDa
55 kDa
pYAP (75 kDa)
55 kDa
40 kDa
35 kDa
40 kDa
GAPDH (37 kDa)
GAPDH (37 kDa)
35 kDa
25 kDa
25 kDa
15 kDa
15 kDa
siNC
siLATS1
siNC
siLATS1
HG+oxLDL
-
-
-
-
+
+
+
+
siNC
siLATS1
HG+oxLDL
-
-
+
+
100 kDa
100 kDa
YAP (75 kDa)
YAP (75 kDa)
70 kDa
70 kDa
55 kDa
55 kDa
40 kDa
40 kDa
GAPDH (37 kDa)
GAPDH (37 kDa)
35 kDa
35 kDa
25 kDa
25 kDa
15 kDa
15 kDa

## Slide 39
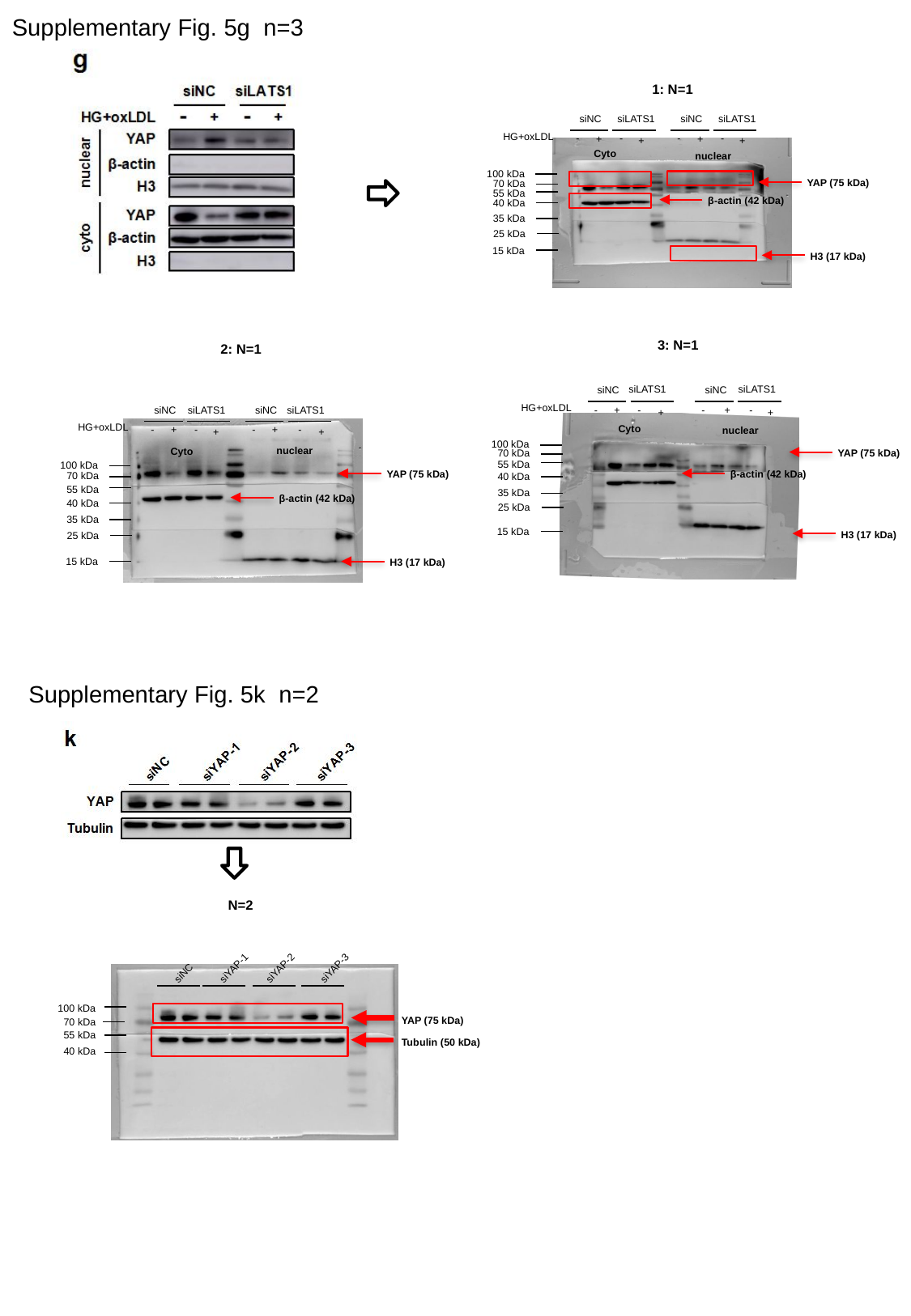

Supplementary Fig. 5g n=3
1: N=1
siNC
siLATS1
siNC
siLATS1
HG+oxLDL
-
-
-
-
+
+
+
+
Cyto
nuclear
100 kDa
YAP (75 kDa)
70 kDa
55 kDa
β-actin (42 kDa)
40 kDa
35 kDa
25 kDa
15 kDa
H3 (17 kDa)
3: N=1
2: N=1
siLATS1
siLATS1
siNC
siNC
HG+oxLDL
siNC
siNC
siLATS1
siLATS1
-
-
-
 -
+
+
+
+
HG+oxLDL
Cyto
-
-
-
-
+
+
nuclear
+
+
100 kDa
nuclear
Cyto
YAP (75 kDa)
70 kDa
55 kDa
100 kDa
β-actin (42 kDa)
YAP (75 kDa)
70 kDa
40 kDa
55 kDa
35 kDa
β-actin (42 kDa)
40 kDa
25 kDa
35 kDa
15 kDa
H3 (17 kDa)
25 kDa
15 kDa
H3 (17 kDa)
Supplementary Fig. 5k n=2
N=2
siYAP-1
siYAP-2
siYAP-3
siNC
100 kDa
YAP (75 kDa)
70 kDa
55 kDa
Tubulin (50 kDa)
40 kDa

## Slide 40
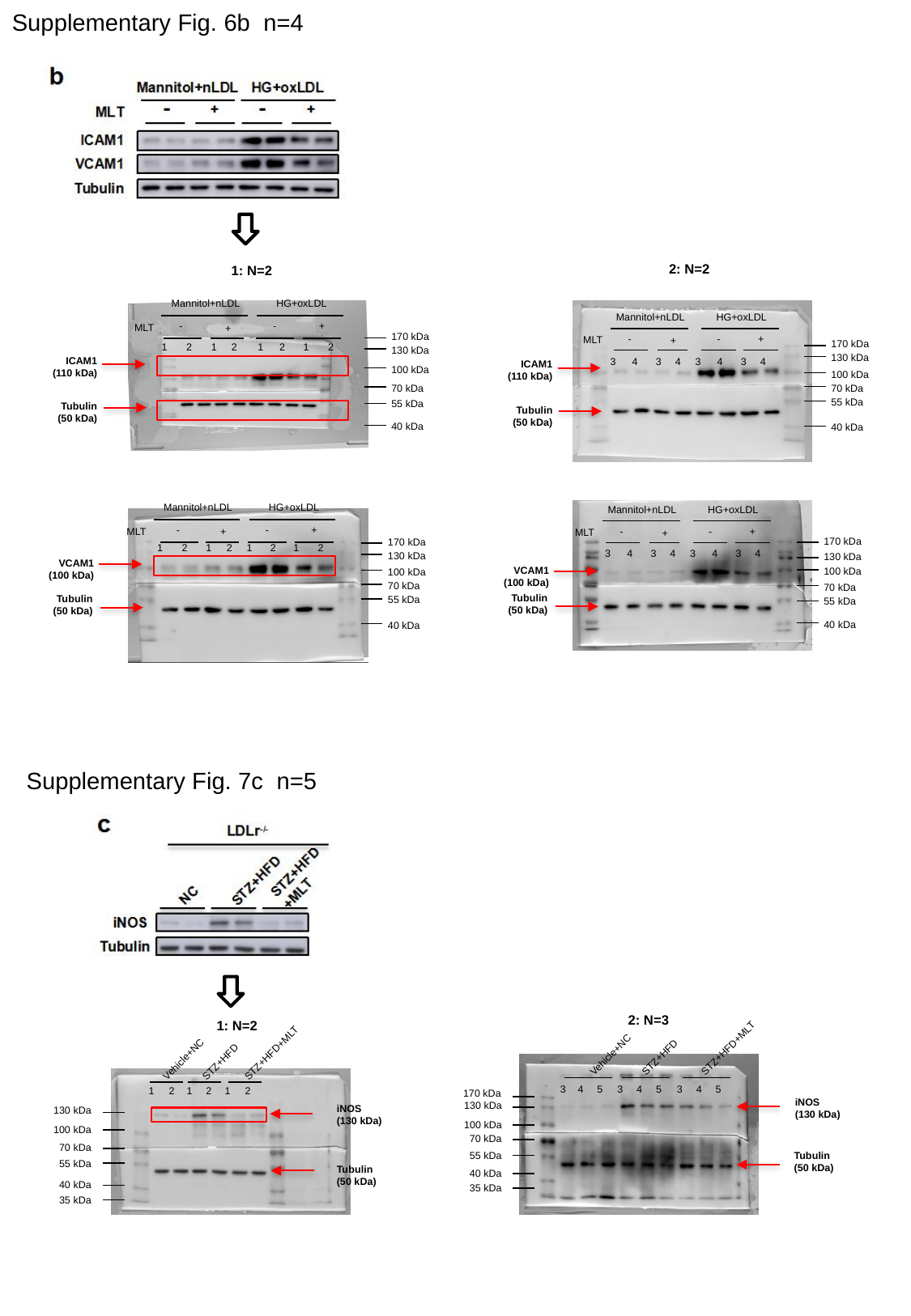

Supplementary Fig. 6b n=4
2: N=2
1: N=2
Mannitol+nLDL
HG+oxLDL
+
-
-
MLT
+
Mannitol+nLDL
HG+oxLDL
+
-
-
MLT
+
170 kDa
170 kDa
1
2
1
2
1
2
1
2
130 kDa
130 kDa
ICAM1
(110 kDa)
3
4
3
4
3
4
3
4
ICAM1
(110 kDa)
100 kDa
100 kDa
70 kDa
70 kDa
55 kDa
55 kDa
Tubulin
(50 kDa)
Tubulin
(50 kDa)
40 kDa
40 kDa
Mannitol+nLDL
HG+oxLDL
+
-
-
MLT
+
Mannitol+nLDL
HG+oxLDL
+
-
-
MLT
+
170 kDa
170 kDa
1
2
1
2
1
2
1
2
3
4
3
4
3
4
3
4
130 kDa
130 kDa
VCAM1
(100 kDa)
VCAM1
(100 kDa)
100 kDa
100 kDa
70 kDa
70 kDa
Tubulin
(50 kDa)
Tubulin
(50 kDa)
55 kDa
55 kDa
40 kDa
40 kDa
Supplementary Fig. 7c n=5
2: N=3
1: N=2
STZ+HFD+MLT
STZ+HFD+MLT
Vehicle+NC
STZ+HFD
Vehicle+NC
STZ+HFD
3
4
5
3
4
5
3
4
5
1
2
1
2
1
2
170 kDa
iNOS
(130 kDa)
130 kDa
iNOS
(130 kDa)
130 kDa
100 kDa
100 kDa
70 kDa
70 kDa
Tubulin
(50 kDa)
55 kDa
55 kDa
Tubulin
(50 kDa)
40 kDa
40 kDa
35 kDa
35 kDa
